# From erbium(iii) to samarium(iii): generalized photomodulation of NIR to red lanthanide luminescence with a DTE ligand and its versatile role in the quenching processes

**DOI:** 10.1039/d5sc07174g

**Published:** 2025-10-27

**Authors:** Tuan-Anh Phan, Frédéric Gendron, Salauat Kiraev, Olivier Galangau, Yoann Fréroux, Hassan Al Sabea, Cédric Mittelheisser, Marie Dallon, Aude Bouchet, François Riobé, Remi Métivier, Michel Sliwa, Boris Le Guennic, Olivier Maury, Akos Banyasz, Bogdan Marekha, Lucie Norel, Stéphane Rigaut

**Affiliations:** a Univ Rennes, CNRS, ISCR (Institut des Sciences Chimiques de Rennes) – UMR 6226 F-35000 Rennes France stephane.rigaut@univ-rennes1.fr lucie.norel@univ-rennes1.fr; b ENS de Lyon, CNRS, LCH, UMR 5182 69342 Lyon cedex 07 France olivier.maury@ens-lyon.fr; c Univ. Bordeaux, CNRS, Bordeaux INP, ICMCB, UMR 5026 F-33600 Pessac France; d Univ. Lille, CNRS, UMR 8516, LASIRE Laboratoire de Spectroscopie pour les Interactions, la Réactivité et l'Environnement Lille F59 000 France; e LOB, CNRS, INSERM, École Polytechnique, Institut Polytechnique de Paris 91120 Palaiseau France; f UMR CNRS 8531-PPSM, ENS Paris-Saclay, Université Paris-Saclay 94235 Cachan France

## Abstract

In this article, we report that a stable molecular system combining three DTE-β-diketonate units, a phenanthroline ligand and a lanthanide ion provides, for an unprecedented panel of lanthanide(iii) ions, efficient luminescence together with an emission quenching process under UV light with very high efficiency. Indeed, this process is applicable to visible emitters (samarium(iii) and europium(iii)), and more importantly to different NIR emitters (ytterbium(iii), neodymium(iii) and erbium(iii)), and it can be reversed under visible light irradiation in a highly repeatable way. The luminescence sensitizing and quenching mechanisms were further investigated with experimental and theoretical tools to decipher the peculiar and complex role of the DTE-β-diketonate units. In particular, these studies reveal the existence (i) of a very high cyclization quantum yield of the open isomer in the presence of oxygen with a triplet mediated mechanism depending on the lanthanide ion nature, and (ii) of a dark triplet state located on the closed isomer explaining the quenching of the NIR and visible emitters.

## Abstract 2

We report that a molecular system combining three DTE-β-diketonate units, a phenanthroline ligand and a lanthanide ion provides, for an unprecedented panel of ions, efficient luminescence together with an emission quenching process under UV light.

## Graphical abstract

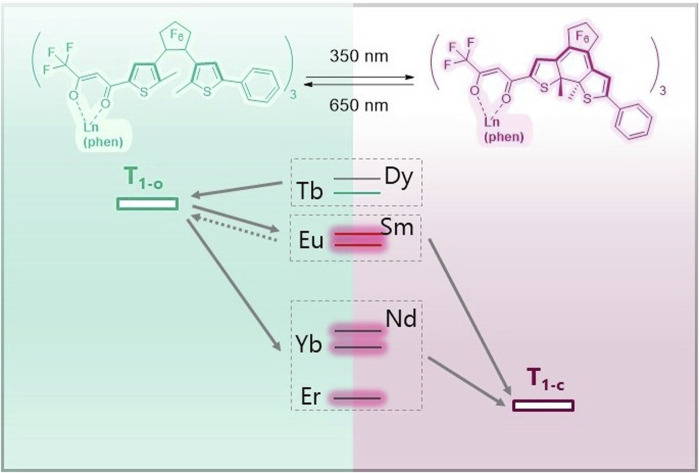

## Introduction

Lanthanide (Ln) β-diketonates are the most intensively investigated rare-earth coordination compounds due (1) to their facile synthesis and commercial availability, and (2) to their possible applications in several fields, particularly in biomedical imaging or in molecular materials with optical fiber lasers, sensors, anticounterfeiting systems or organic light emitting diodes (OLEDs).^1–6^ Such complexes are luminescent *via* indirect excitation through organic ligands, and their emission color can be tuned across the visible and near-infrared (NIR) spectrum by changing the nature of the lanthanide ion. This is contingent upon the excited states of β- diketonate ligand displaying appropriate energy levels above the emissive states of the suitable Ln^3+^ ion to enable efficient sensitization.^7^ Among all β-diketonate ligands, 2-thenoyltrifluoroacetonate (tta^−^) is the most popular owing to the photoluminescence properties of the resulting neutral tris(β-diketonate) complex [Eu(tta)_3_(phen)] (phen = 1,10-phenanthroline) producing, for instance, luminescent resins^8^ or liquid crystals.^9^

For all the above mentioned applications, and also for purely fundamental reasons, it occurred to us that the remote emission control of Ln^3+^ β-diketonate complexes with an external stimulus, *i.e.* an electron transfer or a photon irradiation, was very appealing, and particularly in the unexplored NIR domain.^10^ In that regard, we reported in 2011 the first electrochemical switching of NIR emission with an Yb^3+^ ion associated with an organometallic redox-active ruthenium complex,^11,12^ and in 2019 the first reversible photochemical switching with a related organometallic complex equipped with photochromic β-diketonate-dithienylethene (DTE) ligands.^13^ This ligand can be reversibly switched from a non-conjugated open form to a conjugated one upon UV or vis irradiations leading to a 98% quenching efficiency of the initial maximum emission intensity in the closed conjugated form. Later, as their tta analogues with the rigid phenanthroline ligand are well known for their efficient emissions,^14,15^ we extended this concept to simpler [Ln(β-diketonate-DTE)_3_(phen)] complexes for anticounterfeiting materials with innovative responses,^16^ still with the Yb^3+^ ion, and also with the widely studied red emissive Eu^3+^ ion.^17^ However, despite the successful emission photoswitching upon light irradiation, the underlying mechanisms are not clearly understood. Several studies with similar photochromic switching of Ln^3+^ emission have been published with Eu^3+^ and Tb^3+^ ions, and intramolecular fluorescence resonance energy transfer (FRET) or electron transfer involving the conjugated DTE form are often assumed.^17,18^ However, in the case of Yb^3+^, the mechanism is far from trivial since the resonance condition is not satisfied with an emission occurring at around 1000 nm (*ca.* 10 000 cm^−1^) and a closed DTE isomer absorption at *ca.* 630 nm (*ca.* 16 000 cm^−1^). In addition, to the best of our knowledge, ions such as Sm^3+^ and Er^3+^ have never been studied for the switching of their emission while for Dy^3+^ and Nd^3+^, scarce reports^19,20^ exist that rely on photoinduced electron transfer rather than on well reversible, efficient and robust photochromic ligands.^17^ The case of Er^3+^ is (i) of particular interest with regard to its potential applications, given that its emission wavelength (1530 nm) falls within the short wavelength infrared (SWIR) range,^21,22^ and (ii) extremely challenging due to its very low energy lying excited state (6800 cm^−1^), prone to fast non radiative relaxation pathway *via* multiphonon vibronic quenching with OH, NH and CH oscillators.^23–25^

In this work, in order to address the above issues concerning (i) the widening of the scope of emitters and (ii) the understanding of the quenching processes, we extended the emission photomodulation to ions other than Eu^3+^ and Yb^3+^. Indeed, the above mentioned [Ln(β-diketonate-DTE)_3_(phen)] complex can be prepared for a range of lanthanide centers with various emission colors in order to observe which ions preserve or not ON–OFF emission switching. Since each emitter exhibits well defined metal centered excited states, this systematic approach also allows to decipher which mechanisms can contribute to sensitization or to quenching in each case. Therefore, we report in this article (i) six new systems with Nd^3+^, Sm^3+^, Gd^3+^, Tb^3+^, Dy^3+^, Er^3+^ ions, along with (ii) the photophysical characterizations of this unique array of ions, also including the Yb^3+^ and Eu^3+^ analogues, with emission quenching studies that display excellent reversibility upon multiple ON–OFF emission cycling. In addition, we describe deeper investigations to decipher the system behavior including (iii) photokinetic studies of photochromism under stationary irradiation combined with femto to microsecond transient absorption experiments highlighting the existence of competitive closing mechanisms, and (iv) an unprecedented rationalization of the emission quenching with the help of theoretical calculations beyond the often-tentative FRET explanation. Importantly, this study clarifies why such a system displays an emission quenching that remains efficient from the visible (Sm^3+^, Eu^3+^) to the NIR (Yb^3+^, Nd^3+^, Er^3+^) ranges and will contribute to further design novel efficient systems and applications with lanthanide ions.

## Results and discussion

### Complex synthesis

**Scheme 1:**
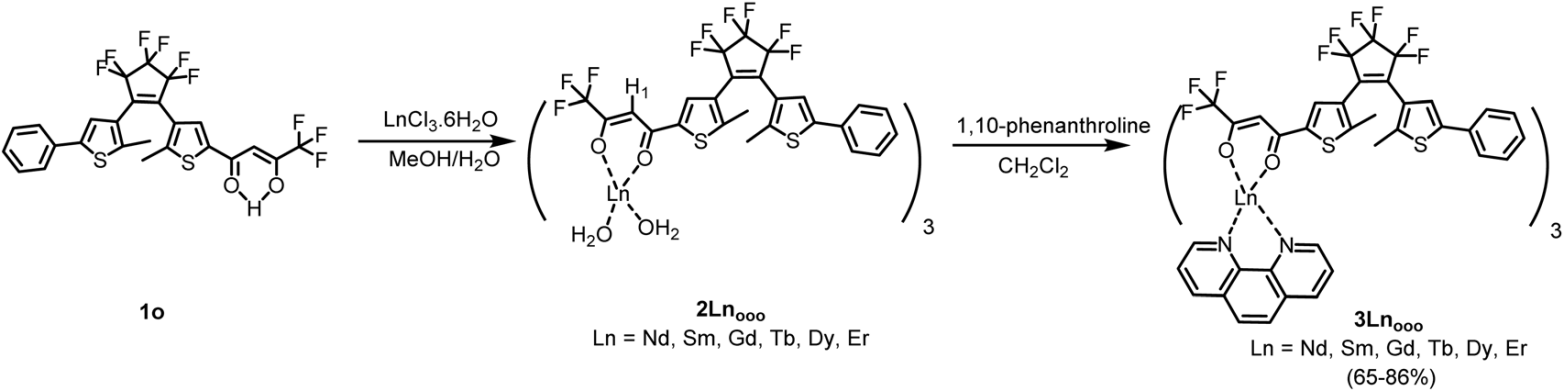
Synthetic pathway yielding new complexes 3L_nooo_ (Ln = Nd, Sm, Gd, Tb, Dy, Er).

For each lanthanide ion, the synthesis of the new compounds was conducted in two steps as previously reported for Eu^3+^ and Yb^3+^ ions.^16^ Thus, as described in Scheme 1, complexation of three equivalents of DTE-β-diketone 1o in its open form with the desired lanthanide ion (Ln^3+^ = Nd^3+^, Sm^3+^, Gd^3+^, Tb^3+^, Dy^3+^, Er^3+^) afforded the bis-aqua precursors [Ln(1o)_3_·2H_2_O] (2Ln_ooo_). The synthesis of the final complexes 3Ln_ooo_, bearing three DTE units in their open state, was readily achieved with further coordination of phenanthroline ligand in CH_2_Cl_2_ (see SI). Except in the case of 3Gd_ooo_, informative ^1^H NMR spectra, displaying the expected paramagnetic (pseudo-contact) shifts, were obtained with the diketonate methine protons experiencing the largest shifts due to its proximity to the paramagnetic center (see SI). The phenanthroline protons experience as well large shifts of opposite sign compared to the methine protons of the DTE ligands. Also, ^19^F NMR spectra showed signals corresponding to the CF_3_ group and to the DTE central and external CF_2_ groups of the diketonate chelators. Note that a single and well resolved set of signals appears for the three β-diketonate ligands indicating a rapid exchange between them, faster than the time scale of the NMR experiment (<10 μs).^26,27^

### Absorption spectra and photochromism of 3Ln_ooo_

**Fig. 1:**
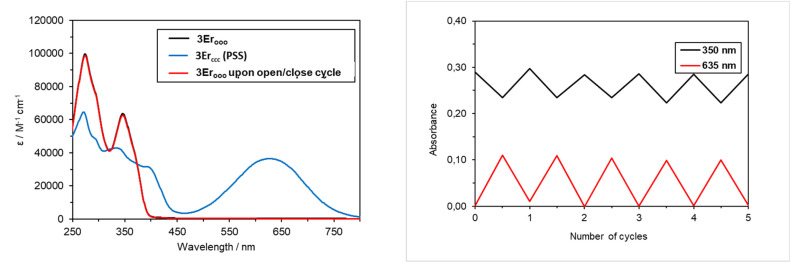
(Left) Absorption spectra of 3Er_ooo_ in CH_2_Cl_2_ solutions ([c] ∼ 5 × 10^−6^ mol L^−1^, blue line) and upon UV irradiation (*λ* = 350 nm) to PSS (green line). The initial spectrum was fully recovered after irradiation at *λ* = 650 nm (red line). (right) Reversibility of the process followed by optical density (OD) at 350 and 635 nm for 3Er_ooo_ over 5 cycles.

**Scheme 2:**
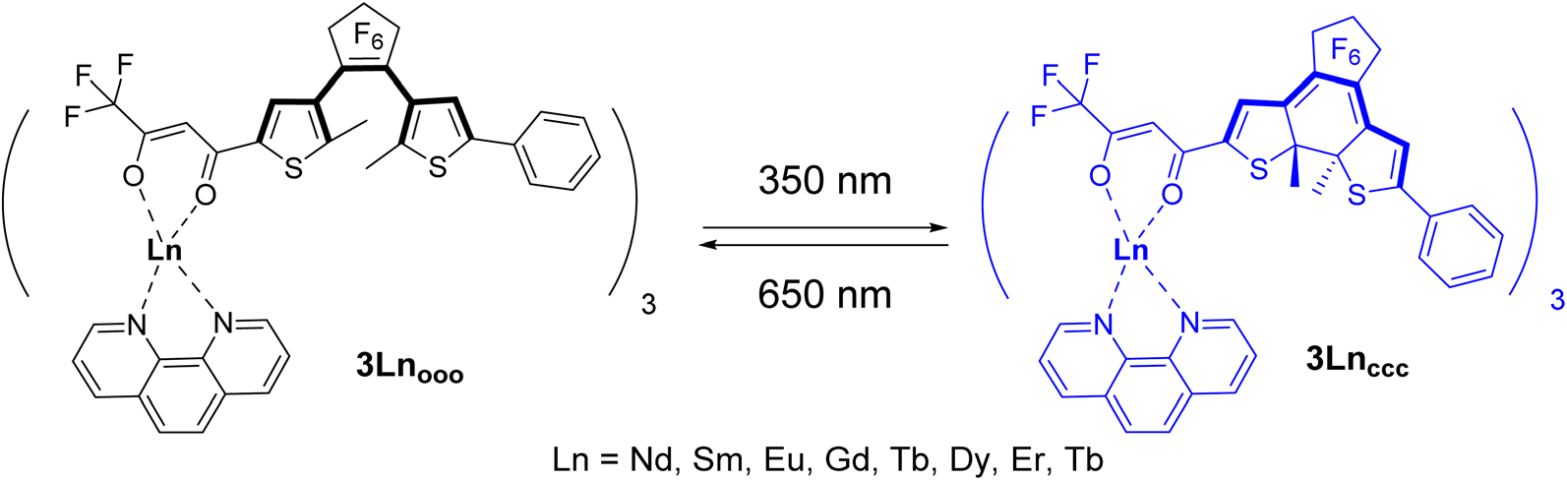
Photochromic switching between the 3Ln_ooo_ and 3Ln_ccc_ complexes upon UV (*λ* = 350 nm) and visible (*λ* = 650 nm) light irradiation, respectively.

The absorption spectra of all new complexes were recorded in diluted CH_2_Cl_2_ solutions ([c] ∼10^−5^ mol L^−1^). They are nearly identical and similar to those of the previously studied Eu^3+^/Yb^3+^ complexes (see Table 1, Fig. 1 and S9–14). The strong absorption bands at *ca. λ*_max_ = 274 nm and *λ*_max_ = 350 nm are assigned to π–π* transitions of the open DTE ligand, probably overlapping with the transitions based on the phenanthroline ligand.^28^ Note that the latter has an absorption maximum at 264 nm (*ε* = 31 000 M^−1^ cm^−1^) and no contribution at wavelengths longer than 330 nm. Upon irradiation at *λ* = 350 nm of solutions of all 3Ln_ooo_ complexes, all spectra display characteristic DTE photochromic behaviour (Scheme 2) accompanied by a deep blue coloration of the solutions. More specifically, while reaching the photostationary state (PSS), a decrease in absorption intensities of the two π–π* transitions is observed, concomitantly with the rising of a new broad absorption at *ca. λ*_max_ = 630 nm along with a shoulder at *ca.* 390 nm. The new main band is ascribed to an intra-ligand (IL) transition centered on the cyclized DTE moiety. These solutions can be bleached back to the pure colorless open forms under visible light irradiation (*λ* = 650 nm), as attested by the quantitative recovering of the initial spectra for all complexes. Furthermore, the reversibility of the process monitored by following the absorption changes at 350 and 635 nm shows the good fatigue resistance of the compounds over several cycles (see Fig. 1 for 3Er_ooo_). Previous studies on 3Eu_ooo_ and 3Yb_ooo_ with the help of ^1^H NMR spectroscopy revealed the PSS composition.^16^ Photochromic conversion was found to be almost complete for both complexes with the reaching of *ca.* 92% of overall closed isomers. This value corresponds to 80% of the fully closed complex 3Ln_ccc_ and 20% of 3Ln_occ_ (Ln = Yb, Eu) with two closed photochromic units, obtained through sequential ring-closures ooo → ooc → occ → ccc. This tendency was confirmed with 3Nd_ooo_, 3Sm_ooo_ and 3Er_ooo_ displaying 93%, 89% and 87% overall conversions, respectively (see Fig. S15–17). The precise and consistent conversion to 78% of 3Nd_ccc_ and 22% of 3Nd_occ_ could be determined for Nd^3+^ only, owing the larger broadening with the other ions, preventing a clear distinction of the four species. Note that the identical absorption spectra of the new complexes, both in their initial and photostationary states, confirm (i) that the conversions are similar for all complexes (including 3Tb_ooo_, 3Gd_ooo_ and 3Dy_ooo_), and (ii) give an estimation of the absorption coefficient of one closed DTE unit in the complexes for their transition at *λ*_max_ ∼630 nm (Table 1). The latter coefficient appears to be consistent within the series of complexes and with the isolated 1c, again supporting NMR conversions along with the apparent decoupling of the DTE units in the fundamental state.

**Table 1:** UV/vis data of 3Ln complexes in CH_2_Cl_2_ solutions^a^

	Open isomer 3Ln_ooo_		One closed DTE in 3Ln_ccc_/3Ln_occ_	
	*λ*_max_ (nm)	*ε* (M^−1^ cm^−1^)	*λ*_max_ (nm)	*ε* (M^−1^ cm^−1^)
1o	280, 340	24 400, 19 600	622	13 400
3Eu	273, 348	119 200, 61 400	630	13 000
3Yb	273, 346	98 200, 62 800	627	12 400
3Nd	274, 350	105 200, 64 100	630	14 800
3Sm	274, 349	100 600, 64 700	630	13 700
3Gd	274, 348	99 400, 61 500	631	12 700
3Tb	274, 348	101 400, 65 900	631	15 100
3Dy	273, 347	100 500, 66 900	630	14 000
3Er	274, 346	99 800, 63 700	627	13 200

a The molar absorption coefficient of one closed DTE unit is calculated using the PSS composition corresponding to 92% of the closed isomer.

**Fig. 2:**
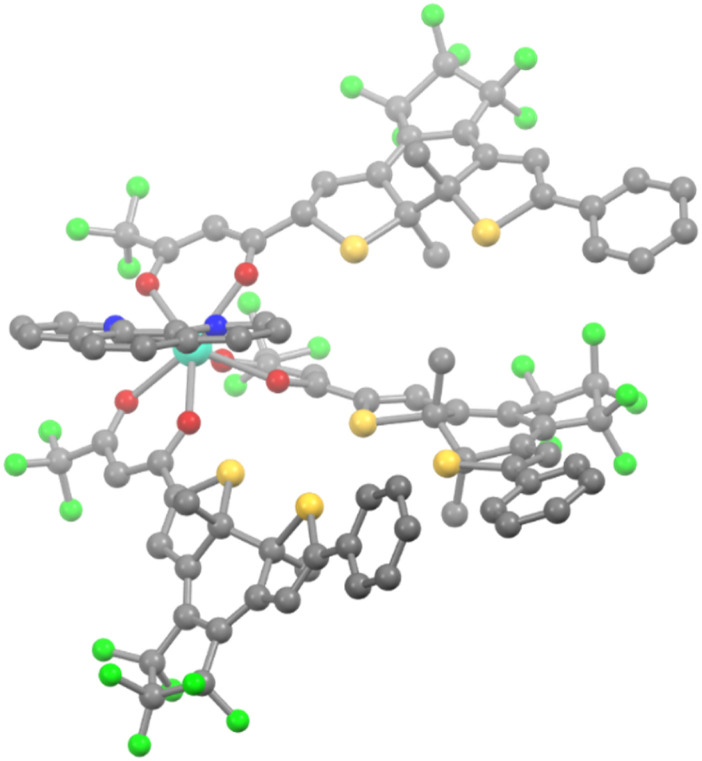
View of the SC-XRD structure of 3Dy_ccc_ (H atoms are omitted for clarity; grey, yellow, blue, green red and light blue spheres represent C, S, N, F O and Dy atoms, respectively).

Further proof of the formation of 3Ln_ccc_ with high photoconversion was provided by the obtention of good quality crystals suitable for X-ray structure determination in the case of 3Yb_ccc_^16^ and 3Dy_ccc_ (Fig. 2). Both were obtained by slow evaporation of dichloromethane solutions after irradiation to the PSS and concentration under vacuum. 3Dy_ccc_ was found isostructural to 3Yb_ccc_ (see SI). We can stress here that the geometrical isomer having all three closed β-diketonate ligands pointing in the same direction is the one crystallizing, as previously observed for similar complexes.^29^ This arrangement allows to accommodate the three photochromic ligands around the metal center without strong distortion of the coordination sphere, that remains close to a square antiprism, even taking into account lanthanide contraction.

### Isomerisation photokinetic and photodynamics studies

Further photokinetic measurements of 3Ln_ooo_ were performed at 365 nm, under air at 20 °C. The absorption time profile evolutions resulting from photocyclizations, measured at *λ*_max_ of closed DTE absorption band of each species, are markedly different and clearly depend on the nature of the metal (Table 2 and Fig. S18). Indeed, 3Eu_ooo_ exhibits the smallest value of *t*^Eu^_PSS_ (750 s), defined as the time at which the molecular systems have reached the photostationary state arbitrarily fixed to 95% of the asymptote value. Conversely, 3Er_ooo_ displays the highest *t*^Er^_PSS_ (3000 s), making 3Er_ooo_ the slowest compound of the family. The absorption time-profiles were further analyzed numerically, based on the classical photochromic differential equations, to decipher the photochromic quantum yields (ESI).^13,30^ The determination of the different quantum yields, upon 365 nm light irradiation, are in line with a stepwise cyclization process, where three distinct quantum yields could be evaluated. More precisely, throughout the entire series, cyclization quantum yields followed *ϕ*_o/c_^1^ ≫ *ϕ*_o/c_^2^ ≥ *ϕ*_o/c_^3^, with unexpected impressive first cyclisation quantum yield *ϕ*_o/c_^1^ about 0.9–1.0 for 3Eu_ooo_, 3Sm_ooo_ and 3Yb_ooo_. The stepwise process agrees with the fact that the cyclization of a first and a second photochromic subunit may quench the following ones by energy transfer from an excited open form towards a closed form, as usually observed in the case of multiphotochromic compounds.^31^ Note that from the SC-XRD structures of 3Ln_ccc_, short intramolecular distances between closed DTE units are observed with the shortest S–S distance around 4 Å, probably indicative of similar short distances between open and closed units in 3Ln_ooc_, favorable for energy transfer. Unexpectedly, *ϕ*_o/c_^1^ experienced large variations within the family, ranging from 0.43 to 0.99 with no obvious correlation when varying the size or the energy levels of the lanthanide ions. The answer likely lies in the inherently complex nature of lanthanide ions and probably implies a balance of different effects such as ligand exchange rates in the lanthanide coordination sphere, ligand reorganization upon isomerization, ligand-to-ligand energy transfer, and lanthanide-to-ligand energy transfer. Further investigations would be necessary to rationalize this effect that is, despite its interest and novelty, out of the scope of the present study.

**Table 2:** Time to reach the PSS (*t*^Ln^_PSS_), and quantum yields of ring cyclization/cycloreversion obtained from fitting the absorption time-profiles at 20 °C, under air

	3Nd_ooo_	3Sm_ooo_	3Eu_ooo_	3Gd_ooo_	3Tb_ooo_	3Dy_ooo_	3Er_ooo_	3Yb_ooo_
*t* ^Ln^_PSS_ (s)	2380	1000	750	2500	2250	2366	3000	2000
*ϕ*_o/c_^1^ a	0.43	0.87	0.99	0.60	0.75	0.76	0.65	0.98
*ϕ*_o/c_^2^ a	0.20	0.25	0.46	0.13	0.26	0.14	0.10	0.13
*ϕ*_o/c_^3^ a	0.08	0.09	0.23	0.08	0.09	0.08	0.04	0.06
*ϕ*_c/o_^365^	0.006	0.006	0.002	0.001	0.003	0.002	0.002	0.003
*ϕ*_c/o_^577^	0.007	0.006	0.008	0.007	0.006	0.006	0.007	0.007

a Determined upon irradiation at 365 nm. Quantum yield values are given with 10% error.

Finally, in the case of cycloreversion, only one quantum yield per DTE can describe the ring opening process either under UV irradiation at 365 nm (*ϕ*_o/c_^365^) or from the PSS under visible irradiation at 577 nm (*ϕ*_c/o_^577^) (Table 2 and Fig. S19). These cycloreversion quantum yields are small (<1%), as usually observed for DTE compounds, and similar throughout the lanthanide series.

**Fig. 3:**
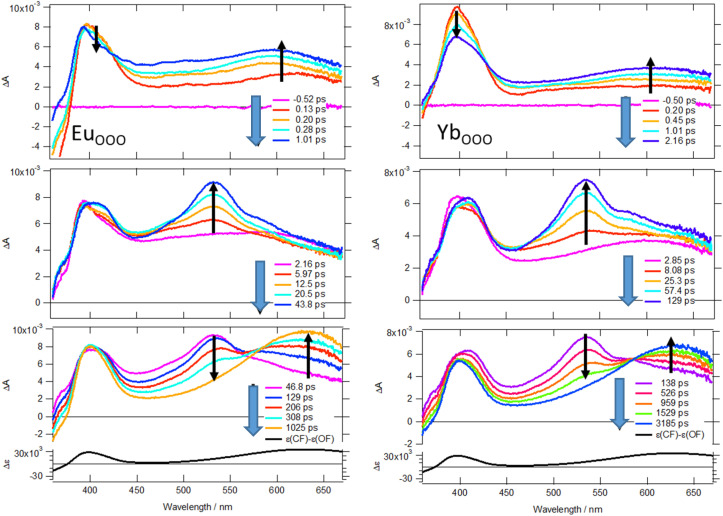
Femtosecond transient difference absorption spectra recorded at different time delays after a femtosecond laser excitation (350 nm) of 3Eu_ooo_ (left) and 3Yb_ooo_ (right).

To get a better insight into the exceptionally high *ϕ*_o/c_^1^ values, we further investigated the photocyclization reaction dynamics of 3Eu_ooo_ and 3Yb_ooo_ using UV-visible femtosecond transient absorption spectroscopy as cyclization for DTE is known to happen in the sub-picosecond to few tens of picosecond time scale from the anti-parallel isomers.^32–35^Fig. 3 shows transient absorption spectra after a 350 nm femtosecond (fs) excitation pump (60 fs FWHM) from fs to nanosecond (ns) for both complexes in CH_2_Cl_2_. Just after the pump (transient spectra at 100 fs, first time-delay panel), a small negative band is observed below 365 nm while the other part is positive with a sharp absorption band at around 395 nm and a broad band covering the 450–670 nm region with a maximum at about 620 nm. The negative band is assigned to ground state bleaching and the positive band with different maxima is ascribable to the excited state absorption (ESA) of the reactive anti-parallel (AP) and usually unreactive parallel (P) DTE isomers. In about 1 ps, the first time-delay panel shows a decrease of ESA at 395 nm and simultaneously the increase of the band at 620 nm. This evolution is assigned to some intramolecular relaxation of the P isomer and formation of the closed form from the AP form (see the differential spectrum between the open- and closed-ring isomers in the last time-delay panel). Then in few tens of picosecond, a new ESA grows (see the second time-delay panel) with a maximum at 535 nm assigned to triplet excited state of remaining open P isomers. Indeed, tens of picosecond time constant is usually interpreted as relaxation of excited singlet state of P isomer by internal conversion and intersystem crossing system with the formation of a triplet excited state.^32,35^ While for classical DTE, the main pathway from the singlet state of the P form is usually internal conversion with no ESA remaining after few hundred of picosecond, in the present case, the interaction with a lanthanide ion causes intersystem crossing to be the main deactivation pathway. A similar quantitative triplet excited state formation was observed for a DTE unit with triplet sensitizer group at the periphery,^36^ but with no evolution in hundred picosecond time scale and a triplet state decaying in microsecond time scale by internal conversion and cyclization with a yield below 0.3 and sensitive to oxygen. In contrast here, an extra important evolution occurs in the few hundred picosecond time range with an isosbestic point at 580 nm arising by the decay of the positive band at 535 nm and the increase of the positive band at 630 nm. The final transient spectrum in the nanosecond time delay are similar to the one of the closed form with a small contribution of triplet state at 460 nm. Nanosecond transient absorption experiments were then conducted degassed and aerated, and only a small decrease of few microsecond was observed at 460 nm dependent of oxygen (Fig. S20), *i.e.* cyclization quantum yield is independent of oxygen. Altogether, the last hundreds of picosecond time evolution is interpreted as the cyclization of the DTE units arising from an evolution of the above-mentioned triplet excited state as already proposed for transition metal complexes.^37,38^ All kinetic traces were then fitted by a global analysis with four and five components for 3Yb_ooo_ and 3Eu_ooo_, respectively, and a constant. Four and five decay-associated spectra (DAS) thus yielded (Fig. S20) together with the corresponding time constants of 0.84 ps, 5.10 ps, 67.0 ps and 998 ps for 3Yb_ooo_ and 0.14 ps, 0.94 ps, 5.95 ps, 52.5 ps and 188 ps for 3Eu_ooo_. The first and last DAS shows negative bands with a maximum at about 630 nm and with same amplitude. As *ϕ*_o/c_^1^ is in the range 0.9–1, the closed form is thus formed with equal probability from first excited singlet state (*ca.* 50%, first DAS) and from a triplet state (*ca.*50%, last DAS) that cyclizes efficiently. The shorter cyclization characteristic time for 3Eu_ooo_ (188 ps *vs.* 998 ps) might be rationalized with a faster transfer from the triplet state toward the f–f excited state in the case of europium that is closer in energy to the triplet state of DTE.^39^ Note that the deactivation of triplet state below 1 ns avoid the influence of oxygen and rationalizes the high cyclisation quantum yield in aerated solution.

Overall, it is very important to mention here that upon UV excitation, photo-isomerization and photo-luminescence are competing processes. In the case of the higher photocyclization quantum yields, knowing that a ±10% precision should be taken into consideration, it means that only a few percent of the absorbed photons can eventually lead to sensitization and then luminescence. However, the corresponding complexes do emit light under 350 nm excitation that is further quenched as the following section demonstrates.

### Emission studies

**Fig. 4:**
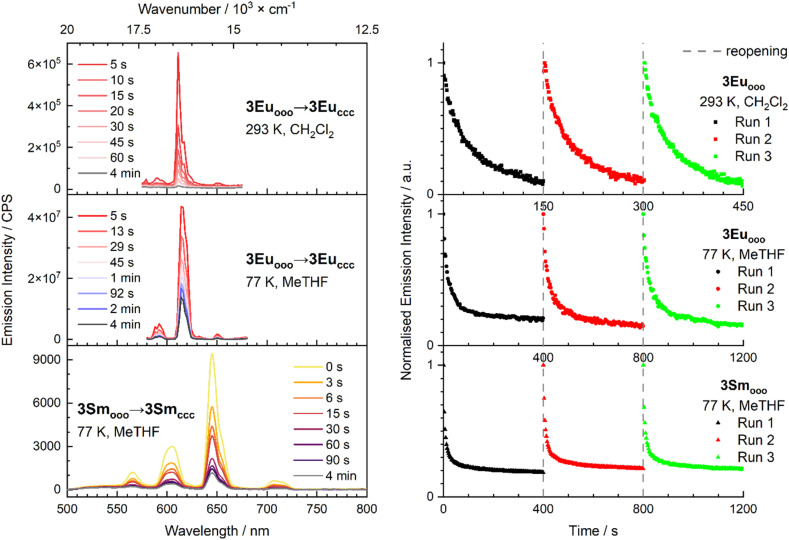
Emission spectra of 3Eu_ooo_ in CH_2_Cl_2_ at 293 K (top left) and 3Eu_ooo_ and 3Sm_ooo_ (bottom left) in MeTHF at 77 K. Emission decrease at 610 nm for 3Eu_ooo_ (top right) at 293 K and at 77 K, and at 645 nm for 3Sm_ooo_ at 77 K (bottom right) obtained upon continuous UV irradiation at *λ*_ex_ = 350 nm to PSS. The initial spectra were recovered after thawing the frozen solutions and 293 K irradiation at *λ*_ex_ = 660 nm between each decay (dashed grey lines).

The emission properties of all complexes, including 3Eu_ooo_ and 3Yb_ooo_ for consistency, were studied in aerated CH_2_Cl_2_ and MeTHF solutions at 293 K and 77 K. The complexes were generally excited at the absorption maxima of the open DTE units (*λ*_ex_ = 350 nm) to enable comparison with the previous results.^16^ It is worthwhile to highlight that with a conventional fluorimeter equipped with a scanning monochromator, the measurement of a distorted emission spectrum is likely as typical monochromator scan rates are comparable with the rate of the closing of the photochromic units upon continuous excitation leading to the loss of emission intensity upon scanning due to quenching by the closed DTE units (*vide infra*). As already pointed out, 3Eu_ooo_ shows very efficient closing reaction, meaning that emission quantum yields will be inherently weak while quenching occurs in a fast way. Nonetheless, in the case of 3Eu_ooo_ at 293 K, under excitation with small slit aperture, fast scan rate and short integration time, the typical Eu^3+^ emission could be detected (^5^D_0_ → ^7^F_J_*J* = 0–4), although with low resolution with a sharp transition located at *λ* = 611 nm (^5^D_0_ → ^7^F_2_ transition) and broader bands at *λ* = 590 nm (*J* = 0), 596 nm (*J* = 1), 651 nm (*J* = 3) for the ^5^D_0_ → ^7^F_3_ transition (Fig. 4, top left and S22). The high-resolution emission spectrum of 3Eu_ooo_ complex was obtained at 77 K in MeTHF organic glass, indicating the fine-splitting of *J* = 1, 2 and 4 transitions (Fig. S23). The efficiency of the emission quenching by the concomitant closure of DTE units upon irradiation is clearly evidenced by further loss of intensity to a value close to zero upon measuring successively several scans. Hence, to circumvent the inevitable closure of the DTE units during the first emission spectrum acquisition, we further used a CMOS-spectrometer-based detection for the kinetic experiments (see SI for details) that allows instantaneous spectrum recording over the whole visible spectral range and prevents distortion of the spectrum when measuring an evolving system. We first found out that 3Tb_ooo_ shows no luminescence neither at room nor at low temperature (Fig. S24). A similar result is obtained with 3Dy_ooo_ indicating that the open form of the diketonate ligand 1o is not a suitable antenna for these metal ions featuring higher energy excited state than europium(iii) (for terbium(iii): ^5^D_4_ = 20 500 cm^−1^, for dysprosium(iii): ^4^F_9/2_ = 21 100 cm^−1^ and for europium(iii): ^5^D_0_ = 17 400 cm^−1^).^40^ In contrast, 3Sm_ooo_, presenting a similar excited state energy as europium(iii) (for samarium(iii): ^4^G_5/2_ = 17 900 cm^−1^), can be sensitized by the coordinated antenna. Whereas extremely weak emission was observed at 293 K (Fig. S24), the four typical transitions (^4^G_5/2_ → ^6^H_J_ transition) could be observed at *λ* = 566 nm (*J* = 5/2), *λ* = 604 nm (*J* = 7/2), 645 nm (*J* = 9/2), 708 nm (*J* = 11/2) at 77 K (Fig. 4, bottom left). The weak emission of the Sm^3+^ complex at 293 K compared to Eu^3+^ is explained by the presence of additional emission in the NIR range, resulting in effective non-radiative relaxation.

For the two active visible emitters, the evolution of the emission spectral profile with time upon continuous irradiation at 350 nm is presented in Fig. 4 and is based on the most intense bands at 610 nm for 3Eu_ooo_ and at 645 nm for 3Sm_ooo_. Importantly, the DTE moieties are able to photoisomerize at 77 K ^41^  resulting in a quenching of the europium(iii) and samarium(iii) emission (Fig. 4 bottom) at that temperature. With CMOS detection at 293 K, less than 5% of the original europium(iii) emission intensity remained after 150 s irradiation, which is comparable to what was observed with the conventional PMT detector.^16^ At the same time, the temporal profiles obtained at 77 K showcase that 15% of Eu and Sm visible emission intensity remains even after 300 s of UV excitation (*λ*_ex_ = 350 nm) in these conditions leading to slower isomerisation, and that the process is reversible upon thawing and visible irradiation at *λ* = 660 nm.

**Fig. 5:**
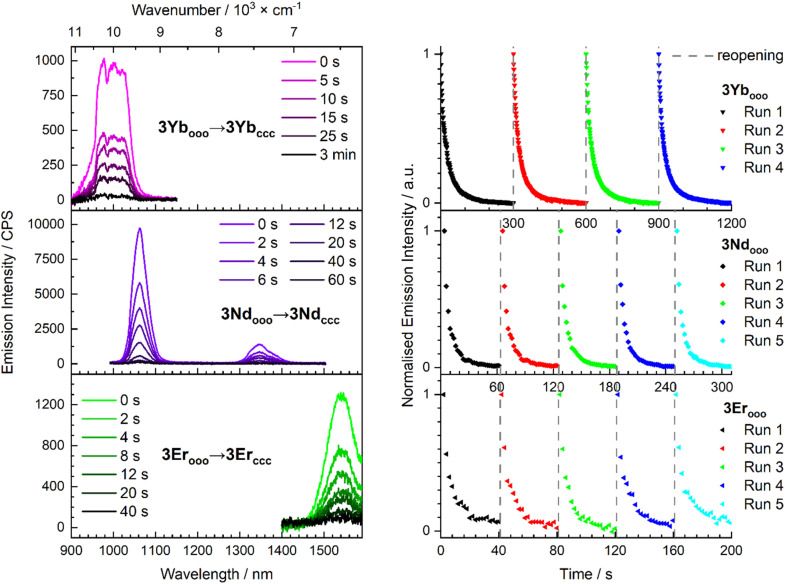
Emission spectra of 3Yb_ooo_, 3Nd_ooo_ and 3Er_ooo_ at 293 K (left), and emission decrease at 978 nm for 3Yb_ooo_, 1063 nm for 3Nd_ooo_ and 1540 nm for 3Er_ooo_ (right) in CH_2_Cl_2_ solutions obtained upon continuous UV irradiation at *λ*_ex_ = 350 nm to PSS. The initial spectra were recovered after 293 K irradiation at *λ*_ex_ = 660 nm between each decay (dashed grey lines).

In the case of the NIR emitters, we used a liquid N_2_ cooled NIR-InGAs CCD camera detection that also allows instantaneous detection over the whole spectral range (Fig. 5). 3Yb_ooo_ presents the characteristic of Yb^3+^ emission: a single ^2^F_5/2_ → ^2^F_7/2_ transition is detected at 970 nm (*λ*_ex_ = 350 nm) (Fig. 5). With 3Nd_ooo_, the characteristic line shape emission profile of the Nd^3+^ ion is observed at 1063 nm (^4^F_3/2_ → ^4^I_11/2_) and 1350 nm (^4^F_3/2_ → ^4^I_13/2_) in CH_2_Cl_2_ at 293 K and in an organic glass (MeTHF, 77 K) (Fig. S25–28). Finally, with 3Er_ooo_ the characteristic NIR emission at 1540 nm (^4^I_13/2_ → ^4^I_15/2_) was also observed in the same conditions at RT and 293 K and 77 K (Fig. S29 and 30). With those emitters, the decrease of luminescence is further monitored by successive acquisition of several spectra measured with the CCD camera detection upon continuous UV irradiation at *λ* = 350 nm. The efficiency of the emission quenching by the concomitant DTE closing upon irradiation is also clearly evidenced regardless of the excitation wavelength, as demonstrated for the 3Nd_ooo_ complex also excited at isosbestic point of photochemical conversion (*λ*_ex_ = 377 nm, Fig. S28). As for the visible emitters europium(iii) and samarium(iii), this experiment shows (i) the high reversibility and the stability of the photomodulation upon four or five successive open/close cycles with overlapping decay curves, and (ii) a quenching efficiency leading to only 2% of the initial intensity for Yb in 3 min, 1% for Nd in 1 min and 5% for Er in 40 s (Fig. 5). Note that no emission was detected when the complexes were excited at *λ*_ex_ = 660 nm indicating that the sensitization from the closed form unit is not possible.

**Fig. 6:**
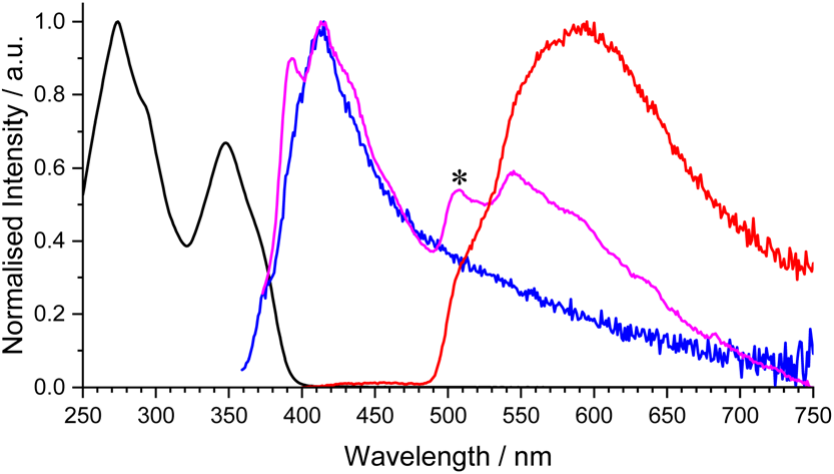
Normalised absorption (black, CH_2_Cl_2_) and emission spectra at 293 K (blue, CH_2_Cl_2_, *λ*_ex_ = 335 nm), at 77 K (magenta, MeTHF, *λ*_ex_ = 350 nm), and after 0.05 ms delay (red, MeTHF, *λ*_ex_ = 350 nm) of 3Gd_ooo_. The asterisk indicates the position of the lowest triplet excited state level (508 nm, 19 700 cm^−1^).

In all operating systems, these results highlight that the open DTE-β-diketonate and the phenanthroline ligands are responsible for the lanthanide luminescence sensitization, whereas the closed DTE form induces the quenching. In order to get deeper insight in this mechanism, 3Gd_ooo_ and 3Gd_ccc_ complexes were studied to further establish the energy diagram of the coordinated DTE-β-diketonate ligands (singlet and triplet states) (Fig. 6). The energies of the 3Gd_ooo_ and 3Gd_ccc_ singlet states can be estimated from the ligand centered emission by the *λ*_cut-off_ method to 390 nm (25 600 cm^−1^) and 750 nm (13 300 cm^−1^), respectively (293 K, CH_2_Cl_2_). On the other hand, the energy of the triplet state of 3Gd_ooo_ could be determined, using time-gated luminescence spectra at 77 K in a MeTHF organic glass, from the triplet emission maximum at 508 nm (19 700 cm^−1^), below that of the phenanthroline ligand measured in related lanthanide complexes.^44,45^ Unfortunately, we did not succeed to identify any triplet state in the case of 3Gd_ccc_ under similar conditions.

**Fig. 7:**
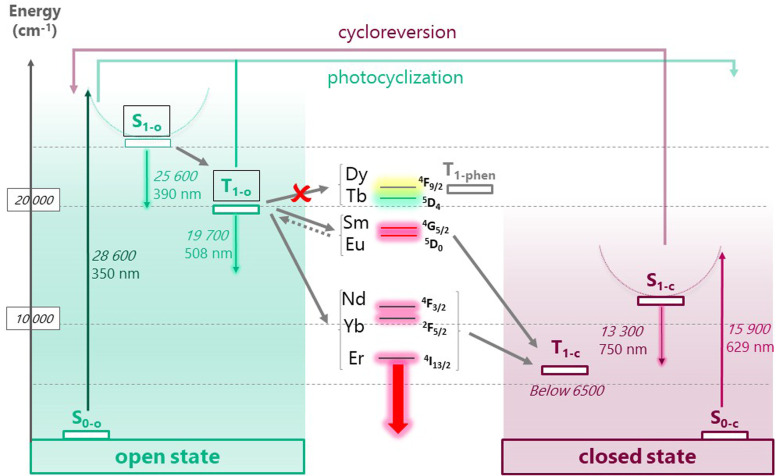
Jablonski diagram summarizing the photophysical and photochemical behavior of the different complexes in their open state (3Ln_ooo_, left) and closed state (3Ln_ccc_, right). The levels are positioned according to our observations for Ln = Gd, except for T_1-c_ (calculated position was used instead, *vide infra*). The phenanthroline triplet state (*ca.* 22 300 cm^−1^) corresponds to previously published data with Gd complexes featuring β-diketonate ligands.^44,45^ Relevant metal-centered emissive states are positioned at the center (see text for the exact values). Grey arrows represent possible energy transfers, including in the case of Ln = Eu, Sm, a thermally activated back energy transfer (dotted arrow).

By using the resulting Jablonski energy diagram (Fig. 7), rationalization of the sensitization process in the case of 3Ln_ooo_ complexes (Ln = Nd, Sm, Eu, Gd, Tb, Dy, Er, Yb) is now straightforward, with simple comparison of the relative positions of the lanthanide accepting levels and the DTE ligand centered singlet and triplet states. For Tb(iii) and Dy(iii), the open DTE ligand triplet state lies below the lanthanide excited state precluding any antenna effect. In contrast, in the case of all other lanthanide emitters, the position of the ligand triplet state of the open form is higher than the lanthanide accepting level, enabling the ligand mediated sensitization process. Interestingly, for europium(iii) and samarium(iii) the energy gap with the triplet are 2300 cm^−1^ and 1800 cm^−1^, respectively. This proximity makes additional thermally-activated back-energy transfer possible,^42,43^ explaining with the competitive DTE cyclisation pathway, the relatively modest europium(iii) and very weak samarium(iii) emission at 293 K. Concerning the closed form 3Ln_ccc_ (and related 3Ln_ooc_, and 3Ln_occ_), the singlet and *a* fortiori related triplet state are located below europium(iii) and samarium(iii) accepting levels, unambiguously precluding any sensitization.

Interestingly, in the case of the NIR emitting complexes (neodymium(iii): ^4^F_3/2_ = 11 600 cm^−1^ , ytterbium(iii): ^2^F_5/2_ = 10 300 cm^−1^, and erbium(iii): ^4^I_13/2_ = 6600 cm^−1^),^40^ displaying lanthanide emissive levels lying below the energy level of the closed DTE singlet (13 300 cm^−1^, *vide supra*), no emission is observed upon exciting at the absorption maxima of the isomerized DTE units (*λ*_ex_ = 660 nm). Furthermore, this latter state energy cannot explain the experimentally observed quenching of the NIR emitters. In addition, while time resolved luminescence measurements performed using a nanosecond 976 nm excitation allowed 3Yb_ooo_ emission plotting in function of time by signal integration between 940–990 nm (Fig. S21), after UV irradiation to 3Yb_ccc_ (PSS), no emission signal could be observed. Therefore, further investigation concerning a possible closed form triplet state lying below the NIR emissive ion excited state was necessary, as described in the following theoretical chemistry section.

### Theoretical calculations

**Fig. 8:**
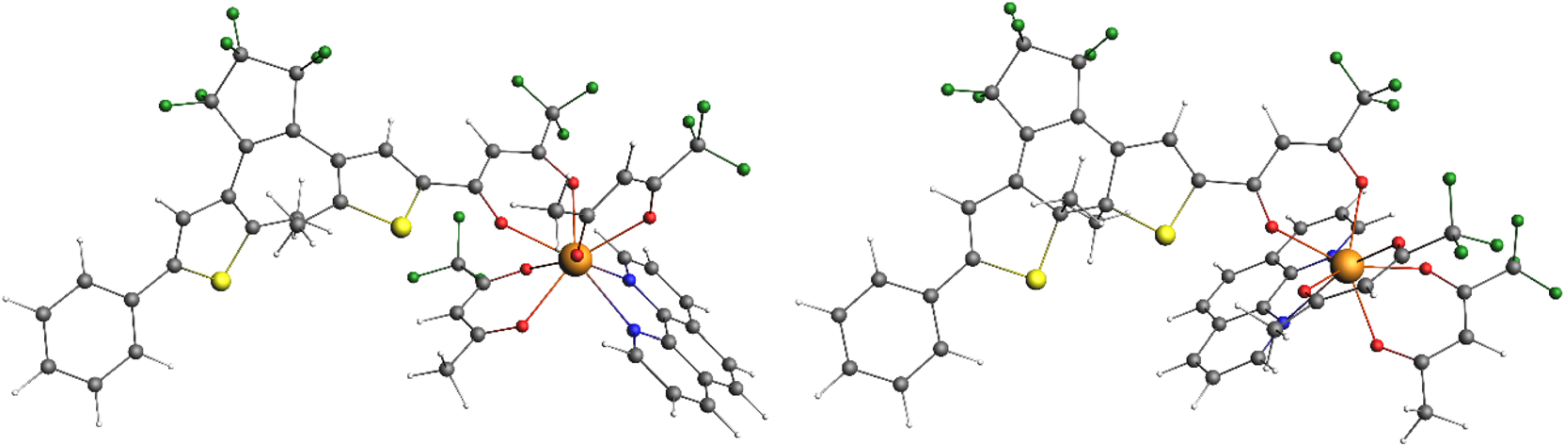
Representation of the DFT-optimized 3Yb_o_-S_0_ (left) and 3Yb_c_-S_0_ (right) model compounds.

To give more insights into the observed spectroscopic properties of the NIR emitters, calculations were performed on the Yb(iii) complexes for which experimental structural data is available, *i.e.*3Yb_ccc_. In order to reduce the computational cost, the experimental complexes were simplified with a model compound, containing only one DTE fragment, while the other two were replaced by methyl moieties. For both the open and closed forms, partial structural optimizations were performed at the DFT level to relax the atomic positions of the DTE fragment, while the rest of the molecules were kept at the same position in order to freeze the coordination sphere around the Yb(iii) centers (see computational details in SI). These optimizations led to the ground-state model structures named 3Yb_c_-S_0_ and 3Yb_o_-S_0_ in its antiparallel conformation (Fig. 8). For comparison purpose, a parallel conformation, *i.e.*3Yb_o//_-S_0_, was also investigated and shows similar behavior (Fig. S32 and Table S4). Structures of the lowest excited singlet state S_1_, and of the lowest triplet state T_1_, were both obtained at the TD-DFT level. The corresponding optimized structures 3Yb_c_-S_1_/T_1_, 3Yb_o_-S_1_/T_1_ and 3Yb_o//_-S_1_/T_1_ are shown in Fig. S33. The diabatic (vertical) and adiabatic (relaxed) energies of these excited states are given in Table S4. First, considering the vertical energies, the lowest excited state corresponds to a triplet spin state T_1_ lying above the ground state (S_0_) at *ca.* 1.1 and 2.7 eV in the closed and in both open models, respectively. In all models, the excited singlet state S_1_ is calculated about 1 eV higher in energy. Structural relaxations do not change the ordering between these excited states. However, in the closed form, a strong energetic stabilization of T_1_ over S_1_ is calculated.

In the following, successive levels of calculations are used to investigate these three models. First, TD-DFT calculation at the ground state S_0_ geometries are performed to estimate vertical transitions. Then, multi-reference WFT calculations are performed at the RASSCF level providing spin free (SF) and then spin orbit coupled states, and more accurate positioning of the singlet and triplet states of the ligand. Finally, the same calculations are performed at the first excited state S_1_ geometry, providing a very accurate picture of the systems. Note that in the discussion, the non-emissive closed isomer is systematically described before the emissive open system.

The energy state diagram at the TD-DFT level with the ground state S_0_ structures of the model compounds is shown in Fig. S34. As mentioned above, the S_1_ state in 3Yb_c_ is calculated at 14 822 cm^−1^ (1.8 eV), in line with the experimental value of 15 900 cm^−1^. As shown in Fig. S35, this S_0_ → S_1_ transition corresponds to a π–π* excitation centered on the DTE core, with typical antibonding character prefiguring ring opening. In both open forms, the S_1_ state is calculated at *ca.* 29 000 cm^−1^ (Fig. S34), again in agreement with the experimental value (28 600 cm^−1^). Interestingly, the oscillator strength associated to this first excitation is relatively low (*ca.* 0.03, see Table S5) , a larger oscillator strength (*ca.* 0.44) is calculated for the S_2_ states lying in the same energy range as S_1_. This excited state S_2_ corresponds to intra-DTE electronic excitations with some charge transfer character towards the β-diketonate (Fig. S35). The T_1_ state is calculated much lower in energy at 21 973 cm^−1^ (3Yb_o_) and 21 617 cm^−1^ (3Yb_o//_), in line with was is found experimentally (19 700 cm^−1^).

**Fig. 9:**
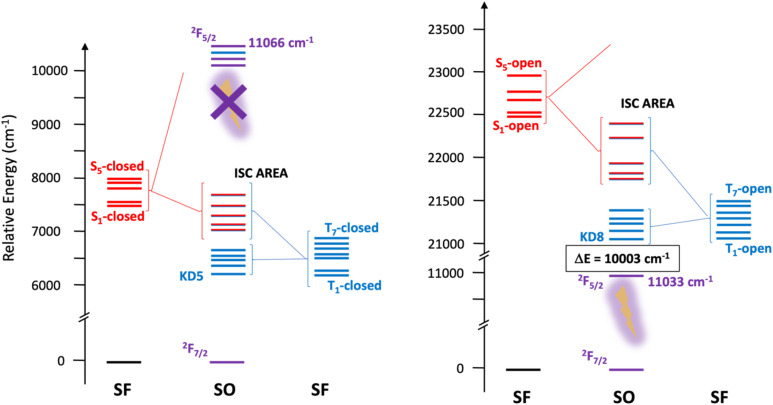
Calculated energy state diagrams (in cm^−1^) of 3Yb_c_-S_1_ (left) and 3Yb_o_-S_1_ (right) at the RASSCF level (see text for details). Note the change in the energy scale between the two state diagrams. The state diagram of 3Yb_o//_-S_1_ is shown in Fig. S39.

However, as TD-DFT tends to overestimate such energy gap, multi-reference WFT calculations were subsequently performed (see computational details in SI). The calculated energy state diagrams at the RASSCF level for the closed and open model structures in their S_1_ and S_0_ geometries are shown in Fig. 9 and S37, respectively. The numerical values of these diagrams are collected in Tables S6–S10 while the corresponding natural orbitals are given in Fig. S40–S45. Let's first naturally focus on the results obtained with the S_0_ geometries. At the spin-free (SF) level (*i.e.* without including spin–orbit coupling), the excited S_1_ states are calculated at 16 435 and 29 514 cm^−1^ in 3Yb_c_-S_0_ and 3Yb_o_-S_0_, respectively. Moreover, in all model compounds, the SF triplet spin states are located lower in energy than the S_1_ states (14 525 cm^−1^ and 27 380 cm^−1^ above the ground state for 3Yb_c_-S_0_ and 3Yb_o_-S_0_, respectively). The resulting small calculated Δ*E*_(S_1_–T_1_)_ energy gaps at the RASSCF level are in favor of a strong inter-system crossing between these states. The introduction of the spin–orbit (SO) coupling leads to a large admixture of singlet and triplet spin states for the non-metal centered Kramers doublet (KD) states (KD13–KD17, labeled as the ISC area in the energy diagrams, Fig. S37). These ISC areas are characterized by large transition matrix elements of the SO coupling operator, such as 〈S_1_|H^SO^|T_2_〉 = 3003 cm^−1^ in 3Yb_c_-S_0_, and 〈S4|H^SO^|T_3_〉 = 2885 cm^−1^ in 3Yb_o_-S_0_ (Table S12). Interestingly, a set of five KD states arising mainly from the SF triplet spin states are calculated below the ISC area, and can thus participate to non-radiative relaxation processes. In both 3Yb_c_-S_0_ and 3Yb_o_-S_0_ model structures, these KDs are located above the emissive Yb(iii) states (Table S6). Overall, these calculated energy state diagrams, based on the S_0_ geometries (Fig. S37) suggest that emission from the ytterbium(iii) states could be observable in both closed and open forms, which is in opposition to the experimental observations. Additionally, the large calculated energy gap (Δ*E* = 16 404 cm^−1^) between the emissive ^2^F_5/2_ states and the lowest KD states centered on the DTE moiety does not favor an efficient energy transfer between the DTE- and 4f-centered states.

To question the structural impact on the calculations, let's thus now concentrate on the calculated state energy diagrams obtained with the S_1_ geometries (Fig. 9). Interestingly, both at the SF and SO levels, a large energetic stabilization of the different calculated states is observed compared to those calculate with the S_0_ geometries, excepted for the ytterbium(iii) centered states that, as expected, remain at the same energies. The most dramatic changes are observed for 3Yb_c_-S_1_, where, at the SF level, S_1_ and T_1_ states are now calculated at 7513 and 6168 cm^−1^, respectively. Consequently, the spin–orbit KD states arising from the SF triplet states are now calculated at *ca.* 6500 cm^−1^ above the GS, and the ISC area is calculated at *ca.* 7500 cm^−1^ above the GS, well below the ytterbium(iii) centered ^2^F_5/2_ states. Such an energy state diagram is therefore in full agreement with the experimental picture and the absence of Yb(iii) luminescence in the closed forms. In 3Yb_o_-S_1_, a large stabilization of the different states is calculated when compared to those obtained with the S_0_ geometry. The S_1_ and T_1_ states are now calculated at 22 481 and 20 990 cm^−1^ above the GS, in close agreement with experiment (25 600 and 19 700 cm^−1^, respectively). The induced smaller energy gap favors overall a stronger mixing among these states when the SO coupling is introduced (Table S12). The energy gap between the emissive ^2^F_5/2_ states and the lowest KD states centered on the DTE unit is reduced to 10 003 cm^−1^. This is in line with both a better energy transfer toward the ytterbium(iii) states and the operant sensitization in the open state. For the sake of completion, the influence of dynamic correlation on the energy of the singlet and triplet spin states was investigated with the help of multi-configurational pair-density functional theory (MC-pDFT, Table S11), without modifying this overall picture.

## Discussion

In the light of the experimental and theoretical results, we can now provide a complete and consistent picture of the sensitization and quenching processes of the 3Ln_ooo_ series in agreement with each ion. First, the photoisomerization of all systems is fast and corresponds to high or very high first cyclisation quantum yields (*ϕ*_o/c_^1^ ranges from 0.43 to 0.99 ± 10%), whereas the second and third isomerization steps are less efficient. This feature provides our system with a desirable very fast response. However, the sensitization efficacy, *i.e.* the fraction of photon absorbed by the DTE units leading to population of the lanthanide excited state, is then limited by this concomitant isomerization process. This is qualitatively apparent when comparing with the naked eye similar solutions of [Eu(tta)(phen)] and 3Eu_ooo_ in DCM at the very beginning of the isomerization process. Unfortunately, experimental evaluation of luminescence quantum yields of 3Eu_ooo_ is precluded by the rapidity of this isomerization. This process is likely to occur from the singlet state of the excited ligand (AP form, 25 600 cm^−1^), at very short time scale (*vide supra*), and from the triplet state of the P form at longer time scale (*vide supra*). Therefore, the DTE triplet states (open and closed) investigation was mandatory since they are determinant in the sensitization of the lanthanide ion luminescence and its further photomodulation in the present case.

Both experimental and theoretical findings account here for an open ligand triplet state located at 19 700 cm^−1^, and thus unsuited for dysprosium and terbium sensitization as observed. This level is also quite low in comparison with europium and samarium emissive level, similar to the simpler 2-thenoyl-trifluoroacetonate with a triplet situated at 19 200 cm^−1^ leading to the same results in term of sensitization.^46^ Concerning the DTE closed state, we can conclude that a dark triplet state, calculated at a very low energy (6200 cm^−1^), acts as a ubiquitous quencher, whatever the lanthanide ion used, including for the very low lying erbium emitter. Similar low energy triplet state levels were theoretically calculated for diarylethene incorporating thiazole rings.^47^ Note that such a level is consistent with the absence of any sensitization of the NIR emitters in the closed state as observed.

From all these results, it arises that the objective of a versatile photochromic ligand efficient in the whole range of emitters from dysprosium to erbium is not out of reach, if one is able to design ligands with energy levels higher by a few thousands of wavenumbers in their open state. We have to mention here that photoswitching of terbium emission has already been reported in a few DTE containing systems with similar open state energy levels.^48–50^ However, the common feature of these systems is their supramolecular nature, and the fact that the distance between the DTE units and the terbium sites is larger, and less controlled, than in our strictly molecular system 3Tb_ooo_. This feature may allow sensitization by non-photochromic ligands in the ON state, the quenching by low lying DTE energy levels being less efficient at longer distances in the open state.^48,49^ On the opposite, this feature can also be detrimental to the trapping of energy in the OFF state leading to very low contrast (*i.e.* I_OFF_/I_ON_ ∼0.90).^50^ Therefore, and more generally, (i) the understanding of a given system has to consider intermolecular energy transfer, and (ii) strictly well-defined molecular systems are needed for a better understanding of the Tb^3+^ emission switching processes.

Finally, regarding the system emission and quenching efficiencies, this study importantly highlights the fact that if the isomerization efficiency affects the lanthanide ion emission, in turn and concomitantly, the lanthanide ion also influences the cyclisation efficiency through the DTE triplet state promotion in the open state with a dependence on the lanthanide ion that remains to be elucidated. Therefore, improving the energy transfer toward the emissive lanthanide excited states by decreasing the cyclisation efficiency of the photochromic unit(s), with a careful engineering is necessary to better balance those two competitive key features and for a better understanding of all the system features.

## Conclusion

In this article, we report that a stable system combining three DTE units and a lanthanide ion provides luminescence of various lanthanide ions associated with a concomitant emission quenching process under UV light owing to a very high efficiency of the DTE isomerisation process toward the closed form, either in its singlet or through its triplet excited states ascribed to spin orbit coupling with the lanthanide ion. The process is applicable to visible emitters (europium(iii), samarium(iii)) and more importantly for different NIR emitters (ytterbium(iii), neodymium(iii), erbium(iii)) and can be reversed under visible light irradiation in a highly repeatable way. Importantly, this unprecedented panel of Ln(iii) ions featuring this effect includes ions for which no previous examples are known such as samarium(iii), neodymium(iii), erbium(iii).

The quenching mechanisms with excellent efficiencies were investigated for the first time with experimental and advanced theoretical tools. They reveal (i) an unexpected large contribution of the open DTE triplet state in the closing mechanism, (ii) a surprisingly large dependence of the DTE closing efficiency on the lanthanide ion nature that remains to be elucidated, and (iii) the existence of a dark triplet state located on closed DTE units lying below the lanthanide ion excited state that explains the quenching of the NIR emitters. This level can also explain the visible emitters quenching whereas, usually, such closed DTE units are believe to act as Förster acceptor. Importantly, this study also highlights the importance of the balance between the emission and the quenching process efficiencies to obtain bright and effective systems.

Finally, these results provide new insight to develop new universal architectures able to apply this process to lanthanide ions (i) with higher energy emissive states, such as terbium(iii) and dysprosium(iii), not sensitized with this current system, and (ii) with overall higher initial emission efficiencies.

## Author contributions

Conceptualizations: L. N. and S. R.; synthesis T. A. P.; H. A.; photokinetic experiments: O. G., R. M.; Femto and nanosecond photophysical measurements C. M., A. B., M. S.; emissions studies, S. K., T. A. P., Y. F., F. R., B. M., A. B., O. M; DRX: M. D.; theoretical investigations: F. G. B.L.G. Supervision: O. M.; L. N.; S. R.; the manuscript was written through the contributions of all authors. All authors have approved the final version of the manuscript.

## Conflicts of interest

The authors declare no conflicts of interest.

## Supplementary Material

SC-OLF-D5SC07174G-s001

SC-OLF-D5SC07174G-s002

## Data Availability

CCDC 2402724 contains the supplementary crystallographic data for this paper.^51^ All experimental procedures, analytical data and spectra are available in the supplementary information (SI). Supplementary information is available. See DOI: https://doi.org/10.1039/d5sc07174g.

## References

[cit1] Binnemans K. , in Handbook on the Physics and Chemistry of Rare Earths, ed. K. A. Gschneidner, J.-C. G. Bünzli and V. K. Pecharsky, Elsevier, 2005, vol. 35, pp. 107–272

[cit2] Wang K.-Z. , in The Rare Earth Elements: Fundamentals and Applications, ed. D. A. Atwood, Wiley, 2012, pp. 249–262

[cit3] Aromí G., Gamez P., Reedijk J. (2008). Coord. Chem. Rev..

[cit4] Shao G., Yu H., Zhang N., He Y., Feng K., Yang X., Cao R., Gong M. (2014). Phys. Chem. Chem. Phys..

[cit5] Clegg J. K., Li F., Lindoy L. F. (2022). Coord. Chem. Rev..

[cit6] Dalal A., Nehra K., Hooda A., Singh D., Kumar P., Kumar S., Malik R. S., Rathi B. (2023). Inorg. Chim. Acta.

[cit7] Gálico D. A., Marin R., Brunet G., Errulat D., Hemmer E., Sigoli F. A., Moilanen J. O., Murugesu M. (2019). Chem. Eur. J..

[cit8] Lenaerts P., Driesen K., Van Deun R., Binnemans K. (2005). Chem. Mater..

[cit9] Puntus L. N., Schenk K. J., Bünzli J. C. G. (2005). Eur. J. Inorg. Chem..

[cit10] Norel L., Galangau O., Al Sabea H., Rigaut S. (2021). ChemPhotoChem.

[cit11] Di Piazza E., Norel L., Costuas K., Bourdolle A., Maury O., Rigaut S. (2011). J. Am. Chem. Soc..

[cit12] Norel L., Di Piazza E., Feng M., Vacher A., He X. Y., Roisnel T., Maury O., Rigaut S. (2014). Organometallics.

[cit13] Al Sabea H., Norel L., Galangau O., Hijazi H., Metivier R., Roisnel T., Maury O., Bucher C., Riobe F., Rigaut S. (2019). J. Am. Chem. Soc..

[cit14] Meshkova S. B., Topilova Z. M., Bolshoy D. V., Beltyukova S. V., Tsvirko M., Venchikov V. Y. (1999). Acta Phys. Pol., A.

[cit15] Binnemans K. , Rare-earth beta-diketonates, in, Handbook on the Physics and Chemistry of Rare Earths, 2005, pp. 107–272, 10.1016/s0168-1273(05)35003-3

[cit16] Al Sabea H., Norel L., Galangau O., Roisnel T., Maury O., Riobé F., Rigaut S. (2020). Adv. Funct. Mater..

[cit17] Fréroux Y. , Caussin L., El Beyrouti N., Rigaut S. and Norel L., in Handbook on the Physics and Chemistry of Rare Earths, ed. J.-C. G. Bünzli and S. M. Kauzlarich, Elsevier, 2024, vol. 65, pp. 35–91

[cit18] Hasegawa Y., Nakagawa T., Kawai T. (2010). Coord. Chem. Rev..

[cit19] Liao P.-Y., Liu Y., Ruan Z.-Y., Wang H.-L., Shi C.-G., Deng W., Wu S.-G., Jia J.-H., Tong M.-L. (2023). Inorg. Chem..

[cit20] Lan J.-F., Li J., Zhu J.-L., Yan G.-P., Ke H., Liao J.-Z. (2021). Inorg. Chem..

[cit21] Addanki S., Amiri I. S., Yupapin P. (2018). Results Phys..

[cit22] Wang T., Wang S., Liu Z., He Z., Yu P., Zhao M., Zhang H., Lu L., Wang Z., Wang Z., Zhang W., Fan Y., Sun C., Zhao D., Liu W., Bünzli J.-C. G., Zhang F. (2021). Nat. Mater..

[cit23] Kreidt E. , Kruck C. and Seitz M., in Handbook on the Physics and Chemistry of Rare Earths, ed. J.-C. G. Bünzli and V. K. Pecharsky, Elsevier, 2018, vol. 53, pp. 35–79

[cit24] Bünzli J.-C. G. (2015). Coord. Chem. Rev..

[cit25] Adewuyi J. A., Ung G. (2024). J. Am. Chem. Soc..

[cit26] Fortman J. J., Sievers R. E. (1971). Coord. Chem. Rev..

[cit27] Nielsen L. G., Sørensen T. J. (2020). Inorg. Chem..

[cit28] Armaroli N., De Cola L., Balzani V., Sauvage J.-P., Dietrich-Buchecker C. O., Kern J.-M. (1992). J. Chem. Soc., Faraday Trans..

[cit29] Feng J., Yu J.-B., Song S.-Y., Sun L.-N., Fan W.-Q., Guo X.-M., Dang S., Zhang H.-J. (2009). Dalton Trans..

[cit30] Nakatani K. , Piard J., Yu P., Métivier R., Photochromic materials: preparation, properties and applications, ed. H. Tian, J. Zhang, Wiley-VCH, 2016, pp. 1–45

[cit31] Fihey A., Perrier A., Browne W. R., Jacquemin D. (2015). Chem. Soc. Rev..

[cit32] Ishibashi Y., Fujiwara M., Umesato T., Saito H., Kobatake S., Irie M., Miyasaka H. (2011). J. Phys. Chem. C.

[cit33] Sotome H., Une K., Nagasaka T., Kobatake S., Irie M., Miyasaka H. (2020). J. Chem. Phys..

[cit34] Jean-Ruel H., Gao M., Kochman M. A., Lu C., Liu L. C., Cooney R. R., Morrison C. A., Miller R. J. D. (2013). J. Phys. Chem. B.

[cit35] Ishibashi Y., Umesato T., Kobatake S., Irie M., Miyasaka H. (2012). J. Phys. Chem. C.

[cit36] Fredrich S., Morack T., Sliwa M., Hecht S. (2020). Chemistry.

[cit37] Indelli M. T., Carli S., Ghirotti M., Chiorboli C., Ravaglia M., Garavelli M., Scandola F. (2008). J. Am. Chem. Soc..

[cit38] Jukes R. T. F., Adamo V., Hartl F., Belser P., De Cola L. (2004). Inorg. Chem..

[cit39] Thor W., Kai H.-Y., Yeung Y.-H., Wu Y., Cheung T.-L., Tam L. K. B., Zhang Y., Charbonnière L. J., Tanner P. A., Wong K.-L. (2024). JACS Au.

[cit40] Carnall W. T., Goodman G. L., Rajnak K., Rana R. S. (1989). J. Chem. Phys..

[cit41] Al Sabea H., Hamon N., Galangau O., Norel L., Maury O., Riobé F., Tripier R., Rigaut S. (2020). Inorg. Chem. Front..

[cit42] Parker D., Fradgley J. D., Wong K.-L. (2021). Chem. Soc. Rev..

[cit43] Samuel A. P. S., Xu J., Raymond K. N. (2009). Inorg. Chem..

[cit44] Wong H.-Y., Lo W.-S., Chan W. T. K., Law G.-L. (2017). Inorg. Chem..

[cit45] Zhuravlev K. P., Tsaryuk V. I., Kudryashova V. A. (2018). J. Fluorine Chem..

[cit46] Abbas Z., Dasari S., Beltrán-Leiva M. J., Cantero-López P., Páez-Hernández D., Arratia-Pérez R., Butcher R. J., Patra A. K. (2019). New J. Chem..

[cit47] Zhang Z., He L., Feng J., Liu X., Zhou L., Zhang H. (2020). Inorg. Chem..

[cit48] Li Z., Chen H., Li B., Xie Y., Gong X., Liu X., Li H., Zhao Y. (2019). Adv. Sci..

[cit49] Zhou W.-L., Dai X.-Y., Lin W., Chen Y., Liu Y. (2023). Chem. Sci..

[cit50] Verma P., Singh A., Maji T. K. (2021). Chem. Sci..

[cit51] CCDC 2402724: Experimental Crystal Structure Determination, 2025, 10.5517/ccdc.csd.cc2ln77r

