# Supplementary material for: From erbium(iii) to samarium(iii): generalized photomodulation of NIR to red lanthanide luminescence with a DTE ligand and its versatile role in the quenching processes
Source: Chem Sci. 2025 Oct 27;16(47):22504–16. doi: 10.1039/d5sc07174g (PMC12555630; doi:10.1039/d5sc07174g)
Supplement: SC-016-D5SC07174G-s001 [file SC-016-D5SC07174G-s001.pdf]

Supplementary Information (SI) for Chemical Science.
This journal is © The Royal Society of Chemistry 2025

SUPPORTING INFORMATION

From Erbium(III) to Samarium(III): Generalized Photomodulation of NIR to Red Lanthanide Luminescence with a DTE Ligand and its Versatile Role in the Quenching Processes

Tuan-Anh Phan,[a] *Frédéric Gendron,*[a] *Salauat Kiraev,*[b] *Olivier Galangau,*[a] *Yoann Fréroux,*[a] *Hassan Al Sabea,*[a] *Cédric Mittelheisser,*[d] *Marie Dallon,*[a] *Aude Bouchet,*[d] *François Riobé,*[c] *Remi Métivier,*[f] *Michel Sliwa,*[d,e] *Boris Le Guennic,*[a] *Olivier Maury,*[b,*] *Akos Banyasz,*[b] *Bogdan Marekha,*[b] *Lucie Norel,*[a,*] *and Stéphane Rigaut*[a,*]

[a] Univ Rennes, CNRS, ISCR (Institut des Sciences Chimiques de Rennes) -UMR 6226, F-35000 Rennes, France E-mail: stephane.rigaut@univ-rennes1.fr, lucie.norel@univ-rennes1.fr
[b] ENS de Lyon, CNRS, LCH, UMR 5182, 69342, Lyon cedex 07, France E-mail: olivier.maury@ens-lyon.fr
[c] Univ. Bordeaux, CNRS, Bordeaux INP, ICMCB, UMR 5026, F-33600 Pessac, France
[d] Univ. Lille CNRS UMR 8516 LASIRE Laboratoire de Spectroscopie pour les Interactions, la Réactivité et l'Environnement, Lille, F59 000 France
[e] LOB, CNRS, INSERM, École Polytechnique, Institut Polytechnique de Paris, 91120 Palaiseau, France
[f] UMR CNRS 8531-PPSM, ENS Paris-Saclay, Université Paris-Saclay, 94235 Cachan, France.

TABLE OF CONTENTS

SYNTHETIC PROCEDURES 2

NMR, IR, UV-VIS AND MASS SPECTRA 7

UV-VIS STUDIES AND PHOTOCHROMIC REACTIONS 23

TRANSIENT ABSORPTION AND EMISSION SPECTROSCOPY 35

EMISSION SPECTROSCOPY STUDIES 37

CRYSTALLOGRAPHIC STUDIES 42

THEORITICAL CALCULATIONS 45

Synthetic procedures

General methods. All manipulations were performed in Schlenk-type flasks under dry nitrogen. Solvents were dried by conventional methods and distilled immediately prior to use. Deuterated solvents were passed down a 5 cm-thick alumina column and stored under nitrogen over molecular sieves (4 Å). Routine ^{1}H and ^{19}F{1H} spectra were recorded on a Bruker AVANCE III 400 MHz. The NMR signals were tentatively ascribed by comparison with similar compound (2D experiments were inconclusive). Mass spectra were recorded either on a Bruker MicroTOF spectrometer (ESI-TOF). Elemental analyses were performed by the CRMPO (Le Centre régional de mesures physiques de l'Ouest). All commercial reagents were used as supplied. Note that for some paramagnetic lanthanide complexes, it was not possible to assign all of the signals precisely. The DTE-β–diketone **1o** and the Europium/Ytterbium bis-aqua complexes have been obtained as previously reported.[1]

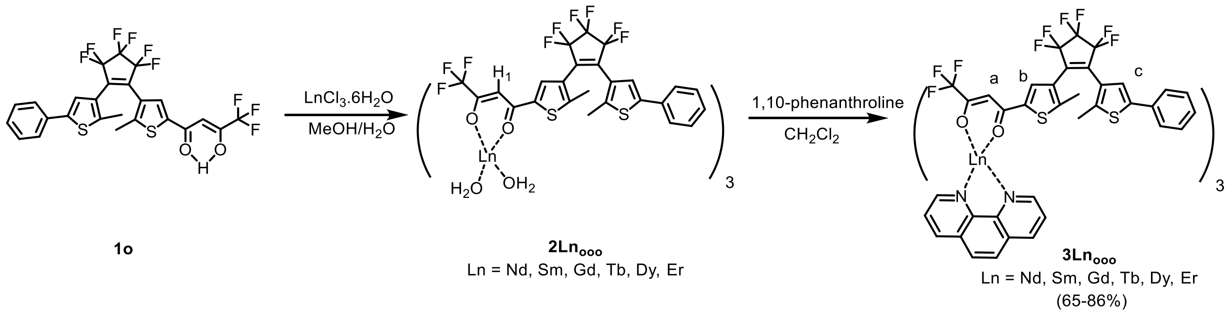

1o **2Ln$_{ooo}$** Ln = Nd, Sm, Gd, Tb, Dy, Er **3Ln$_{ooo}$** Ln = Nd, Sm, Gd, Tb, Dy, Er (65-86%)

General procedure A for the preparation of 2Ln$_{ooo}$

DTE-derived β-diketone (1 equiv.) was dissolved in a 1:1 CH_3OH/H_2O mixture, then the appropriate lanthanide salt (1 equiv.) was added while maintaining the pH between 6 and 6.5. The mixture was stirred for 0.5 h at room temperature, and water (50 mL) was added. The solution was stirred for another 10 min and the precipitate was filtered off. The latter was washed successively with water (10 mL) and pentane (0.5 mL) to afford the desired bis-aqua product.

General procedure B for the preparation of 3Ln$_{ooo}$

To a solution of **2Ln$_{ooo}$** (1 equiv.) in dichloromethane (5 mL) was added 1,10-phenanthroline (1 equiv.). The solution was stirred for 24 h at room temperature. The reaction mixture was then concentrated to ca 0.5 mL, and pentane (10 mL) was added. The precipitate was filtered off and dried under vacuum to afford the desired product.

2Nd$_{ooo}$. Prepared according to general procedure **A** from **1o** (40 mg, 0.069 mmol) and $NdCl_3.6H_2O$ (8.2 mg, 0.023 mmol) to give **2Nd$_{ooo}$** (35 mg, 79% yield) as a white bluish powder.

^{1}H NMR (400 MHz, CD_2Cl_2, 297 K): δ (ppm) = 7.44 (m, 9H, H_{ortho} and H_{para}), 7.27 (m, 12H, H_{meta}, H_b and H_c), 1.97 (s, 9H, CH_3), 1.43 (s, 4H, H_2O), (H_a resonance is not detected).

$^{19}F\{^1H\}$ NMR (376 MHz, CD_2Cl_2, 297 K): δ (ppm) = -72.40 (bs, 9F, CF_3), -110.29 (bs, 6F, CF_2), -110.53 (bs, 6F, CF_2), -132.13 (bs, 6F, CF_2).
IR (KBr): 3447 (s), 1612 (s), 1545 (m), 1508 (w), 1444 (m), 1338 (w), 1305 (s), 1274 (s), 1246 (w), 1194 (s), 1140 (s), 1057 (m), 988 (m), 896 (w), 789 (w), 756 (w), 689 (w), 575 (w), 538 (w).
Elem. Anal. Calcd for $C_{75}H_{46}O_8S_6F_{27}Nd$: C, 46.80; H, 2.41; S, 9.99. Found: C, 48.32; H, 2.62; S, 9.85.
HR-MS ESI (CH_3COCH_3): 2466.025 ([*NdL*$_4$]$^-$, calcd for $C_{100}H_{56}O_8S_8F_{36}Nd$: 2466.025).

2Sm$_{ooo}$. Prepared according to general procedure **A** from **1o** (50 mg, 0.086 mmol) and $SmCl_3.6H_2O$ (10.4 mg, 0.028 mmol) to give **2Sm$_{ooo}$** (51 mg, 92 % yield) as a white bluish powder.
^1H NMR (400 MHz, CD_2Cl_2, 297 K): δ (ppm) = 7.91 (bs, 3H, H_a or H_b), 7.50 (d, 6H, $^3J_{H,H}$ = 7.5 Hz, H_{ortho}), 7.34 (t, 6H, $^3J_{H,H}$ = 7.5 Hz, H_{meta}), 7.27 (t, 3H, $^3J_{H,H}$ = 7.5 Hz, H_{para}), 7.26 (s, 3H, H_c), 1.90 (s, 18H, CH_3), 1.47 (s, 4H, H_2O), (H_a or H_b resonance is not detected).
$^{19}F\{^1H\}$ NMR (376 MHz, CD_2Cl_2, 297 K): δ (ppm) = -75.50 (bs, 9F, CF_3), -110.39 (bs, 6F, CF_2), -110.54 (bs, 6F, CF_2), -132.20 (bs, 6F, CF_2).
IR (KBr): 3441 (s), 1611 (s), 1544 (m), 1505 (m), 1446 (m), 1338 (m), 1307 (s), 1274 (s), 1245 (m), 1193 (s), 1140 (s), 1056 (m), 986 (m), 899 (w), 787 (w), 757 (w), 690 (w), 579 (w), 537 (w).
Elem. Anal. Calcd for $C_{75}H_{46}O_8S_6F_{27}Sm$: C, 46.65; H, 2.40; S, 9.96. Found: C, 48.02; H, 2.65; S, 9.84.
HR-MS ESI (CH_3COCH_3): 2476.036 ([*SmL*$_4$]$^-$, calcd for $C_{100}H_{56}O_8S_8F_{36}Sm$: 2476.037).
.

2Gd$_{ooo}$. Prepared according to general procedure **A** from **1o** (50 mg, 0.086 mmol) and $GdCl_3.6H_2O$ (10.6 mg, 0.028 mmol) to give **2Gd$_{ooo}$** (48 mg, 87% yield) as a white bluish powder.
IR (KBr): 3442 (s), 1609 (s), 1545 (m), 1509 (m), 1446 (m), 1338 (m), 1309 (s), 1274 (s), 1247 (m), 1194 (s), 1140 (s), 1056 (m), 986 (m), 899 (w), 789 (w), 757 (w), 690 (w), 581 (w), 536 (w).
Elem. Anal. Calcd for $C_{75}H_{46}O_8S_6F_{27}Gd$: C, 46.49; H, 2.39; S, 9.93. Found: C, 47.01; H, 2.57; S, 9.61.
HR-MS ESI (CH_3COCH_3): 2482.041 ([*GdL*$_4$]$^-$, calcd for $C_{100}H_{56}O_8S_8F_{36}Gd$: 2482.041).

2Tb$_{ooo}$. Prepared according to general procedure **A** from **1o** (50 mg, 0.086 mmol) and $TbCl_3.6H_2O$ (10.7 mg, 0.028 mmol) to give **2Tb$_{ooo}$** (52 mg, 94% yield) as a white bluish powder.
IR (KBr): 3448 (s), 1608 (s), 1545 (m), 1508 (w), 1445 (m), 1338 (m), 1309 (s), 1274 (s), 1248 (m), 1194 (s), 1140 (s), 1056 (m), 987 (m), 899 (w), 789 (w), 757 (w), 690 (w), 582 (w), 537 (w).
Elem. Anal. Calcd for $C_{75}H_{46}O_8S_6F_{27}Tb$: C, 46.45; H, 2.39; S, 9.92. Found: C, 48.41; H, 2.97; S, 9.57.
HR-MS ESI (CH_3COCH_3): 2483.042 ([*TbL*$_4$]$^-$, calcd for $C_{100}H_{56}O_8S_8F_{36}Tb$: 2483.043).

2Dy$_{ooo}$. Prepared according to general procedure **A** from **1o** (50 mg, 0.086 mmol) and $DyCl_3.6H_2O$ (10.8 mg, 0.028 mmol) to give **2Dy$_{ooo}$** (50 mg, 90% yield) as a white bluish powder.
IR (KBr): 3443 (s), 1608 (s), 1546 (m), 1509 (w), 1446 (m), 1338 (m), 1310 (s), 1275 (s), 1248 (m), 1195 (s), 1141 (s), 1057 (m), 987 (m), 899 (w), 789 (w), 757 (w), 690 (w), 582 (w), 537 (w).
Elem. Anal. Calcd for $C_{75}H_{46}O_8S_6F_{27}Dy$: C, 46.36; H, 2.39; S, 9.90. Found: C, 48.74; H, 2.72; S, 9.93.
HR-MS ESI (CH_3COCH_3): 2488.047 ([*DyL*$_4$]$^-$, calcd for $C_{100}H_{56}O_8S_8F_{36}Dy$: 2488.046).

$2Er_{ooo}$. Prepared according to general procedure **A** from **1o** (40 mg, 0.069 mmol) and $ErCl_3.6H_2O$ (8.7 mg, 0.023 mmol) to give **$2Er_{ooo}$** (41 mg, 92% yield) as a white bluish powder.

^{1}H NMR (400 MHz, CD_2Cl_2, 297 K): δ (ppm) = 8.04 (bs, 3H, H_b), 7.83 (bs, 6H, H_{ortho}), 7.48 (m, 12H, H_{meta}, H_{para} and H_c), 3.61 (s, 9H, CH_3), 2.03 (s, 9H, CH_3), (H_a resonance is not detected).

^{19}F{^{1}H} NMR (376 MHz, CD_2Cl_2, 297 K): δ (ppm) = -100.50 (bs, 9F, CF_3), -110.69 (bs, 6F, CF_2), -110.98 (bs, 6F, CF_2), -132.52 (bs, 6F, CF_2).

IR (KBr): 3445 (s), 1613 (s), 1546 (m), 1508 (w), 1445 (m), 1338 (m), 1311 (s), 1274 (s), 1247 (m), 1195 (s), 1140 (s), 1057 (m), 987 (m), 899 (w), 789 (w), 756 (w), 690 (w), 566 (w), 537 (w).

Elem. Anal. Calcd for $C_{75}H_{46}O_8S_6F_{27}Er$: C, 46.25; H, 2.38; S, 9.88. Found: C, 48.33; H, 2.79; S, 9.73.

HR-MS ESI (CH_3COCH_3): 2490.047 ([*ErL4*]$^-$, calcd for $C_{100}H_{56}O_8S_8F_{36}Er$: 2490.047).

$3Nd_{ooo}$. Prepared according to general procedure **B** from **$2Nd_{ooo}$** (30 mg, 0.016 mmol) and 1,10-phenanthroline (2.8 mg, 0.016 mmol). Precipitation in CH_2Cl_2/pentane gave **$3Nd_{ooo}$** (21 mg, 65% yield) as a light-yellow powder.

^{1}H NMR (400 MHz, CD_2Cl_2, 297 K): δ (ppm) = 11.28 (s, 3H, H_b), 9.33 (s, 3H, H_a), 7.49 (d, 6H, $^3J_{H,H}$ = 7.5 Hz, H_{ortho}), 7.32 (bs, 3H, H_c), 7.31 (t, 6H, $^3J_{H,H}$ = 7.5 Hz, H_{meta}), 7.25 (dd, 3H, $^3J_{H,H}$ = 7.5 Hz, H_{para}), 6.98 (d, 2H, $^3J_{H,H}$ = 8.1 Hz, H_{phen}), 6.51 (s, 2H, H_{phen}), 6.29 (d, 2H, $^3J_{H,H}$ = 8.1 Hz, H_{phen}), 3.59 (bs, 2H, H_{phen}), 2.09 (s, 9H, CH_3), 1.54 (s, 9H, CH_3) ppm.

^{19}F{^{1}H} NMR (376 MHz, CD_2Cl_2, 297K): δ (ppm) = -73.46 (s, 9F, CF_3), -110.23 (bs, 6F, CF_2), -110.44 (bs, 6F, CF_2), -132.08 (q, 6F, $^3J_{F,F}$ = 5.0 Hz, CF_2) ppm.

IR (KBr): 1626 (m), 1603 (s), 1580 (m), 1543 (m), 1505 (m), 1446 (m), 1338 (m), 1307 (s), 1274 (s), 1243 (m), 1193 (s), 1140 (s), 1056 (m), 986 (m), 898 (w), 843 (w), 788 (w), 757 (w), 730 (w), 690 (w), 579 (w), 537 (w).

Elem. Anal. Calcd for $C_{87}H_{50}O_6N_2S_6F_{27}Nd.1C_5H_{12}$: C, 51.61; H, 2.92; N, 1.31; S, 8.98. Found: C, 52.31; H, 2.95; N, 1.65; S, 9.15.

HR-MS ESI (CH_2Cl_2): 2065.063 ([*M*]$^{\circ+}$, calcd for $C_{87}H_{50}O_6N_2S_6F_{27}Nd$: 2065.063).

UV-vis (CH_2Cl_2): λ_{max} (ε) = 274 (105200), 350 (64100) nm ($M^{-1}.cm^{-1}$).

$3Sm_{ooo}$. Prepared according to general procedure **B** from **$2Sm_{ooo}$** (40 mg, 0.021 mmol) and 1,10-phenanthroline (3.7 mg, 0.021 mmol). Precipitation in CH_2Cl_2/pentane gave **$3Sm_{ooo}$** (37 mg, 86% yield) as a light-yellow powder.

^{1}H NMR (400 MHz, CD_2Cl_2, 297 K): δ (ppm) = 8.82 (bs, 2H, H_{phen}), 8.21 (d, 2H, $^3J_{H,H}$ = 8.1 Hz, H_{phen}), 7.85 (s, 3H, H_a), 7.66 (s, 2H, H_{phen}), 7.59 (dd, 2H, $^3J_{H,H}$ = 8.1 Hz, $^3J_{H,H}$ = 4.4 Hz, H_{phen}), 7.51 (d, 6H, $^3J_{H,H}$ = 7.5 Hz, H_{ortho}), 7.35 (t, 6H, $^3J_{H,H}$ = 7.5 Hz, H_{meta}), 7.29 (t, 3H, $^3J_{H,H}$ = 7.5 Hz, H_{para}), 7.28 (s, 3H, H_b), 7.27 (s, 3H, H_c), 1.95 (s, 9H, CH_3), 1.88 (s, 9H, CH_3).

^{19}F{^{1}H} NMR (376 MHz, CD_2Cl_2, 297 K): δ (ppm) = -75.95 (s, 9F, CF_3), -110.40 (bs, 6F, CF_2), -110.53 (bs, 6F, CF_2), -132.17 (q, 6F, $^3J_{F,F}$ = 5.2 Hz, CF_2) ppm.

IR (KBr): 1628 (m), 1605 (s), 1580 (m), 1543 (m), 1506 (m), 1446 (m), 1338 (m), 1309 (s), 1274 (s), 1243 (m), 1193 (s), 1140 (s), 1057 (m), 987 (m), 899 (w), 842 (w), 788 (w), 758 (w), 730 (w), 690 (w), 579 (w), 537 (w).

Elem. Anal. Calcd for $C_{87}H_{50}O_6N_2S_6F_{27}Sm$: C, 50.36; H, 2.43; N, 1.35; S, 9.27. Found: C, 50.86; H, 2.42; N, 1.09; S, 8.88.

HR-MS ESI (CH_2Cl_2): 1494.043 ([M – (**1o**-H)]$^+$, calcd for $C_{62}H_{36}O_4N_2S_4F_{18}Sm$: 1494.062) and 2075.076 ([M]$^{°+}$, calcd for $C_{87}H_{50}O_6N_2S_6F_{27}Sm$: 2075.0756).

UV-vis (CH_2Cl_2): λ_{max} (ε) = 274 (100600), 349 (64700) nm ($M^{-1}.cm^{-1}$).

3Gd$_{ooo}$. Prepared according to general procedure **B** from **2Gd$_{ooo}$** (30 mg, 0.015 mmol) and 1,10-phenanthroline (2.8 mg, 0.015 mmol). Precipitation in CH_2Cl_2/pentane gave **3Gd$_{ooo}$** (25 mg, 78% yield) as a light-yellow powder.

IR (KBr): 1630 (m), 1606 (s), 1580 (m), 1544 (m), 1506 (m), 1446 (m), 1338 (m), 1311 (s), 1274 (s), 1243 (m), 1193 (s), 1140 (s), 1056 (m), 986 (m), 899 (w), 842 (w), 788 (w), 756 (w), 729 (w), 690 (w), 580 (w), 537 (w).

Elem. Anal. Calcd for $C_{87}H_{50}O_6N_2S_6F_{27}Gd$: C, 50.19; H, 2.42; N, 1.35; S, 9.24. Found: C, 50.31; H, 3.02; N, 1.60; S, 8.86.

HR-MS ESI (CH_3COCH_3): 2081.081 ([M]$^{°+}$, calcd for $C_{87}H_{50}O_6N_2S_6F_{27}Gd$: 2081.080).

UV-vis (CH_2Cl_2): λ_{max} (ε) = 274 (99400), 348 (61500) nm ($M^{-1}.cm^{-1}$).

3Tb$_{ooo}$. Prepared according to general procedure **B** from **2Tb$_{ooo}$** (40 mg, 0.021 mmol) and 1,10-phenanthroline (3.7 mg, 0.021 mmol). Precipitation in CH_2Cl_2/pentane gave **3Tb$_{ooo}$** (34 mg, 79% yield) as a light-yellow powder.

^{1}H NMR (400 MHz, CD_2Cl_2, 297 K): δ (ppm) = 114.35 (bs, 3H, H_a), 7.72 (s, 3H, H_c), 6.74 (bs, 6H, H_{ortho}), 6.38 (bs, 3H, H_{para}), 6.27 (bs, 9H, H_{meta}), 4.81 (s, 9H, CH_3), 1.28 (s, 9H, H_b), -0.38 (s, 9H, CH_3), -9.06 (bs, 2H, H_{phen}), -13.20 (bs, 2H, H_{phen}), -31.69 (s, 2H, H_{phen}) (resonances of the protons in the 2 and 9-positions of 1,10-phenanthroline are not detected).

^{19}F{^{1}H} NMR (376 MHz, CD_2Cl_2, 297 K): δ (ppm) = -51.64 (bs, 9F, CF_3), -107.62 (bs, 6F, CF_2), -109.59 (bs, 6F, CF_2), -130.87 (bs, 6F, CF_2).

IR (KBr): 1631 (m), 1604 (s), 1578 (m), 1544 (m), 1505 (m), 1446 (m), 1338 (m), 1310 (s), 1273 (s), 1242 (m), 1193 (s), 1141 (s), 1057 (m), 986 (m), 899 (w), 841 (w), 788 (w), 756 (w), 729 (w), 690 (w), 582 (w), 537 (w).

Elem. Anal. Calcd for $C_{87}H_{50}O_6N_2S_6F_{27}Tb.1C_5H_{12}$: C, 51.26; H, 2.90; N, 1.30; S, 8.92. Found: C, 51.80; H, 2.49; N, 1.49; S, 8.86.

HR-MS ESI (CH_2Cl_2): 2082.081 ([M]$^{°+}$, calcd for $C_{87}H_{50}O_6N_2S_6F_{27}Tb$: 2082.081).

UV-vis (CH_2Cl_2): λ_{max} (ε) = 274 (101400), 348 (65900) nm ($M^{-1}.cm^{-1}$).

$3Dy_{ooo}$. Prepared according to general procedure **B** from **$2Dy_{ooo}$** (40 mg, 0.021 mmol) and 1,10-phenanthroline (3.7 mg, 0.021 mmol). Precipitation in CH_2Cl_2/pentane gave **$3Dy_{ooo}$** (35 mg, 81% yield) as a light-yellow powder.

^1H NMR (400 MHz, CD_2Cl_2, 297 K): δ (ppm) = 136.87 (bs, 3H, H_a), 7.46 (s, 3H, H_c), 6.67 (bs, 6H, H_{ortho}), 6.28 (bs, 9H, H_{meta} and H_{para}), 5.56 (s, 9H, CH_3), 1.34 (s, 3H, H_b), -3.05 (s, 9H, CH_3), -42.87 (bs, 2H, H_{phen}), -47.55 (bs, 2H , H_{phen}), -66.42 (s, 2H, H_{phen}) (resonances of the protons in the 2 and 9-positions of 1,10-phenanthroline are not detected).

$^{19}F\{^1H\}$ NMR (376 MHz, CD_2Cl_2, 297 K): δ (ppm) = -40.27 (bs, 9F, CF_3), -108.84 (bs, 6F, CF_2), -109.99 (bs, 6F, CF_2), -131.49 (bs, 6F, CF_2).

IR (KBr): 1628 (m), 1605 (s), 1580 (m), 1543 (m), 1506 (m), 1446 (m), 1338 (m), 1309 (s), 1274 (s), 1243 (m), 1193 (s), 1140 (s), 1057 (m), 987 (m), 899 (w), 843 (w), 788 (w), 757 (w), 730 (w), 690 (w), 579 (w), 537 (w).

Elem. Anal. Calcd for $C_{87}H_{50}O_6N_2S_6F_{27}Dy$: C, 50.07; H, 2.41; N, 1.34; S, 9.22. Found: C, 51.15; H, 2.51; N, 1.09; S, 8.69.

HR-MS ESI (CH_2Cl_2): 2087.086 ($[M]^{\circ+}$, calcd for $C_{87}H_{50}O_6N_2S_6F_{27}Dy$: 2087.085).

UV-vis (CH_2Cl_2): λ_{max} (ε) = 273 (100500), 347 (66900) nm ($M^{-1}.cm^{-1}$).

$3Er_{ooo}$. Prepared according to general procedure **B** from **$2Er_{ooo}$** (30 mg, 0.015 mmol) and 1,10-phenanthroline (2.8 mg, 0.015 mmol). Precipitation in CH_2Cl_2/pentane gave **$3Er_{ooo}$** (26 mg, 81% yield) as a light-yellow powder.

^1H NMR (400 MHz, CD_2Cl_2, 297K): δ (ppm) = 17.85 (bs, 2H, H_{phen}), 17.30 (s, 2H, H_{phen}), 15.55 (bs, 2H, H_{phen}), 7.65 (bs, 15H, H_{ortho}, H_{meta} and H_b), 7.51 (s, 3H, H_{para}), 6.98 (s, 3H, H_c), 2.41 (s, 9H, CH_3), 1.22 (s, 9H, CH_3), -15.17 (s, 3H, H_a).

$^{19}F\{^1H\}$ NMR (376 MHz, CD_2Cl_2, 297K): δ (ppm) = -93.13 (bs, 9F, CF_3), -110.95 (bs, 6F, CF_2), -111.44 (bs, 6F, CF_2), -132.72 (bs, 6F, CF_2).

IR (KBr): 1634 (m), 1608 (s), 1579 (m), 1544 (m), 1506 (m), 1447 (m), 1338 (m), 1313 (s), 1273 (s), 1244 (m), 1193 (s), 1140 (s), 1057 (m), 987 (m), 899 (w), 842 (w), 787 (w), 757 (w), 730 (w), 690 (w), 582 (w), 537 (w).

Elem. Anal. Calcd for $C_{87}H_{50}O_6N_2S_6F_{27}Er$: C, 49.95; H, 2.41; N, 1.34; S, 9.20. Found: C, 50.96; H, 3.00; N, 1.57; S, 8.95.

HR-MS ESI (CH_2Cl_2): 2089.087 ($[M]^{\circ+}$, calcd for $C_{87}H_{50}O_6N_2S_6F_{27}Er$: 2089.086).

UV-vis (CH_2Cl_2): λ_{max} (ε) = 274 (99800), 346 (63700) nm ($M^{-1}.cm^{-1}$).

NMR, IR, UV-Vis and mass spectra

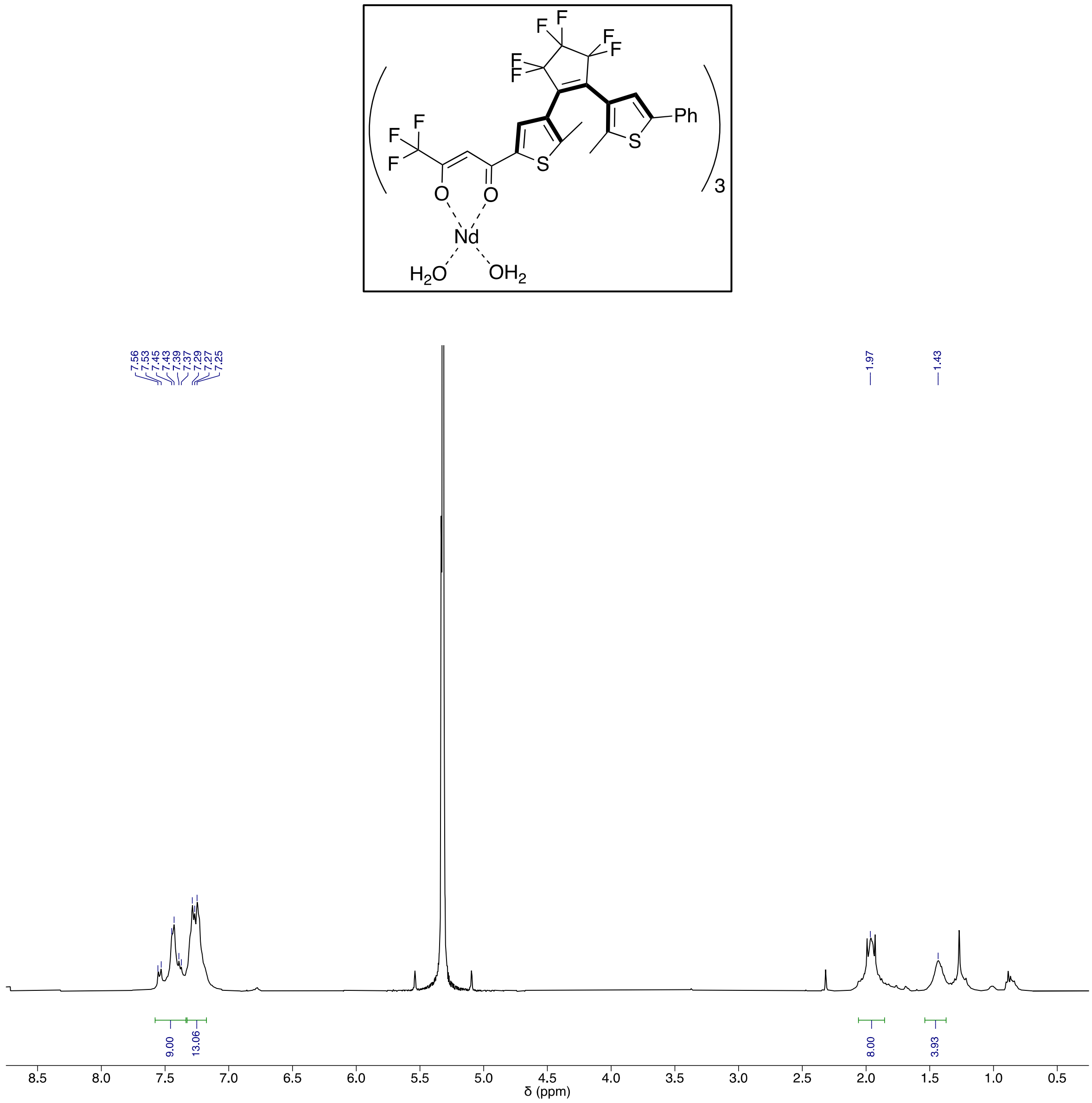

Figure S1a. ^{1}H NMR (400 MHz, CD_2Cl_2) spectrum of compound **2Ndooo**.

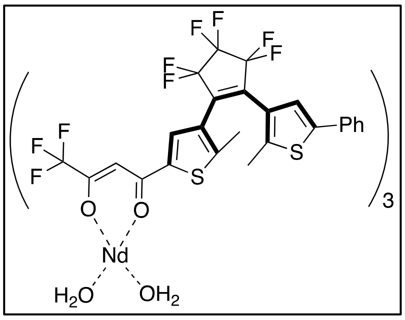

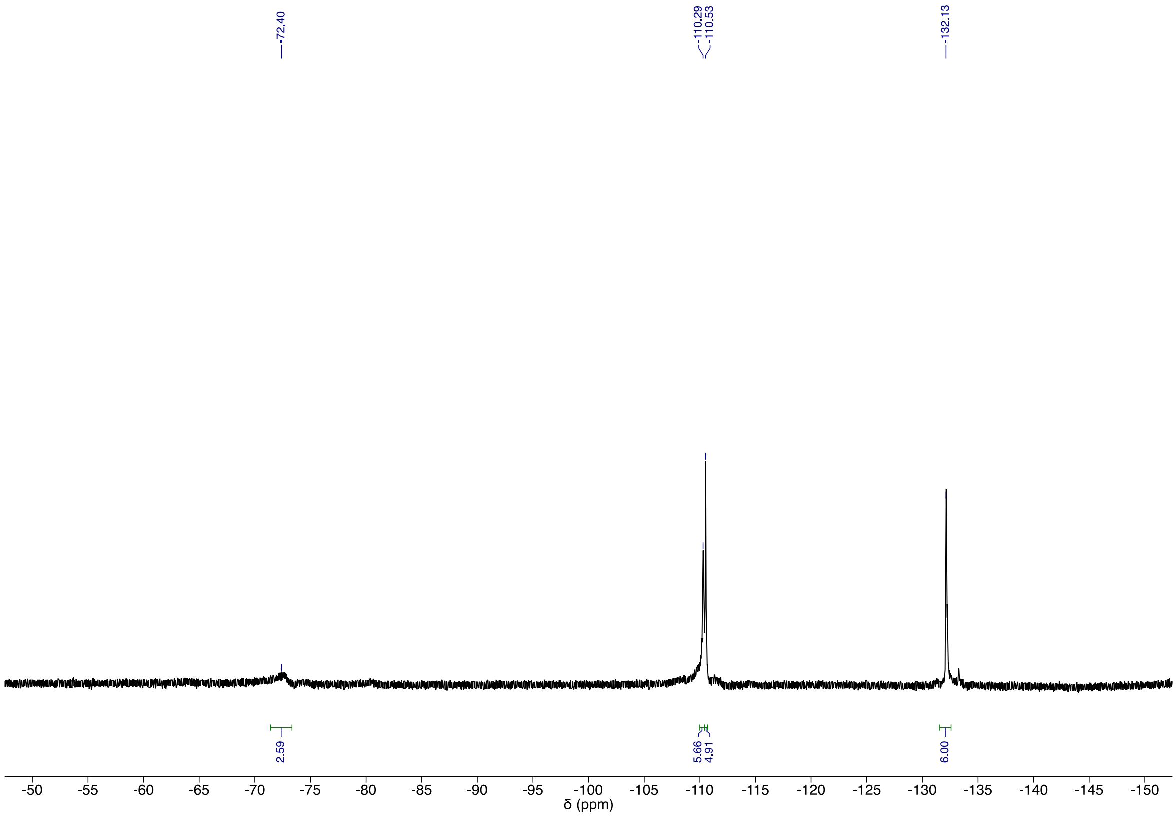

Figure S1b. $^{19}F\{^{1}H\}$ NMR (376 MHz, CD_2Cl_2) spectrum of compound **2Ndooo**.

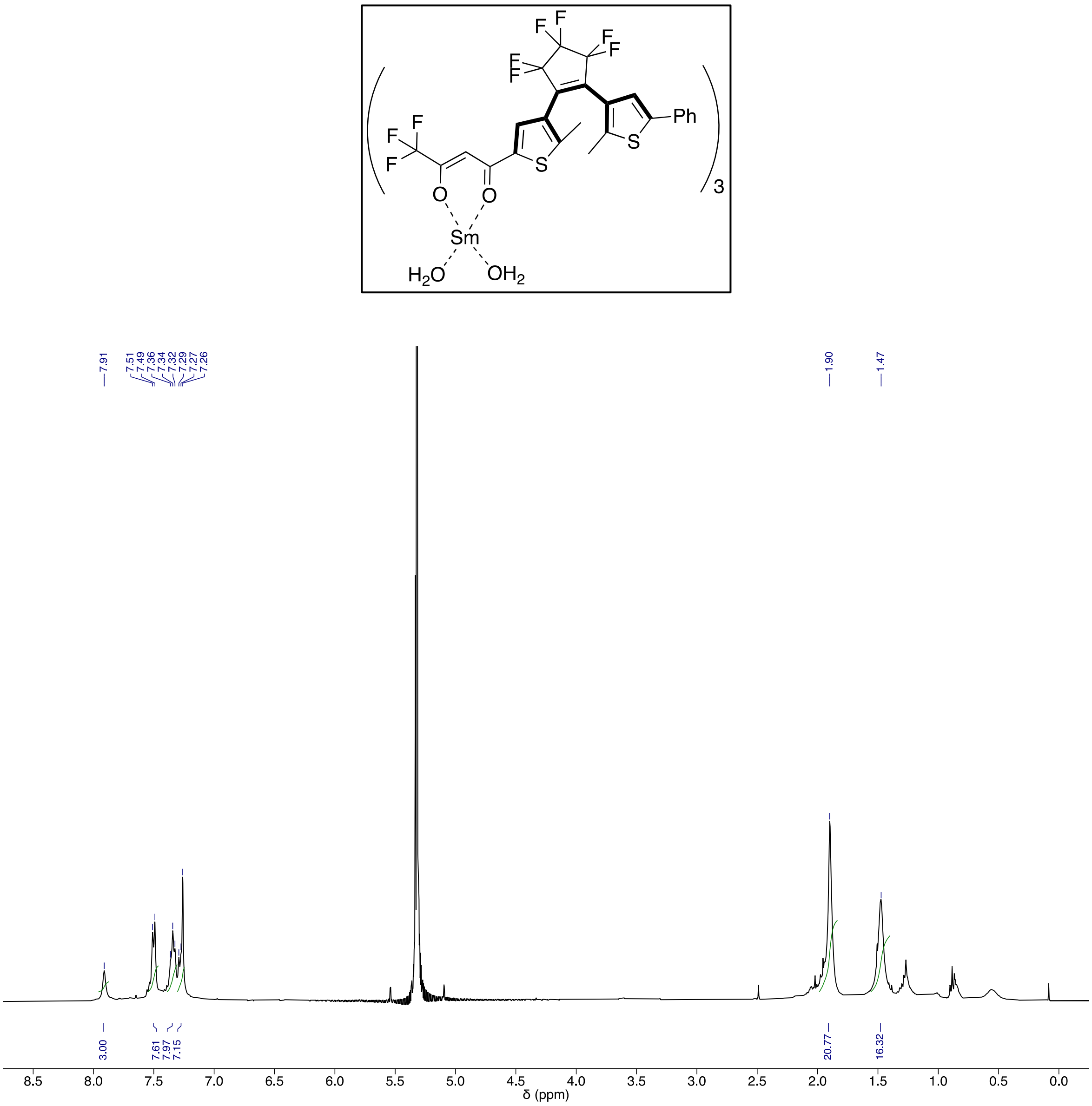

Figure S2a. ^{1}H NMR (400 MHz, CD_2Cl_2) spectrum of compound **2Smooo**.

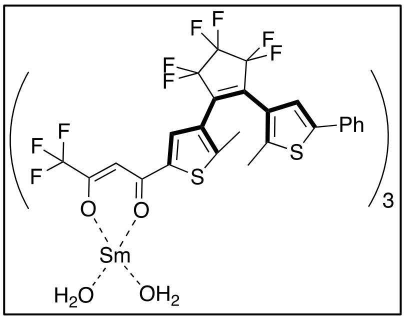

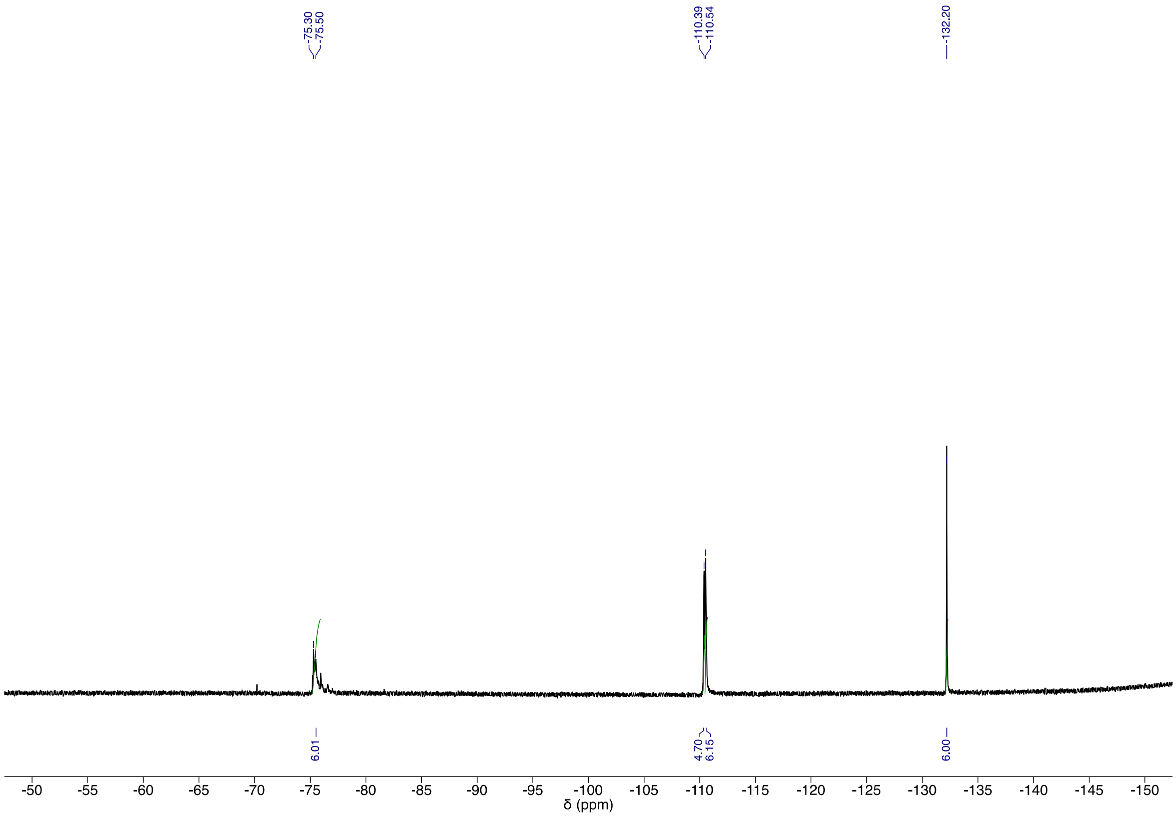

Figure S2b. $^{19}F\{^1H\}$ NMR (376 MHz, CD_2Cl_2) spectrum of compound **2Smooo**.

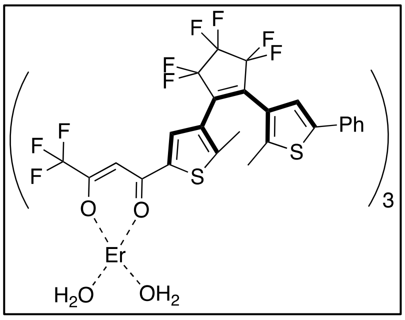

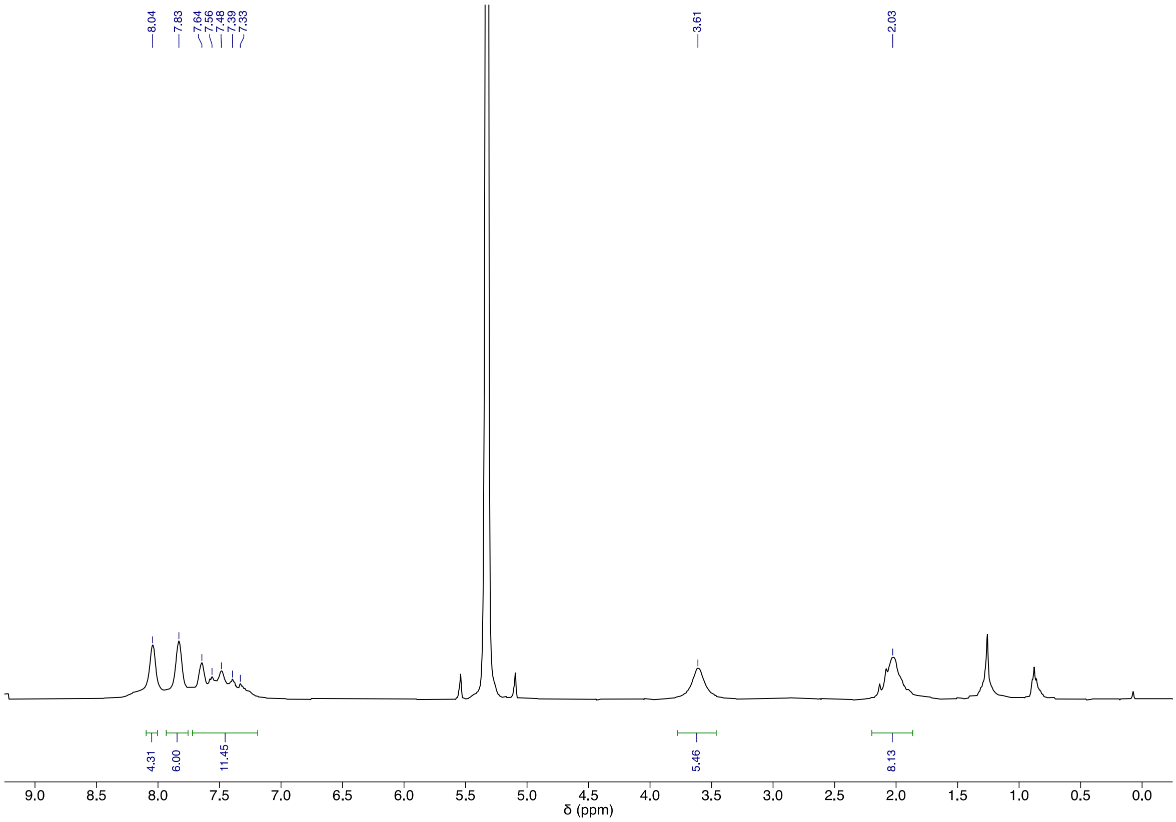

Figure S3a. ^{1}H NMR (400 MHz, CD_2Cl_2) spectrum of compound **2Erooo**.

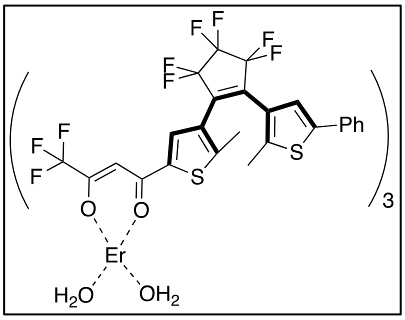

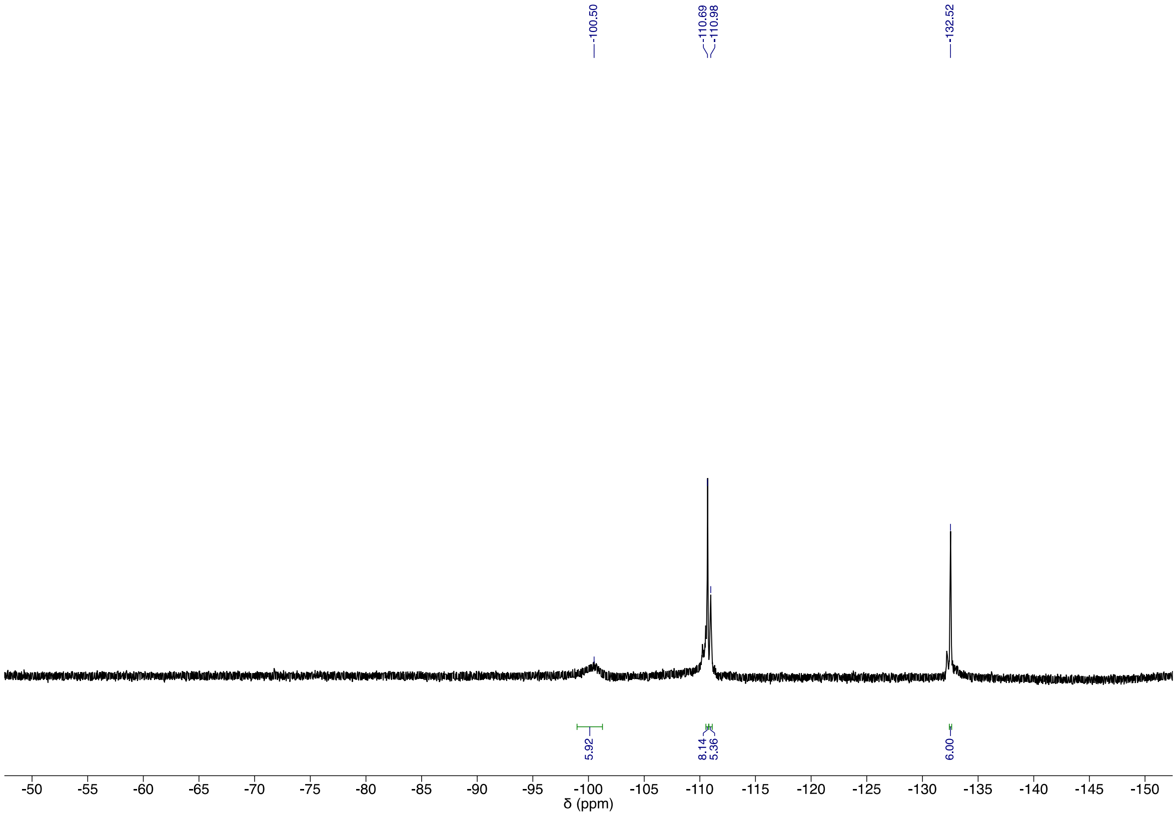

Figure S3b. $^{19}F\{^1H\}$ NMR (376 MHz, CD_2Cl_2) spectrum of compound **2Erooo**.

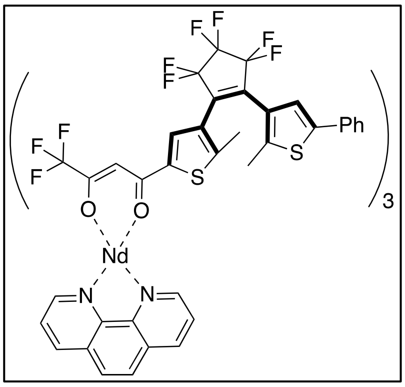

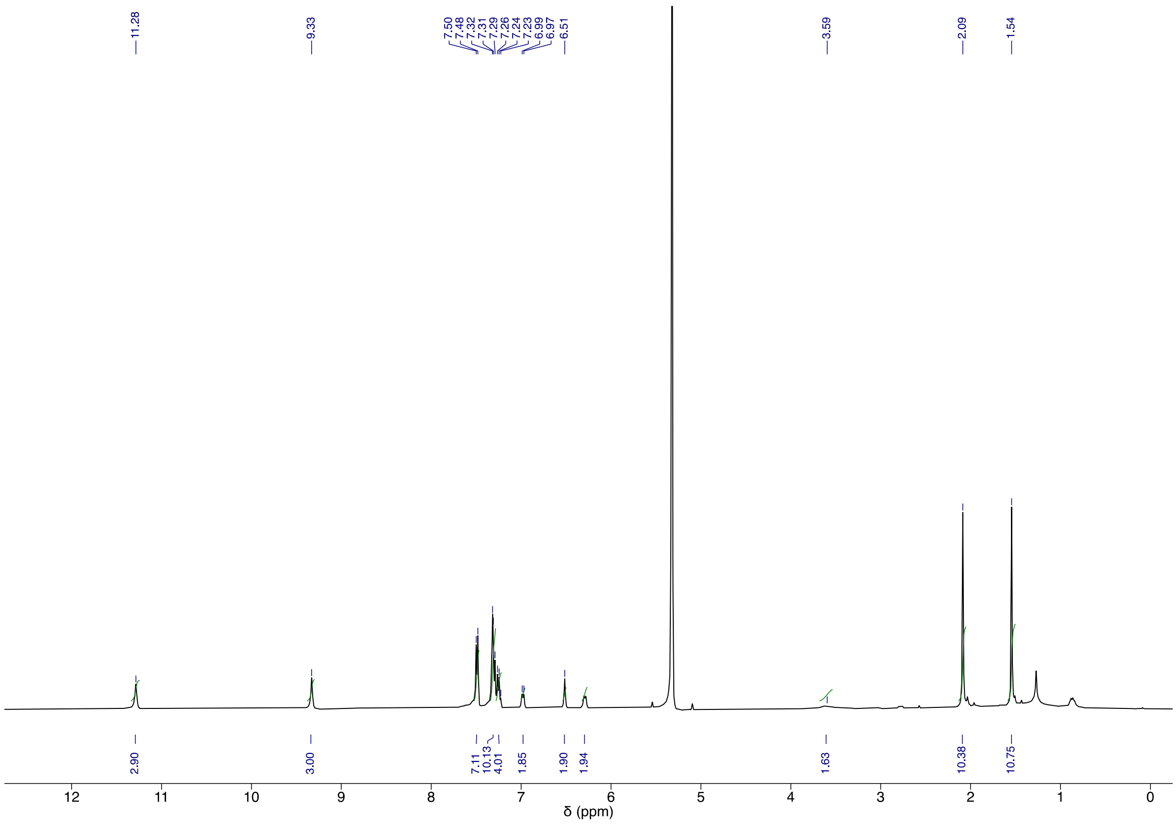

Figure S4a. ^{1}H NMR (400 MHz, CD_2Cl_2) spectrum of compound **2Ndooo**.

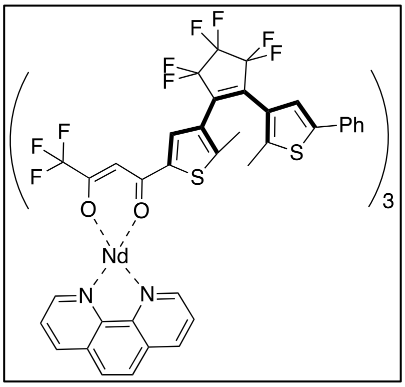

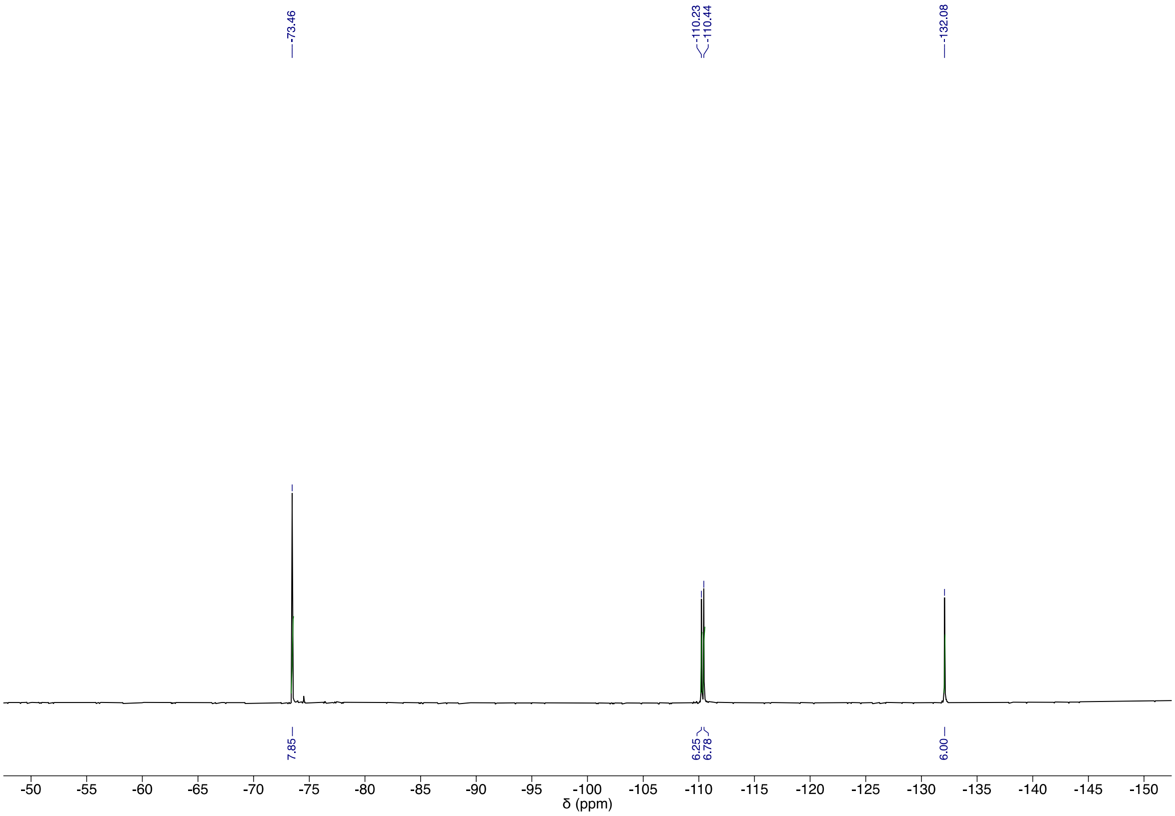

Figure S4b. $^{19}F\{^1H\}$ NMR (376 MHz, CD_2Cl_2) spectrum of compound **3Ndooo**.

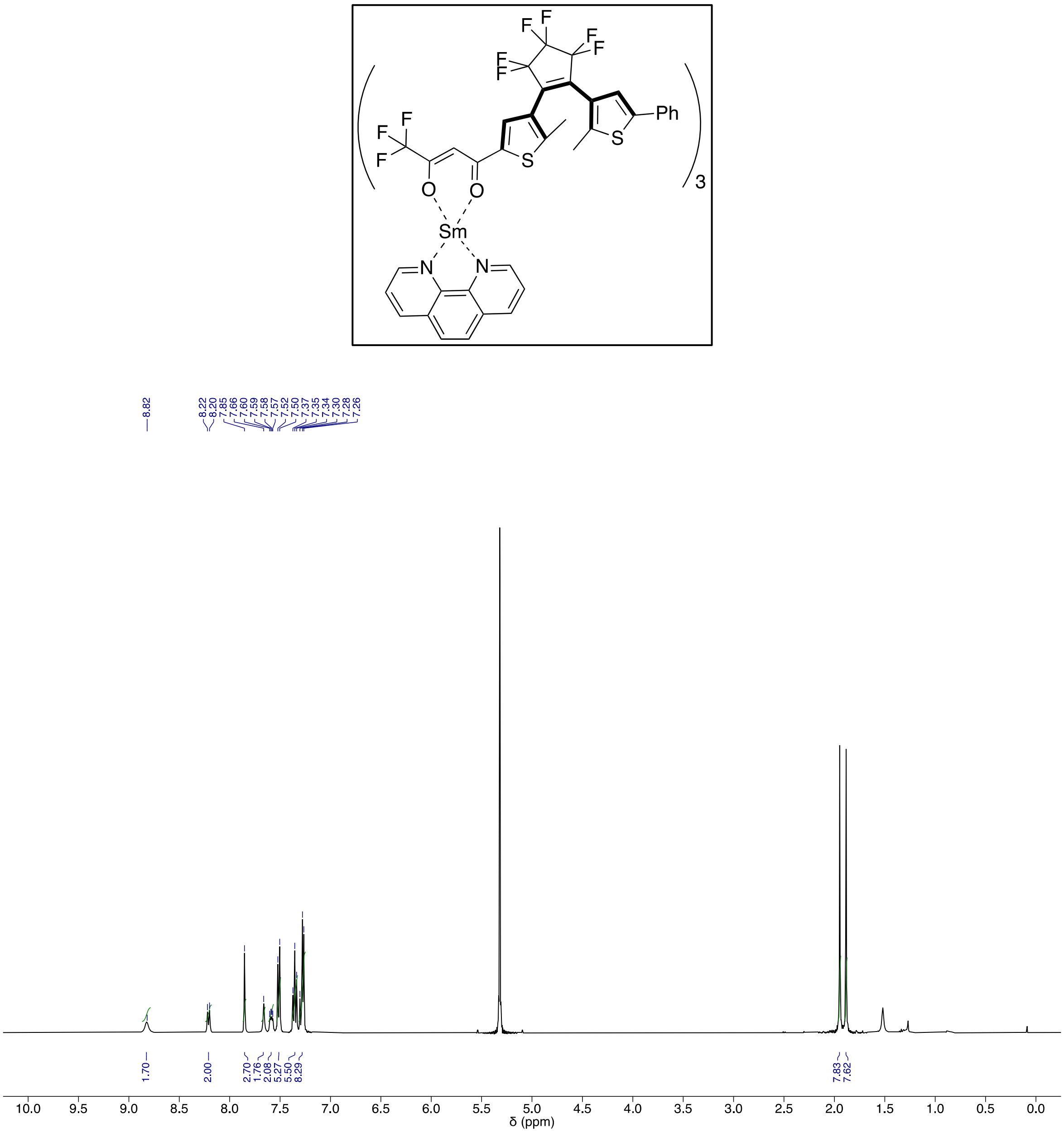

Figure S5a. ^{1}H NMR (400 MHz, CD_2Cl_2) spectrum of compound **3Smooo**.

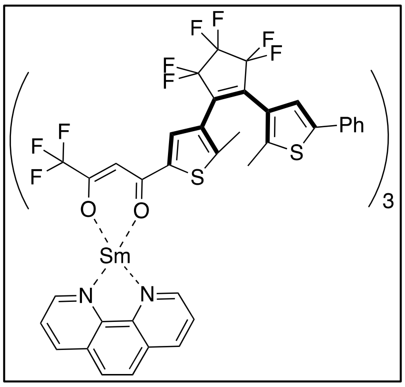

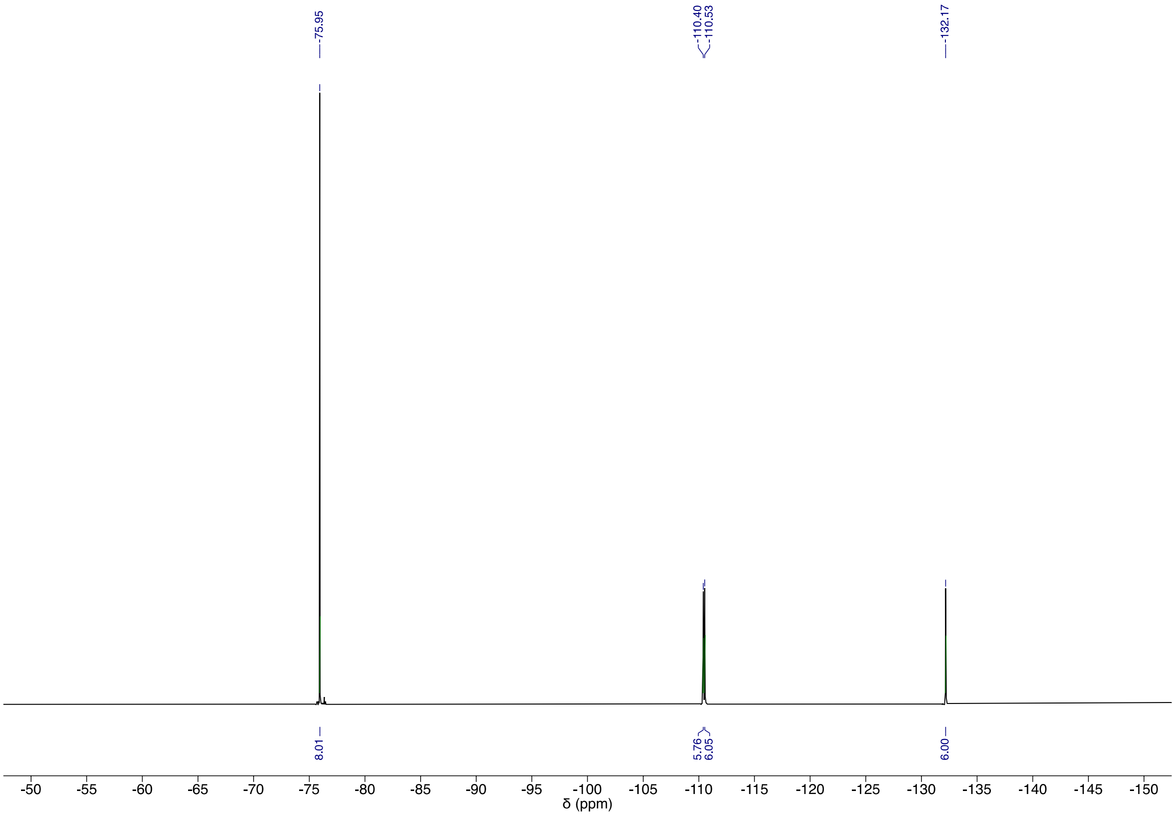

Figure S5b. $^{19}F\{^{1}H\}$ NMR (376 MHz, CD_2Cl_2) spectrum of compound **3Smooo**.

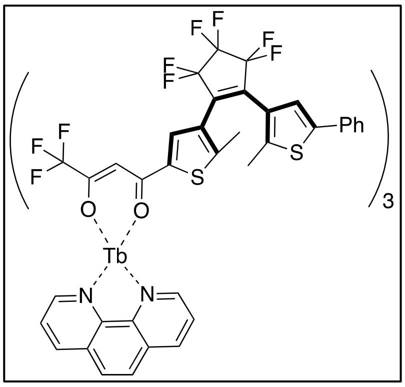

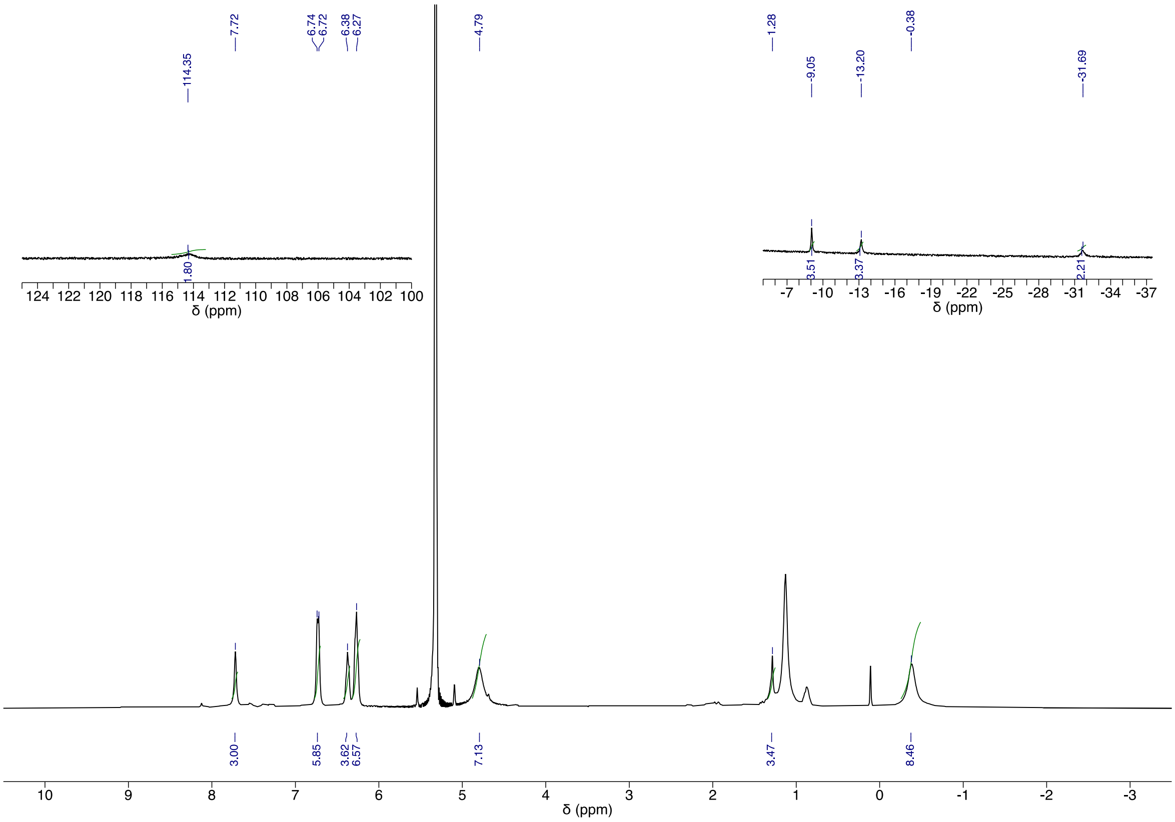

Figure S6a. ^{1}H NMR (400 MHz, CD_2Cl_2) spectrum of compound **3Tbooo**.

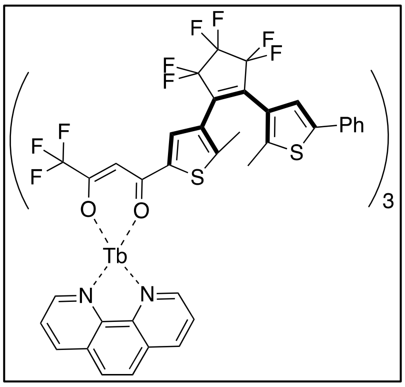

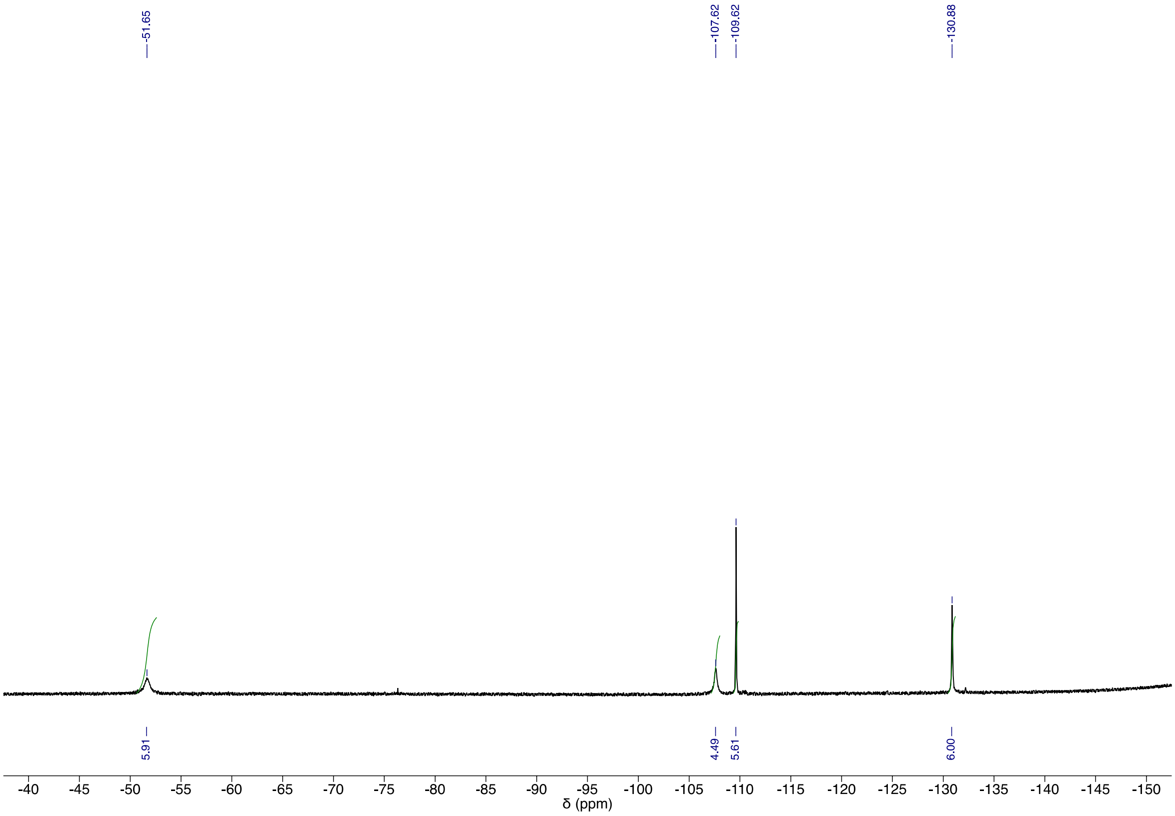

Figure S6b. $^{19}F\{^{1}H\}$ NMR (376 MHz, CD_2Cl_2) spectrum of compound **3Tbooo**.

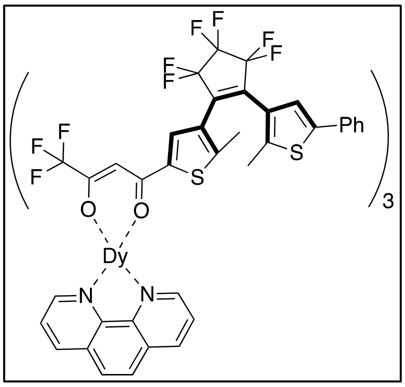

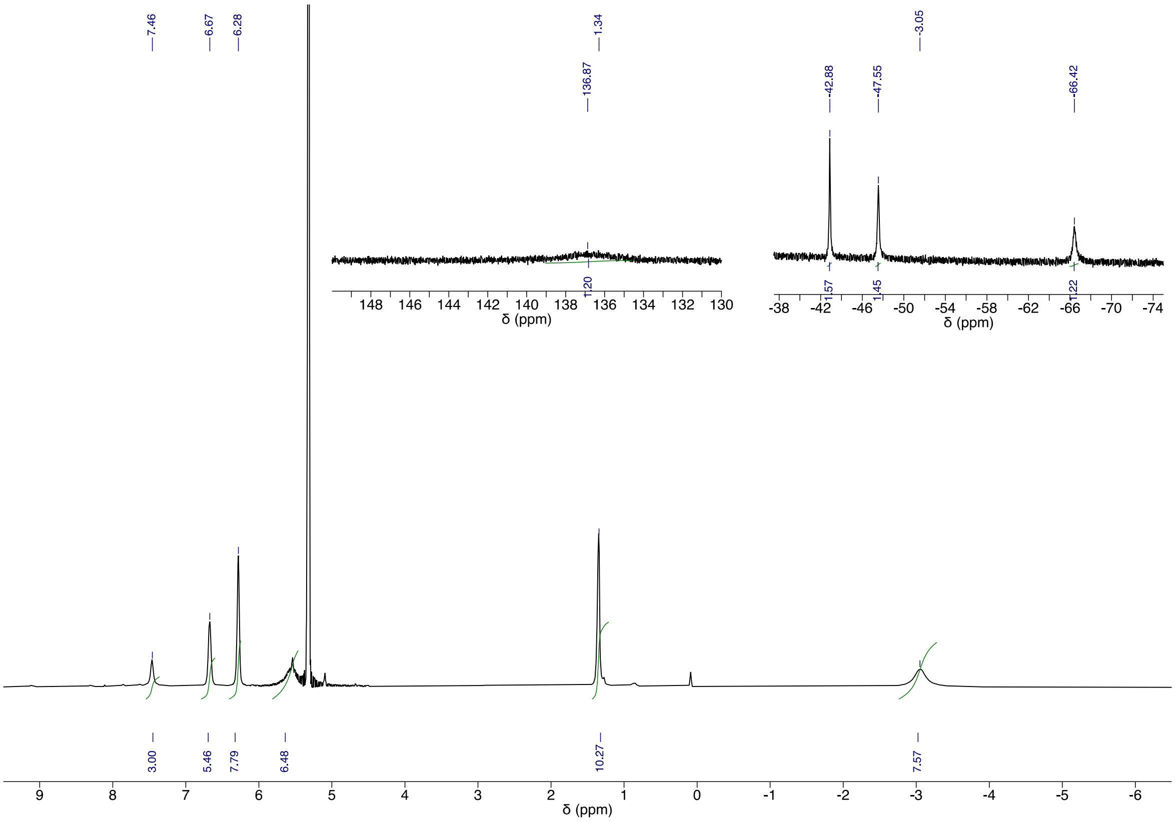

Figure S7a. ^{1}H NMR (400 MHz, CD_2Cl_2) spectrum of compound **3Dyooo**.

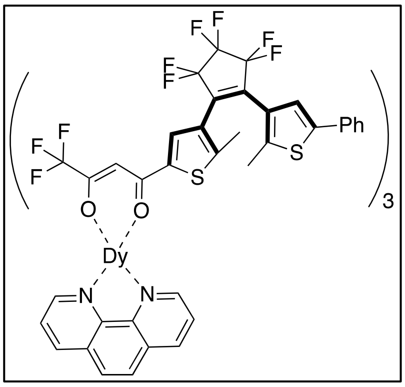

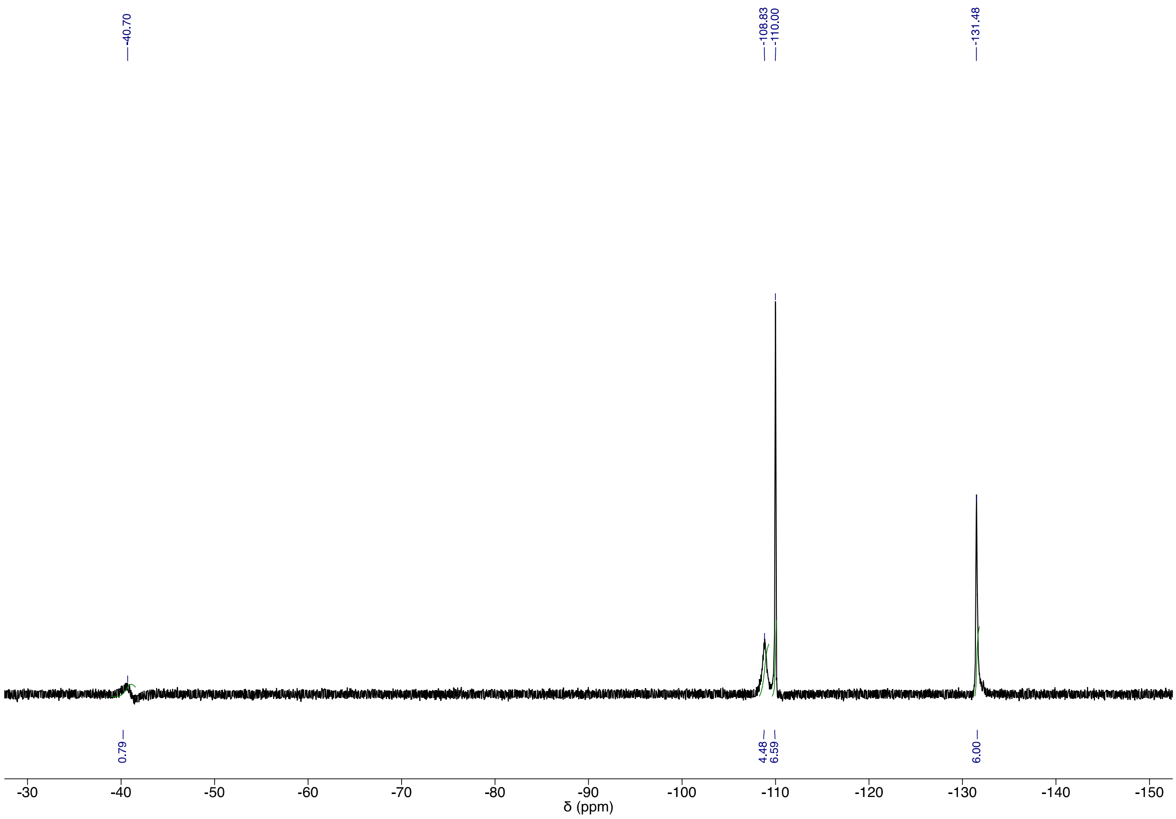

Figure S7b. $^{19}F\{^{1}H\}$ NMR (376 MHz, CD_2Cl_2) spectrum of compound **3Dyooo**.

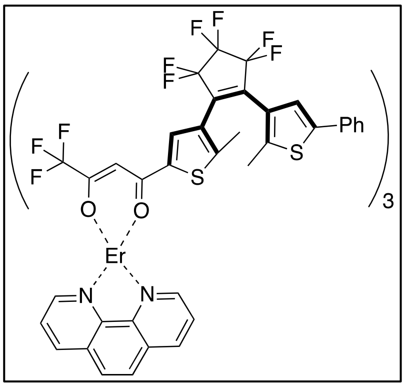

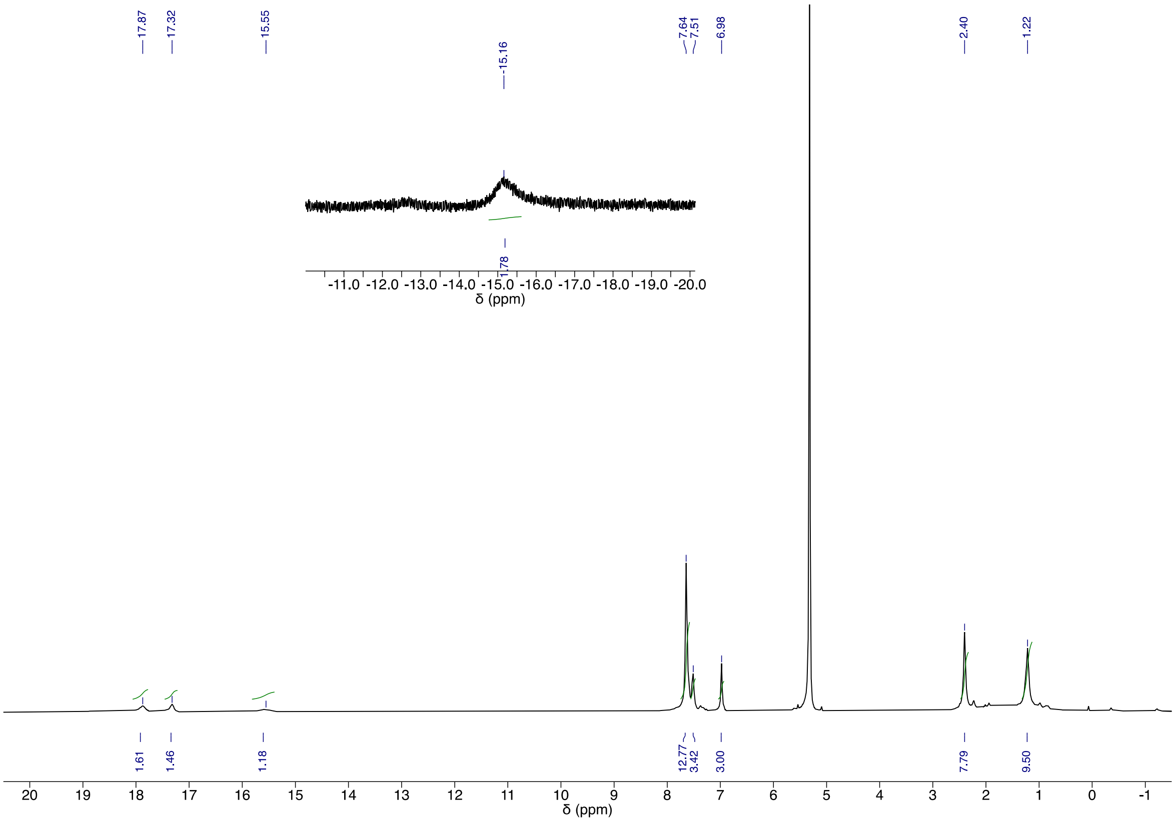

Figure S8a. ^{1}H NMR (400 MHz, CD_2Cl_2) spectrum of compound **3Erooo**.

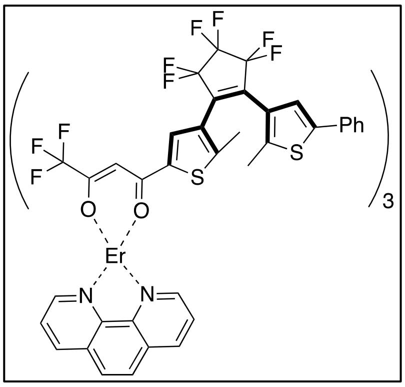

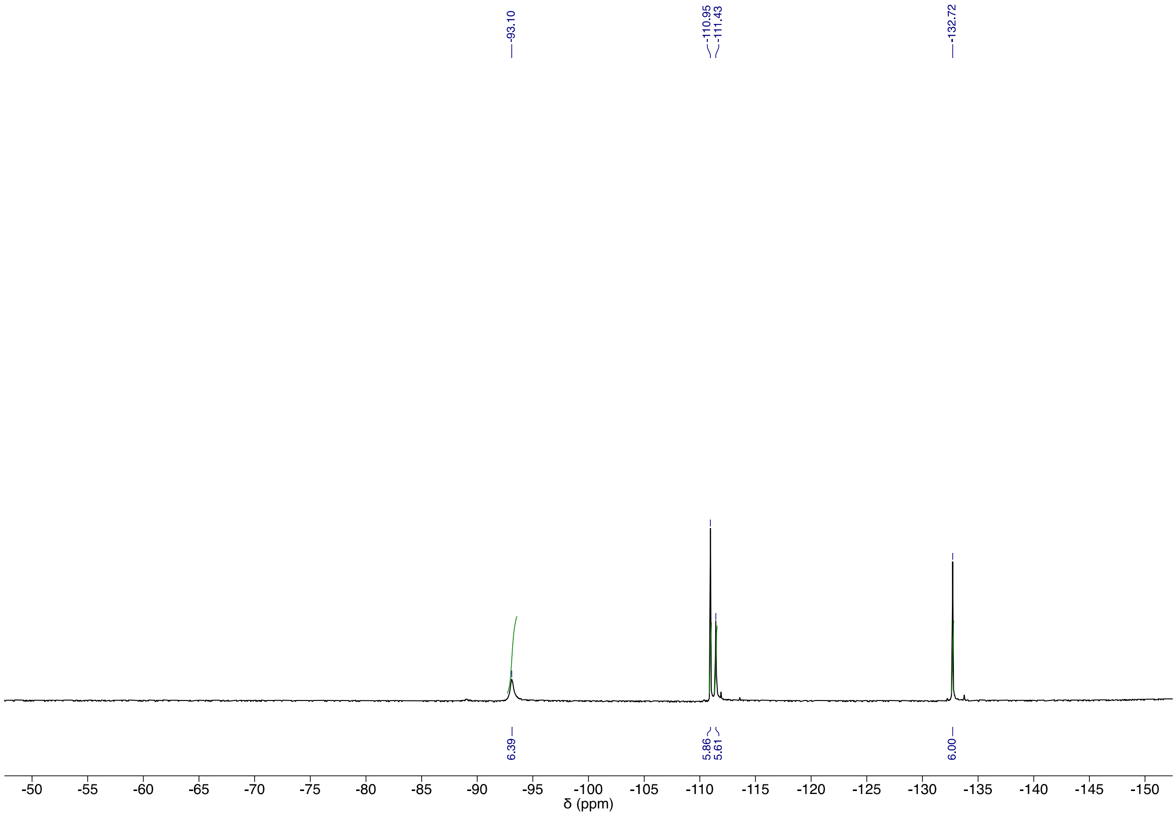

Figure S8b. $^{19}F\{^{1}H\}$ NMR (376 MHz, CD_2Cl_2) spectrum of compound **3Erooo**.

UV-Vis studies and photochromic reactions

General comments: UV-vis measurements were performed in CH_2Cl_2 at 20 °C using a "analytikjena specord 205" spectrophotometer. Routine UV-vis irradiations were performed in UV cells or NMR tubes with a LS series Light Source of ABET technologies, Inc (150 W xenon lamp) equipped with single wavelength light filters "350FS 10-25" and "650FS 10-25". Alternatively, photoirradiations at 365 nm were performed with fluorescent tubes used for TLC illumination, and white light from a 1.5 W LED lamp (λ > 400 nm) was used for opening.

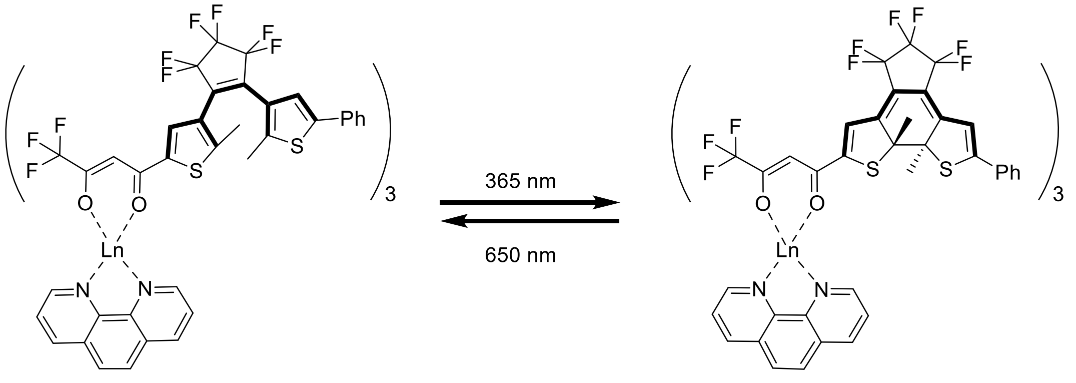

3Ln$_{ooo}$
Ln = Nd, Sm, Gd, Tb, Dy, Er

3Ln$_{ccc}$
Ln = Nd, Sm, Gd, Tb, Dy, Er

For complex **3Ndooo**:

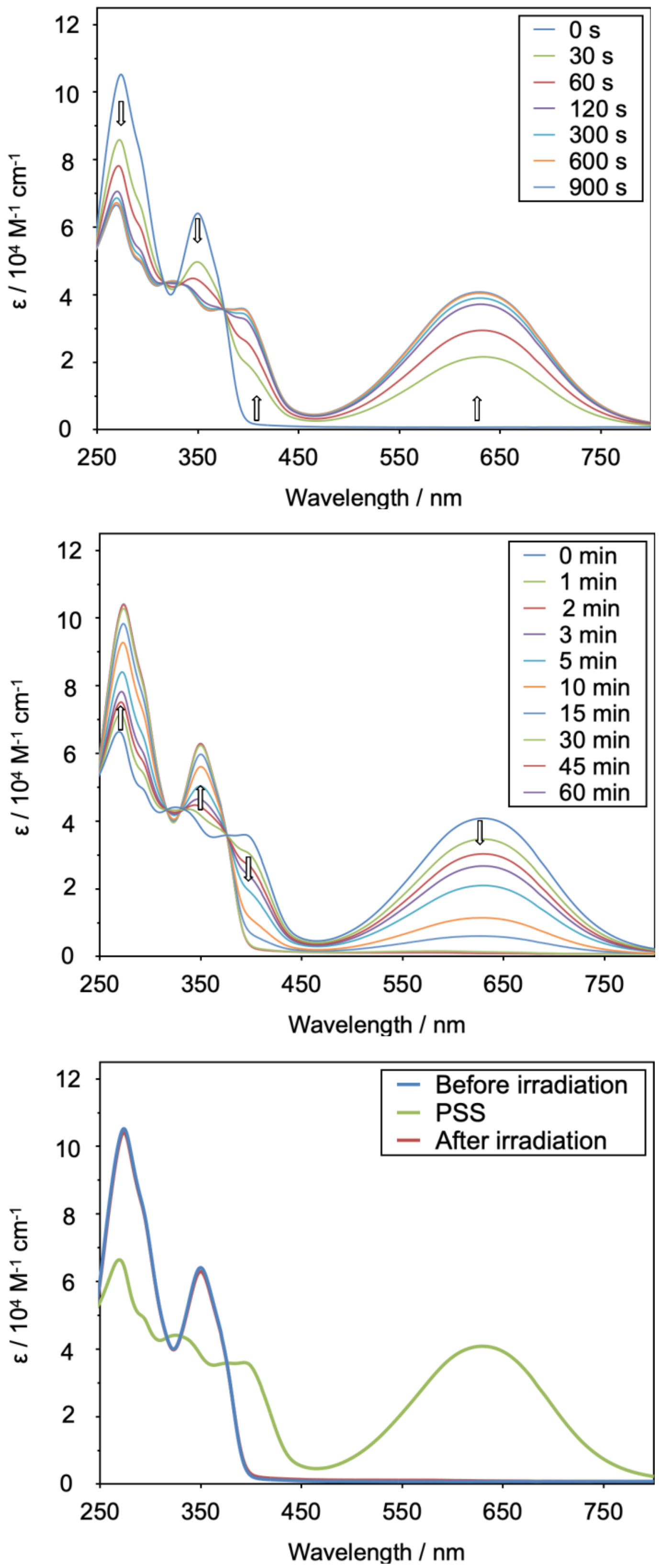

Figure S9. Absorption spectra of **3Ndooo** in CH_2Cl_2 solutions ([c]~5×10^{-6} $M^{-1}.cm^{-1}$) upon closing (top) and opening (middle) of the system under UV and vis irradiations, and the evolution of the absorption spectrum after one cycle showing full reversibility (bottom).

For complex **3Smooo**:

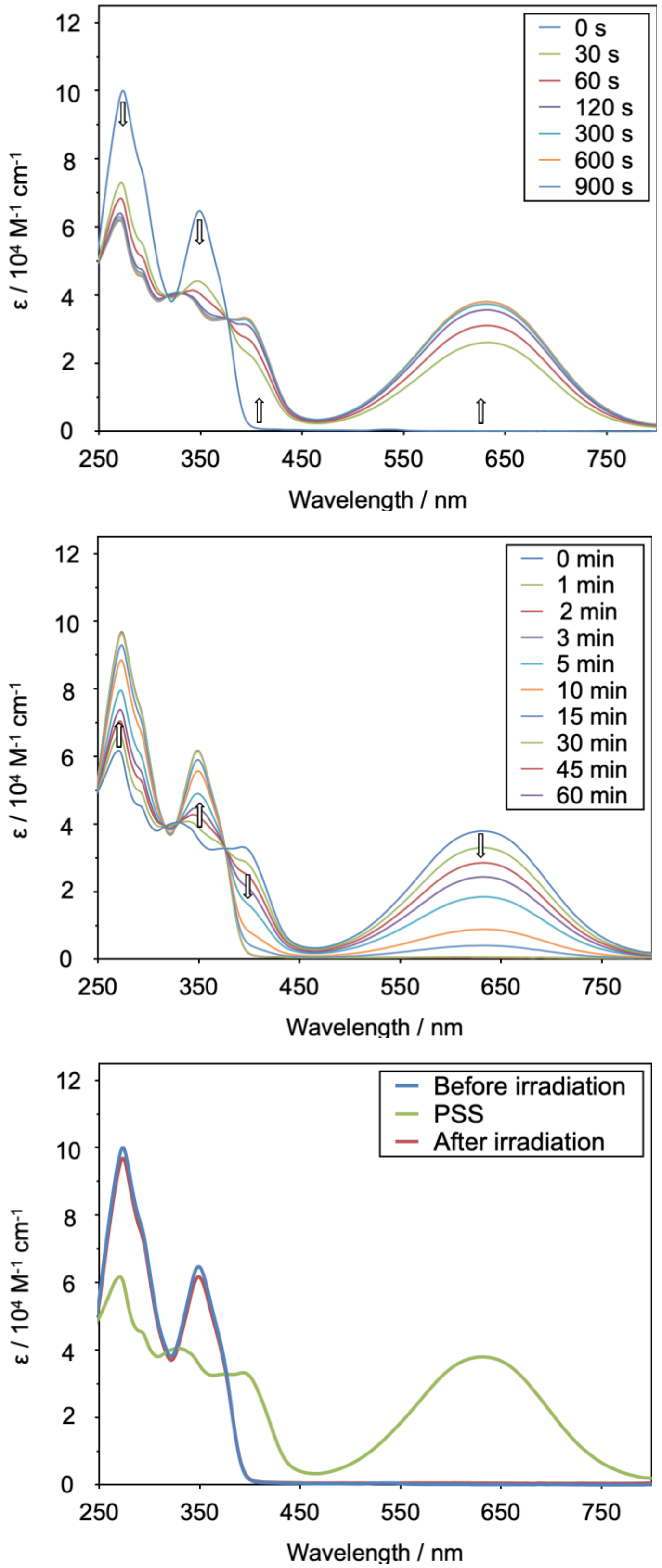

Figure S10. Absorption spectra of **3Smooo** in CH_2Cl_2 solutions ([c]~5×10^{-6} M^{-1}.cm^{-1}) upon closing (top) and opening (middle) of the system UV and vis irradiations, and the evolution of the absorption spectrum after one cycle showing full reversibility (bottom).

For complex **3Gdooo**:

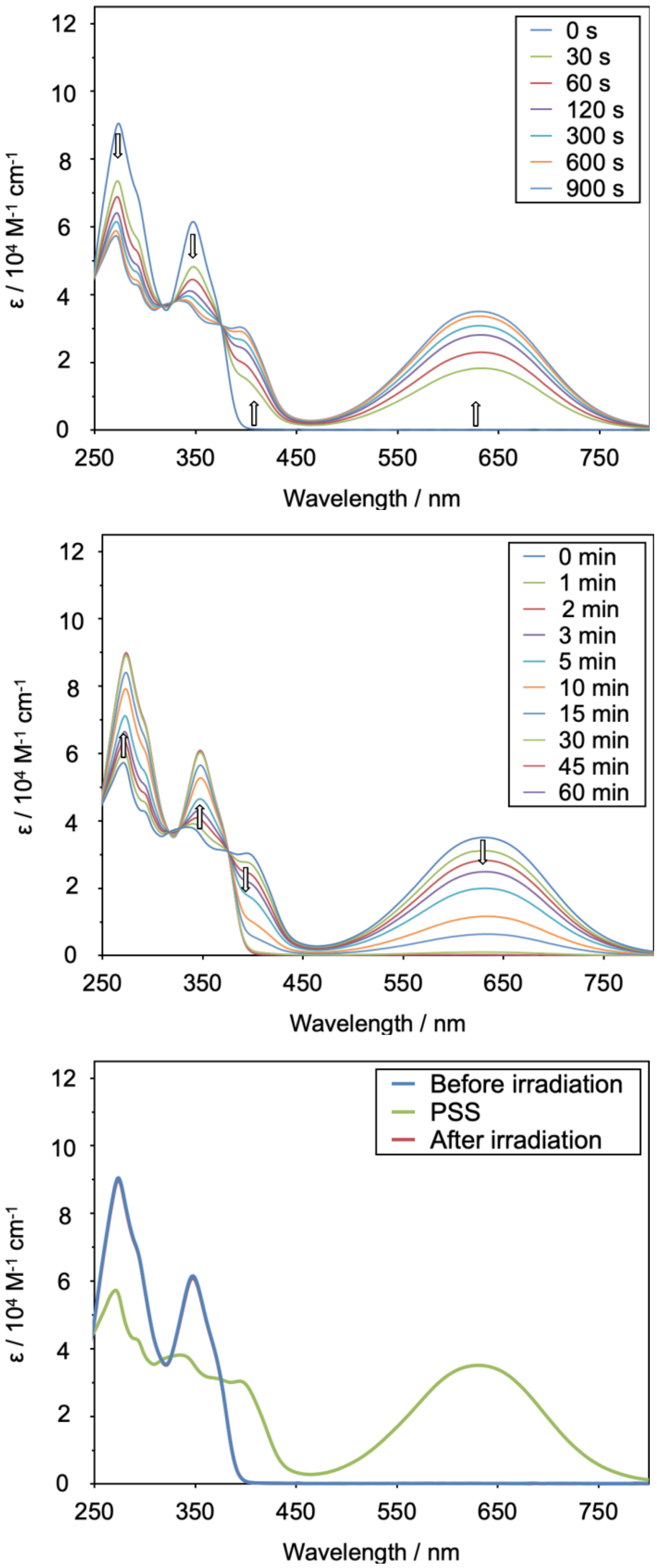

Figure S11. Absorption spectra of **3Gdooo** in CH_2Cl_2 solutions ([c]~5×10^{-6} $M^{-1}.cm^{-1}$) upon closing (top) and opening (middle) of the system UV and vis irradiations, and the evolution of the absorption spectrum after one cycle showing full reversibility (bottom).

For complex **3Tbooo**:

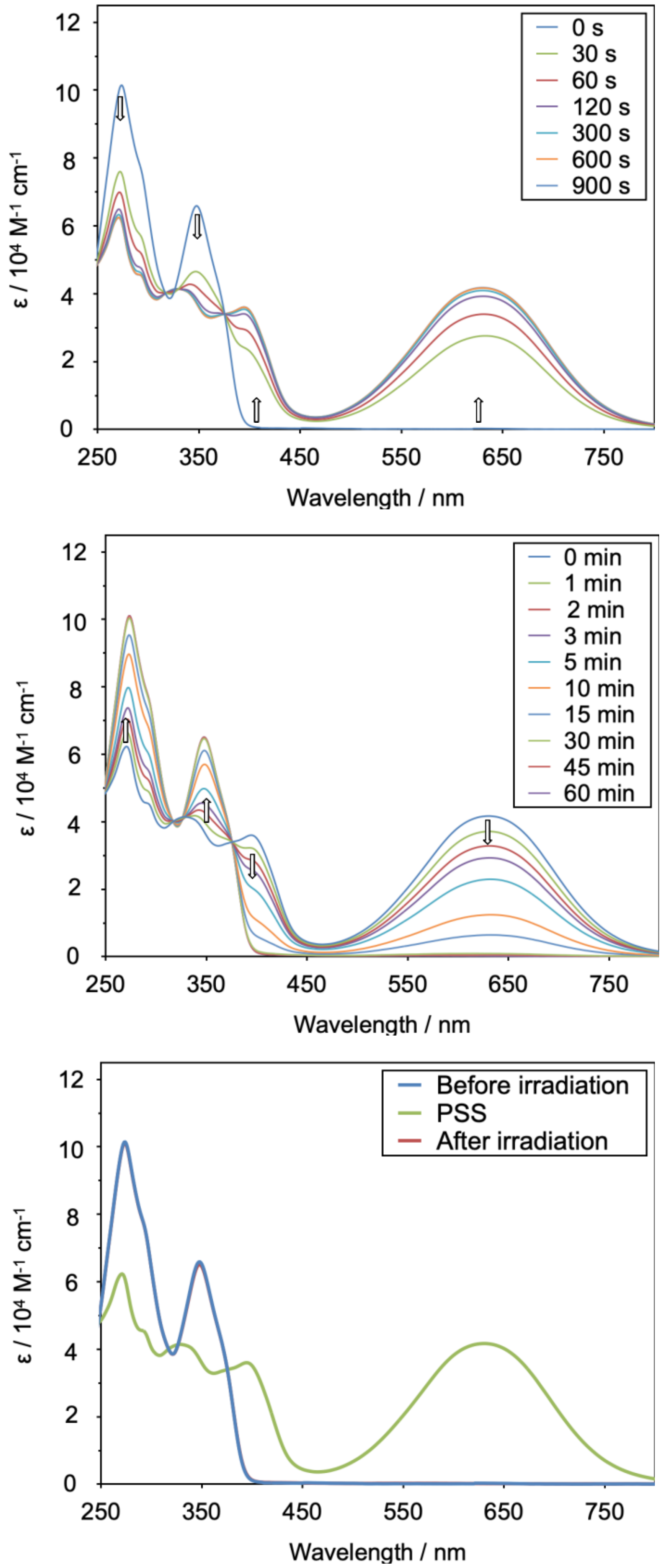

Figure S12. Absorption spectra of **3Tbooo** in CH_2Cl_2 solutions ([c]~5×10^{-6} M^{-1}.cm^{-1}) upon closing (top) and opening (middle) of the system UV and vis irradiations, and the evolution of the absorption spectrum after one cycle showing full reversibility (bottom).

For complex **3Dyooo**:

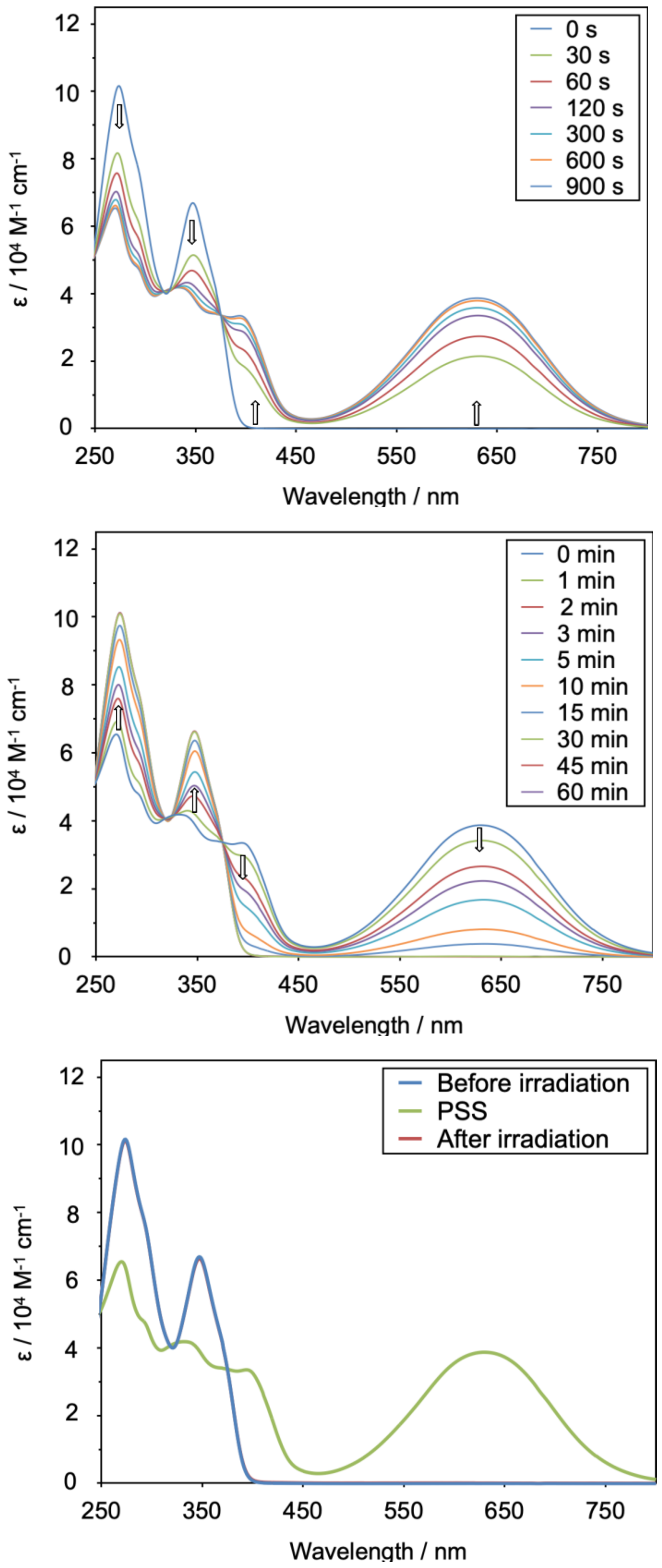

Figure S13. Absorption spectra of **3Dyooo** in CH_2Cl_2 solutions ([c]~5×10^{-6} $M^{-1}.cm^{-1}$) upon closing (top) and opening (middle) of the system UV and vis irradiations, and the evolution of the absorption spectrum after one cycle showing full reversibility (bottom).

For complex **3Erooo**:

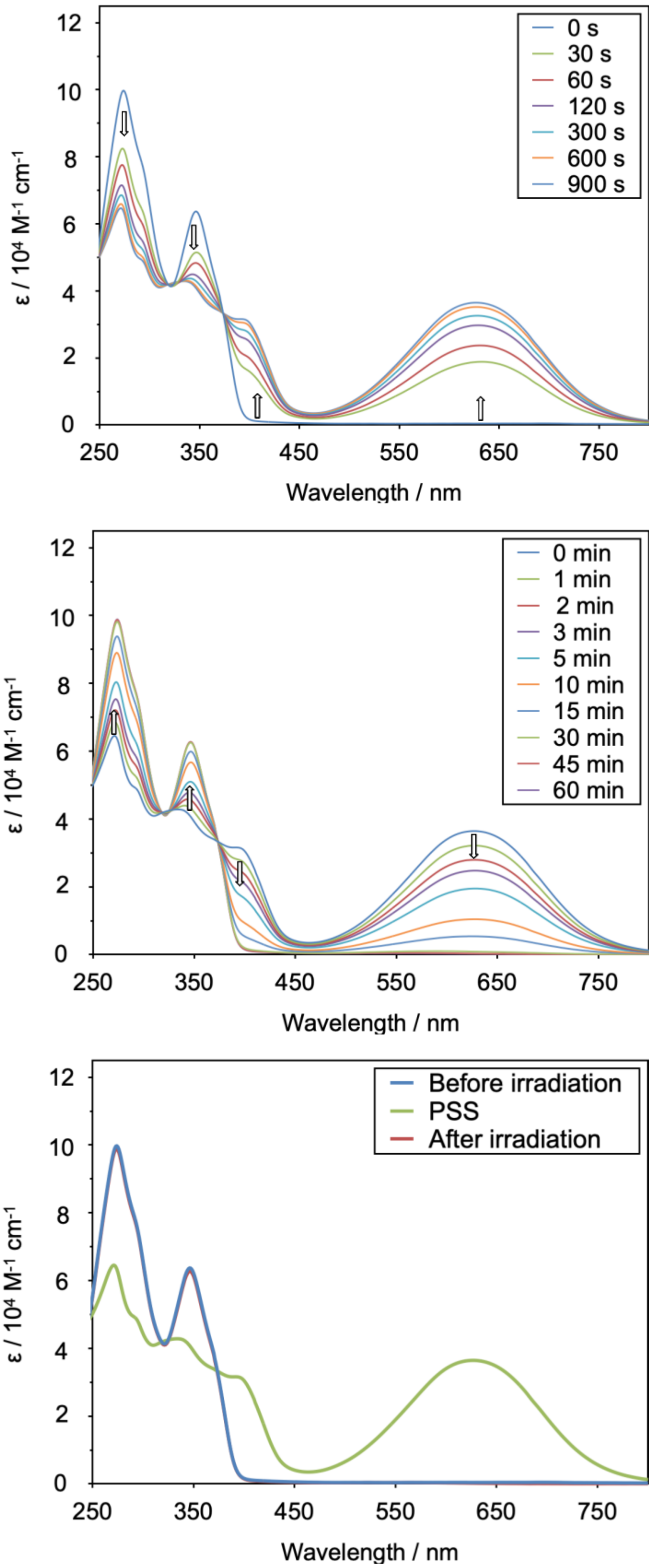

Figure S14. Absorption spectra of **3Erooo** in CH_2Cl_2 solutions ([c]~5×10^{-6} $M^{-1}.cm^{-1}$) upon closing (top) and opening (middle) of the system UV and vis irradiations, and the time evolution of the absorption spectra (bottom).

NMR CONVERSIONS

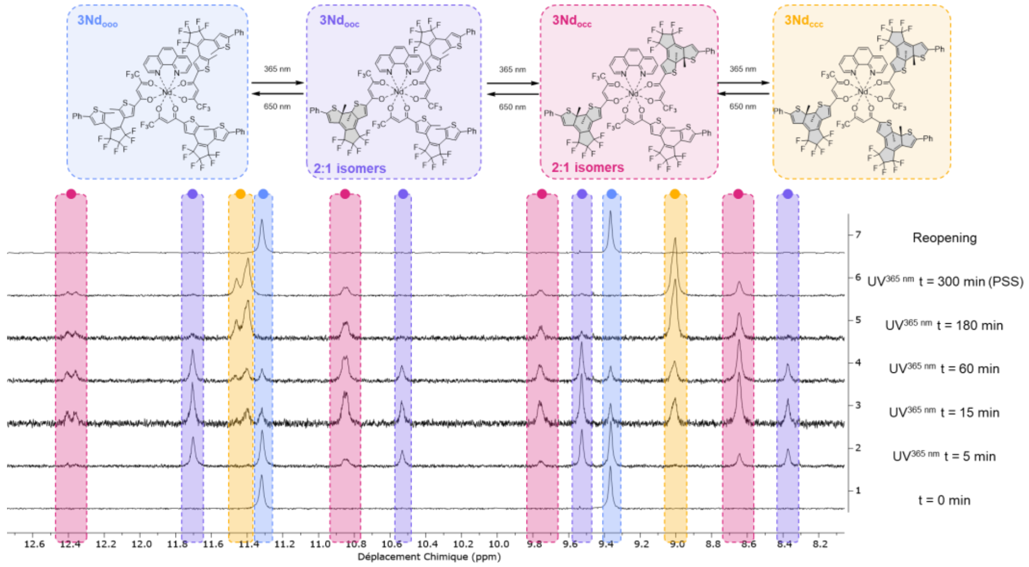

% Conversion	Nd ooo	Nd ooc	Nd occ	Nd ccc
0,00	1,00	0,00	0,00	0,00
0,26	0,38	0,46	0,16	0,00
0,55	0,07	0,35	0,44	0,14
0,56	0,07	0,33	0,45	0,15
0,87	0,00	0,03	0,33	0,64
0,93	0,00	0,00	0,22	0,78

Figure S15: Evolution of pyridine ^{1}H NMR signals of **3Nd$_{ooo}$** in CD_2Cl_2 upon irradiation al λ = 350 nm for 300 minutes. The integrations of the signals of the different forms allow the determination of the amounts of ooo/ooc/occ/ccc species, in addition to the global DTE conversion. The assignments are based on the time evolution. The initial spectrum was recovered after irradiation at λ = 650 nm.

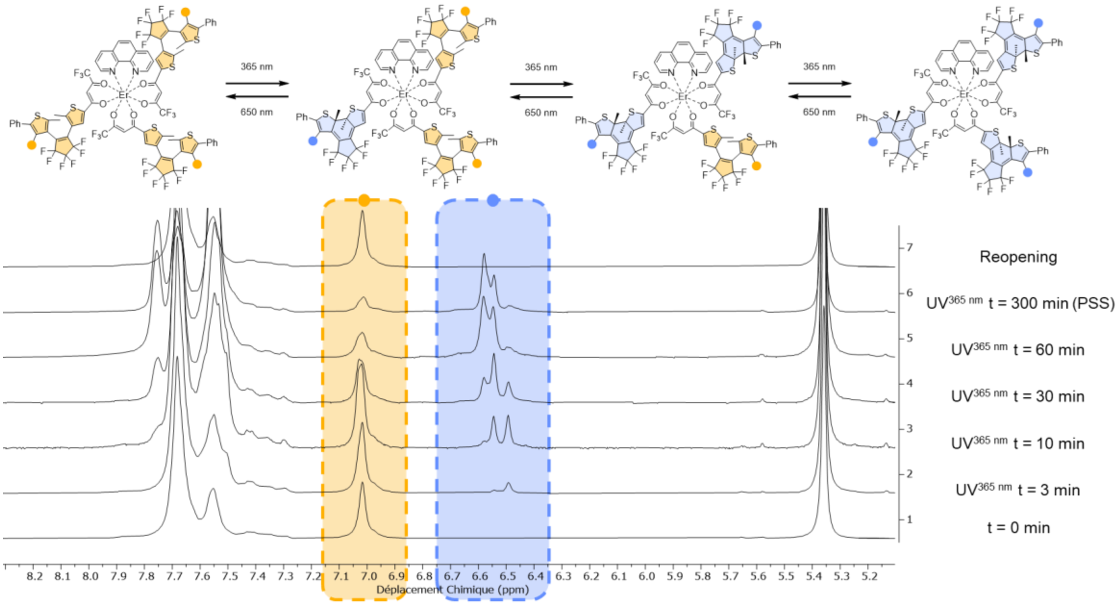

UV irradiation	1H 7,15-6,85 ppm	1H 6,35-6,75 ppm	% Conversion
start	1	0	0,00
3 min	1	0,13	0,12
10 min	1	0,48	0,32
30 min	1	1,20	0,55
60 min	1	3,88	0,80
300 min	1	6,65	0,87

Figure S16: Evolution of methyl groups ^{1}H NMR signals of **3Er$_{ooo}$** in CD_2Cl_2 upon irradiation al λ = 350 nm for 300 minutes. The global integrations of the signals of the open (yellow) and closed (blue) DTE isomers allow the determination of the globaln amount of cloded DTE formed withoue distinction of the ooc/occ/ccc species. The initial spectrum was recovered after irradiation at λ = 650 nm.

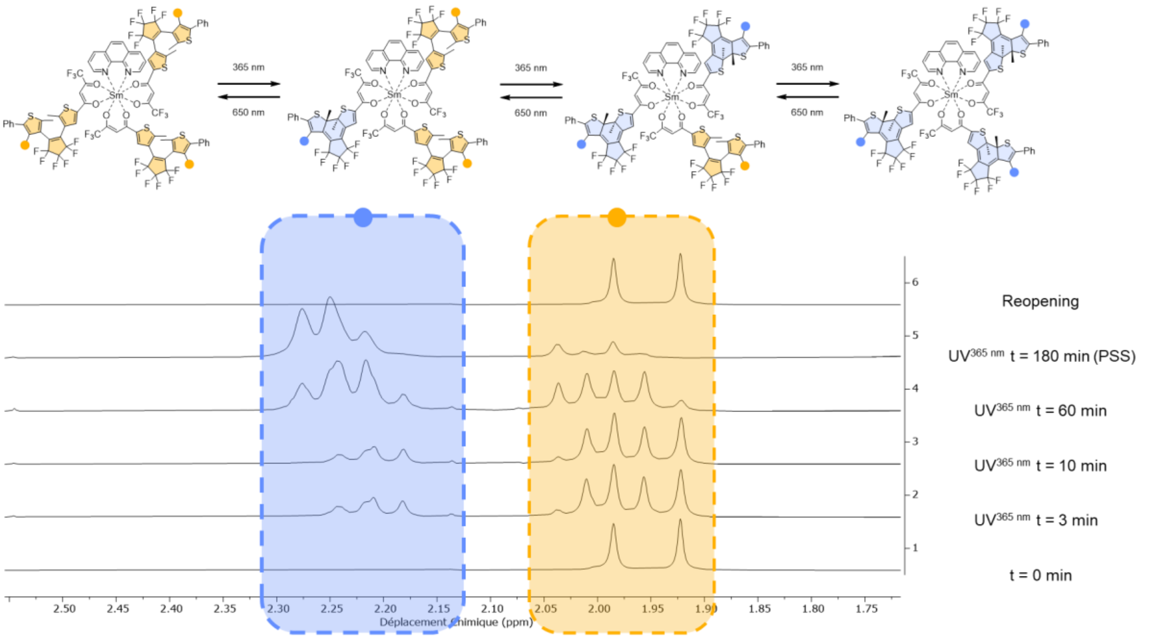

UV irradiation	1H 1,87-2,07 ppm	1H 2,12-2,32 ppm	% Conversion
start	1	0	0,00
3 min	1	0,3	0,23
10 min	1	0,78	0,44
60 min	1	3,88	0,80
300 min	1	7,77	0,89

Figure S17: Evolution of methyl groups ^{1}H NMR signals of **3Sm$_{ooo}$** in CD_2Cl_2 upon irradiation al λ = 350 nm for 300 minutes. The global integrations of the signals of the open (yellow) and closed (blue) DTE isomers allow the determination of the globaln amount of cloded DTE formed withoue distinction of the ooc/occ/ccc species. The initial spectrum was recovered after irradiation at λ = 650 nm.

PHOTOKINETIC EXPERIMENTS: Absoprtion time-profiles acquisition and quantum yields determination.

Time-profiles were obtained on a homemade photokinetic setup, by monitoring the change of the absorption spectrum overtime, while the sample is continuously irradiated at 365 nm or at 577 nm, under continuous stirring (400 rpm) at T=20°C. The temperature and stirring were controlled thanks to Peltier device. The irradiation wavelength (source Xe lamp Zolix instruments, model Sitius 300P) was selected by passing through a monochromator (Zolix instruments, model Omni-λ200i, halfwidth of ±10 nm). Absorption spectra were acquired with a Flame spectrophotometer (Ocean Insights) combined to a DHBAL-2000 D/Hal lamp. Light intensity was measured using a Thorlabs (PM100USB) powermeter. Experimental data are a matrix of full spectra over the full time of acquisition; useful photokinetic profiles were extracted at the maximum of absorbance of the corresponding close form.

The time-profiles were fitted (homemade Igor Pro 9 algorithm procedure (Wavemetrics)) to the photokinetic equations by means of a numerical iterative fitting method.

The photokinetic equations are

$$[\mathbf{ooo}] \underset{\Phi_{c\to o}}{\overset{\Phi_{ooo\to ooc}}{\rightleftarrows}} [\mathbf{ooc}] \underset{\Phi_{c\to o}}{\overset{\Phi_{ooc\to occ}}{\rightleftarrows}} [\mathbf{occ}] \underset{\Phi_{c\to o}}{\overset{\Phi_{occ\to ccc}}{\rightleftarrows}} [\mathbf{ccc}]$$

$$\frac{d[\mathbf{ooo}]}{dt} = \Phi_{c\to o} \times I_{\mathbf{ooc}}^{abs-c} - \Phi_{ooo\to ooc} \times I_{\mathbf{ooo}}^{abs-o}$$

$$\frac{d[\mathbf{ooc}]}{dt} = \Phi_{ooo\to ooc} \times I_{\mathbf{ooo}}^{abs-o} + \Phi_{c\to o} \times I_{\mathbf{occ}}^{abs-c} - \Phi_{c\to o} \times I_{\mathbf{ooc}}^{abs-c} - \Phi_{ooc\to occ} \times I_{\mathbf{ooc}}^{abs-o}$$

$$\frac{d[\mathbf{occ}]}{dt} = \Phi_{ooc\to occ} \times I_{\mathbf{ooc}}^{abs-o} + \Phi_{c\to o} \times I_{\mathbf{ccc}}^{abs-c} - \Phi_{c\to o} \times I_{\mathbf{occ}}^{abs-c} - \Phi_{occ\to ccc} \times I_{\mathbf{occ}}^{abs-o}$$

$$\frac{d[\mathbf{ccc}]}{dt} = \Phi_{occ\to ccc} \times I_{\mathbf{occ}}^{abs-o} - \Phi_{c\to o} \times I_{\mathbf{ccc}}^{abs-c}$$

$$\text{with } I_{\mathbf{tot}}^{abs} = I_{\mathbf{ooo}}^{abs-o} + I_{\mathbf{ooo}}^{abs-c} + I_{\mathbf{ooc}}^{abs-o} + I_{\mathbf{ooc}}^{abs-c} + I_{\mathbf{occ}}^{abs-o} + I_{\mathbf{occ}}^{abs-c} + I_{\mathbf{ccc}}^{abs-o} + I_{\mathbf{ccc}}^{abs-c}$$

where I_i^{abs-x} is the intensity absorbed by the DTEs in the ***x*** form (either ***o*** or ***c***) in the isomer *i* and $\phi_{i\to j}$ is the i→j photochromic quantum yield (I_{tot} is the total absorbed light).

The ring opening (cycloreversion) quantum yields were determined by recording the absorption time-profiles of the corresponding closed isomer upon irradiation at 577 nm. The time-profiles were fitted with a monoexponential decay function of the kind

(Eq. S1) $$\boldsymbol{y = y_0 + A.e^{-\frac{t}{t_1}}}$$

The cycloreversion quantum yields were obtained from eq. S2

(Eq. S2) $$\boldsymbol{\phi_{c/o} = \frac{-a^{mol}}{\varepsilon_{CF}^{mol}(obs)*I_0\left(1-10^{-\left(A_0(irr)\right)}\right)*l}}$$

Where a^{mol} represents the slope of the kinetic curve at the beginning of the process and is obtained from $a^{mol} = -\frac{A}{t_1}$, $\varepsilon_{CF}^{mol}(obs)$ is the extinction coefficient of the closed isomer at the observation wavelength, I_0 is the incident irradiation light intensity, $A_0(irr)$ is the initial absorption at the irradiation wavelength and l the light pathway ($l = 1\ cm$).

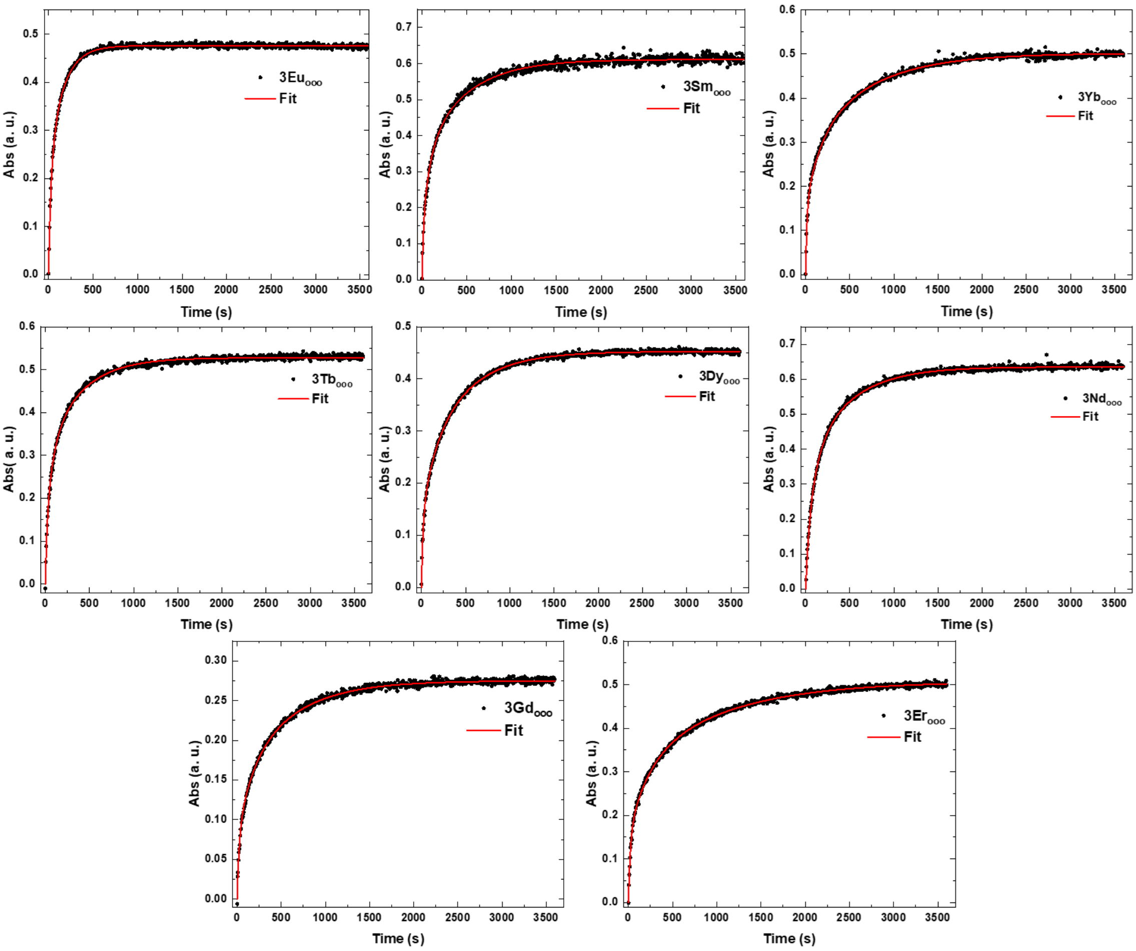

Figure S18. Individual absoprtion time-profiles of **3Ln$_{ooo}$** at the maximum of absorption of the corresponding closed isomers, upon irradiation at 365 nm, at T = 20°C.

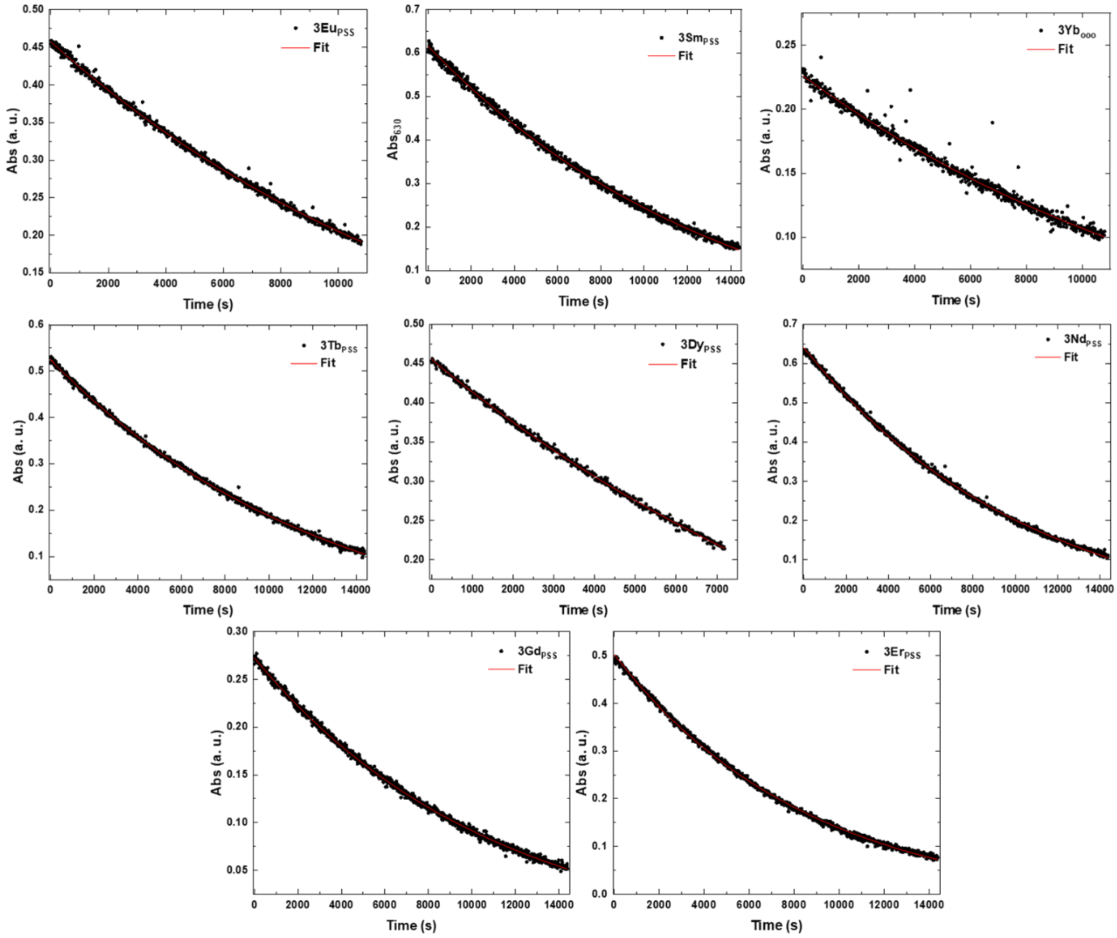

Figure S19. Individual absoprtion time-profiles of **3M$_{ooo}$** at the maximum of absorption of the corresponding closed isomers, upon irradiation at 577 nm, at T = 20°C.

	**3Eu**	**3Sm**	**3Yb**	**3Tb**	**3Dy**	**3Nd**	**3Gd**	**3Er**
C_0 (mol L^{-1})	1.2 x 10^{-5}	4.2 x 10^{-6}	1.4 x 10^{-5}	1.2 x 10^{-5}	1.1 x 10^{-5}	1.5 x 10^{-5}	7.3 x 10^{-6}	1.3 x 10^{-5}
I_0^{365} (mW)	1.14	1.15	1.15	1.11	1.10	1.16	0.96	1.18
I_0^{577} (mW)	0.44	0.68	0.44	0.58	0.58	0.60	0.52	0.60

Table S1. Experimental conditions of the photokinetic studies on **3M$_{ooo}$** derivatives

Transient absorption and emission spectroscopy

The nanosecond transient absorption spectroscopy setup (flash photolysis) has been described elsewhere.[2] Briefly a third harmonic pulse (355 nm) of a Nd:YAG laser with an output power of ca. 1 mJ and a pulse width of 7 ns was used as excitation beam and a pulsed Xe lamp was utilized as probe light with a 90° geometry. A spectroscopic cell (1cmx1cm) was used and the absorbance of diluted sample solutions were about 1 at 350 nm (1 cm length). One experiment is an average of 6 measurements and each pump pulse was separated by 2 seconds delay. To avoid accumulation of the closed-ring isomer, a CW LED (625nm, 700 mW, Thorlabs) was continuously irradiating the cuvette (90° respectively to probe light). It was checked that no change of signal was indunced by the LED light in the nanosecond microsecond time range. 10000 step delay points with a 10-microsecond time range were registered by the oscilloscope (no impedance was used and resolution is about 10 ns). Transient difference absorption spectra were obtained from the transient absorption decays recorded in steps of 10 nm wavelengths as absorbance changes for the given delay-times (Figure S20).

As already published previously,[3] femtosecond transient absorption spectra were measured using a commercial system (Ultrafast Systems, HELIOS) coupled with a Ti:sapphire amplifier (Coherent, Astrella, 800 nm, 60 fs FWHM, 1 kHz, 5 W)[4]. Here the instrument settings allow to measure time delay until 6 ns with a spectral range from 360 nm to 670 nm using CaF_2 to generate white light continuum probe light. The pump beam at 350 nm (300 nJ, 100 μm FWHM, 60 fs FWHM) is generated using an OPA (Coherent, OPerA Solo). The relative polarization of the excitation and probe pulses was set to the magic angle with respect to each other with a Berek compensator. The energy of the pump pulse was selected to be in the linear response range. Each acquisition is the average of 4 measurements with 2 s acquisition per time delay (250 points with logarithmic steps from -1 ps to 6 ns). The diluted sample solutions (spectroscopic grade dichloroethane) were placed into a flow Harrick cell (3 mL solution) combined with Micro annular gear pumps (HNP Mikrosysteme, 7255 model, Teflon tubing) with 1 mm CaF_2 windows and an internal thickness of 2 mm (absorbance about 0.8-1 at 350 nm) to refresh the probe volume between consecutive pump pulses each 1 millisecond. Due to cyclization conversion of the compounds by the pump pulse, the solutions were irradiated in an external reservoir with a CW LED (625nm, 700 mW, Thorlabs). Steady-state absorption spectra before and after the measurements detected no absorption band due to the closed-ring isomer, indicating that accumulation of the closed-ring isomer was negligible. Data were analyzed using Glotaran software (1.5.1) and the quality of the fit was evaluated by checking the residuals of the spectra for each time delay and for each wavelength kinetics. For the fit, negative time delay point (-10 ps to - 1 ps) were averaged and subtracted as background. In Glotaran, dispersion, coherent artifact and instrumental Gaussian pulse response function were convolved to the sum of a multiexponential function (Figure S20). Transient spectra were corrected from the GVD (about 1.5 ps between 360 and 670 nm) using Surface Explorer software.

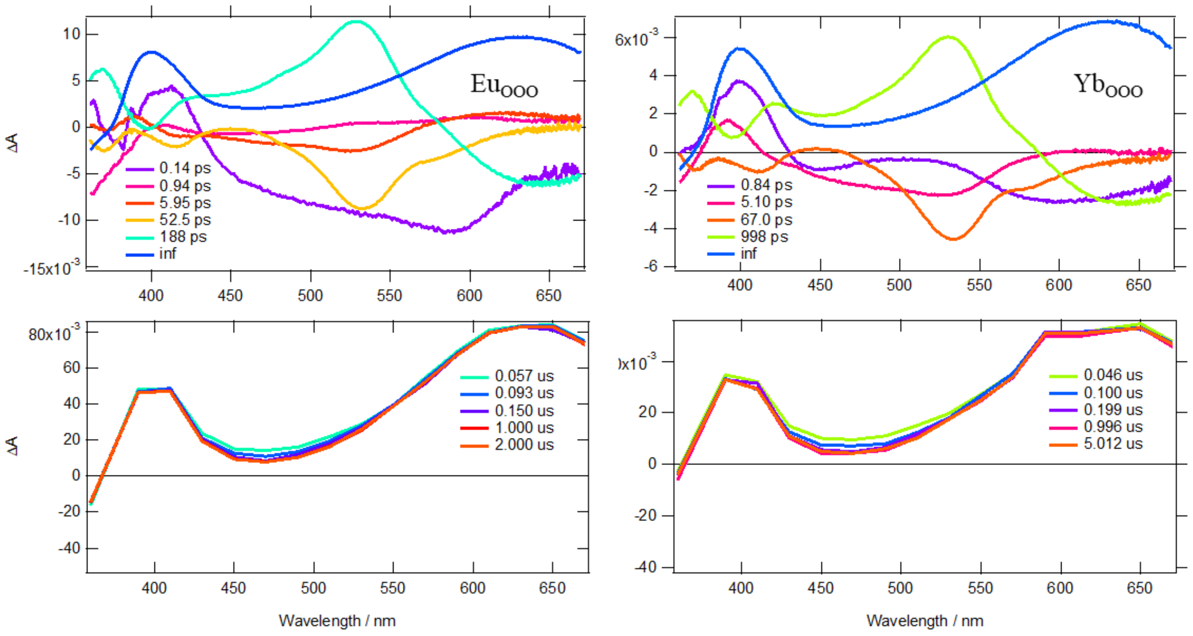

Figure S20: Decay associated spectra of the kinetics traces obtained from femtosecond transient absorption experiments for **$3Eu_{ooo}$ and $3Yb_{ooo}$** (top panels) and microsecond transient absorption spectra obtained after a 355 nm nanosecond excitation (bottom panels).

As recently described for the measurement of the luminescence of upconversion nanoparticles,[5] time resolved luminescence measurements for lanthanide were performed using a nanosecond 976 nm excitation (Nd:YAG coupled with an OPO, fwhm 7- 8 ns, 2.5 mJ, 2 Hz). A quartz cell (4 × 10 mm) was used and the emitted light was collected at 90°, dispersed by a spectrometer (iHR 320, center wavelength 980 nm) and analyzed with a gated intensified CCD camera (1024 x 256 pixels, PI-MAX 4, Princeton Instruments). Time-resolved spectra between 850 and 1050 nm were recorded with constant gate width of 500 ns. The first time gate was set to start at 0 µs and 100 gates were recorded until 50 µs. Ytterbium luminescence for **$3Yb_{ooo}$** was plotted in function of time by integrating the intensity signal between 940 - 990 nm for each time gate point (Figure S21). After reaching photostationary state using UV irradiation, no emission signal could be measured.

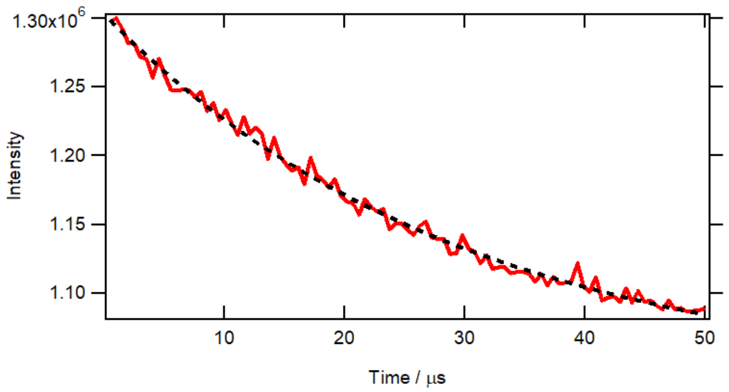

Figure S21: Time resolved emission integrated between 940 – 990 nm for **$3Yb_{ooo}$** after a nanosecond excitation at 976 nm.

Emission Spectroscopy Studies

Luminescence spectra were recorded with a Horiba-Jobin Ybon Fluorolog-3 ® spectrofluorimeter. Excitation light was provided by a Xe lamp and the excitation wavelength was set to the maximum of absorption of the DTE's open form (350 nm, absorbance ~0.31–0.33) by means of a grating monochromator and by using a UG11 band-pass filter (280–380 nm). Photoluminescence spectra in the near-infrared range were collected at a right angle with respect to the excitation beam with a liquid-nitrogen-cooled Symphony II® CCD array detector placed after an imaging spectrograph. For the experiments in the visible range the spectrofluorimeter was amended with a home-built setup. Briefly, the emission was collected in the forward direction by an off-axis collecting lens and sent to a fiber-coupled Avantes AvaSpec-ULS2048 grating spectrometer equipped with a CMOS linear sensor thereby providing a fast readout without the need for monochromator scanning. In both detection configurations, the residual excitation light was blocked by suitable low-pass color filters (450 nm). All the spectra presented in this study were corrected for the intensity response of the excitation lamp, gratings and detectors. For measurements at 293 K of solutions in dicholoromethane (CH_2Cl_2) the samples were placed in 1 cm square quartz cuvettes under continuous stirring (600 rpm), whereas experiments at 77 K of organic glass-forming solutions in 2-methyltetrahydrofurane (MeTHF) were performed in 4 mm O.D. quartz tubes placed in a liquid-nitrogen-filled quartz Dewar flask.

For reopening experiments the samples were irradiated for 5–10 minuntes with a 660 nm LED (M660, ThorLabs) under stirring (600 rpm).

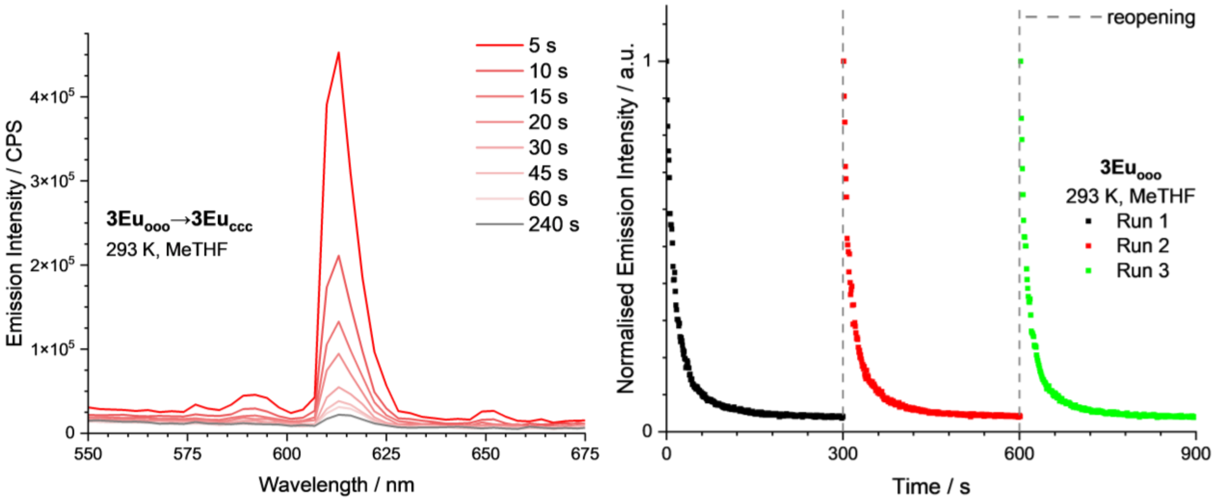

Figure S22. Emission spectra (left) and emission decrease at 613 nm (right) of **3Eu$_{ooo}$** in MeTHF at 293 K obtained upon continuous UV irradiation at λ_{ex} = 350 nm to PSS, [*c*]~10^{-5} mol.L^{-1}.cm^{-1}. The initial spectra were recovered after 293 K irradiation at λ = 660 nm between each decay (dashed grey lines).

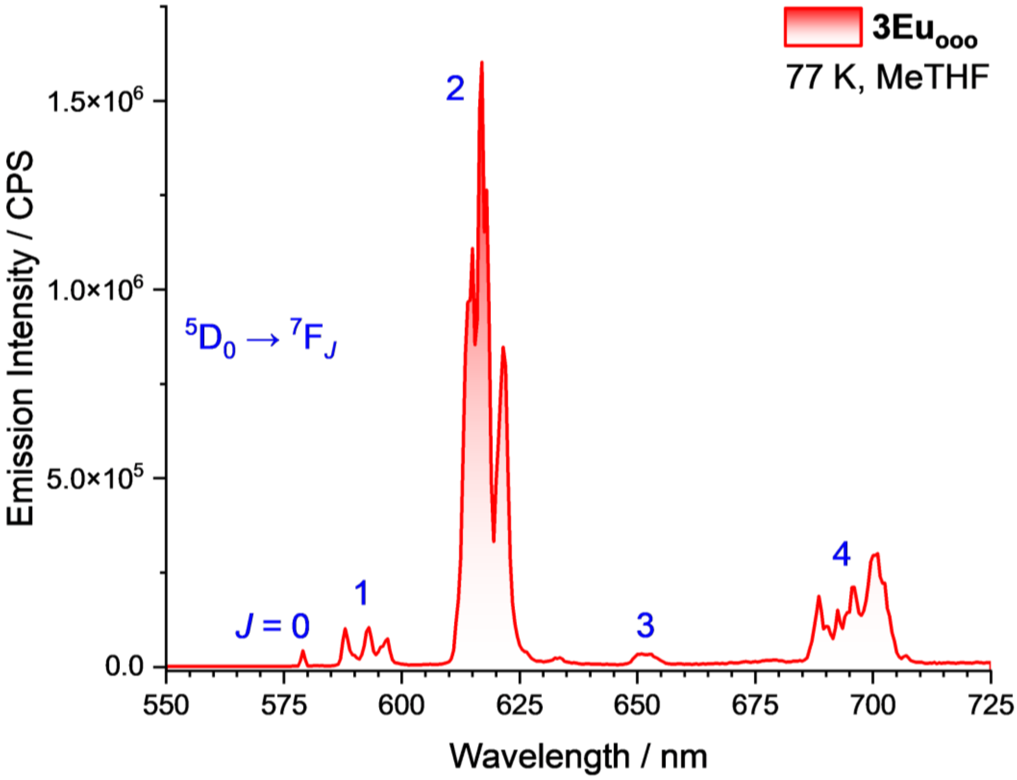

Figure S23. Emission spectrum of $\mathbf{3Eu_{ooo}}$ in MeTHF at 77 K (λ_{ex} = 350 nm, [*c*]~10^{-5} mol.L^{-1}.cm^{-1}).

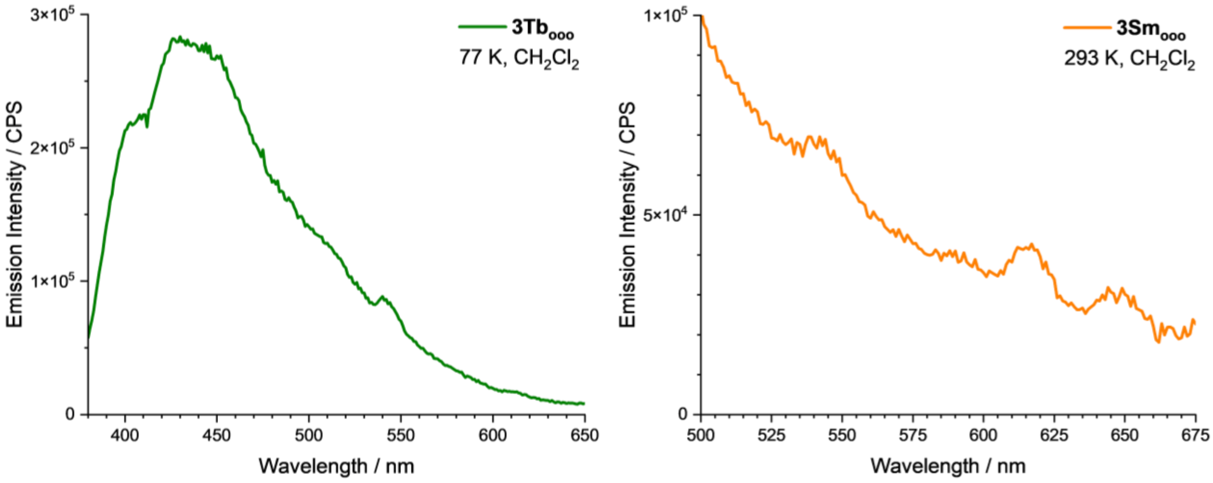

Figure S24. Emission spectra of $\mathbf{3Tb_{ooo}}$ (left) at 77 K and $\mathbf{3Sm_{ooo}}$ (right) at 293 K in CH_2Cl_2 (λ_{ex} = 350 nm, [*c*]~10^{-5} mol.L^{-1}.cm^{-1}).

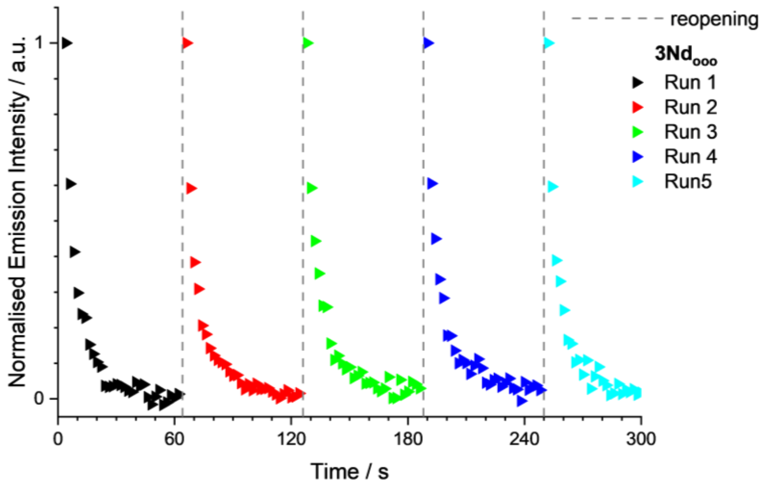

Figure S25. Emission decrease at 1350 nm for **3Nd$_{ooo}$** in CH_2Cl_2 solution at 293 K ([*c*]~10^{-5} mol.L^{-1}.cm^{-1}) obtained upon continuous UV excitation at λ_{ex} = 350 nm to PSS. The initial spectra were recovered after irradiation at λ = 660 nm between each decay (dashed grey lines).

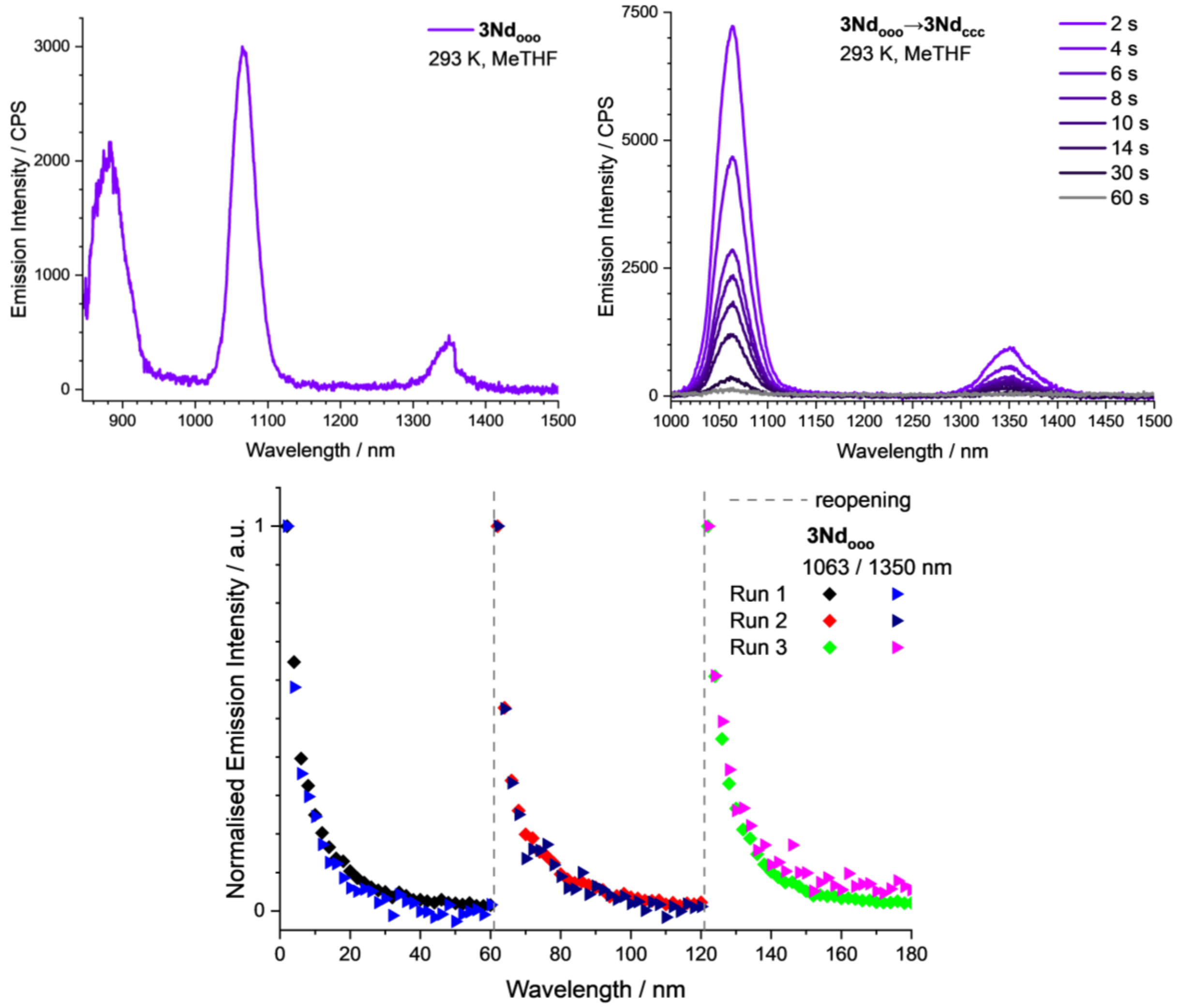

Figure S26. Full emission spectrum at t = 0 s (top left), emission spectra (top right) and emission decrease at 1063 and 1350 nm of **3Nd$_{ooo}$** in MeTHF at 293 K obtained upon continuous UV excitation at λ_{ex} = 350 nm to PSS, [*c*]~10^{-5} mol.L^{-1}.cm^{-1}. The initial spectra were recovered after irradiation at λ = 660 nm between each decay (dashed grey lines).

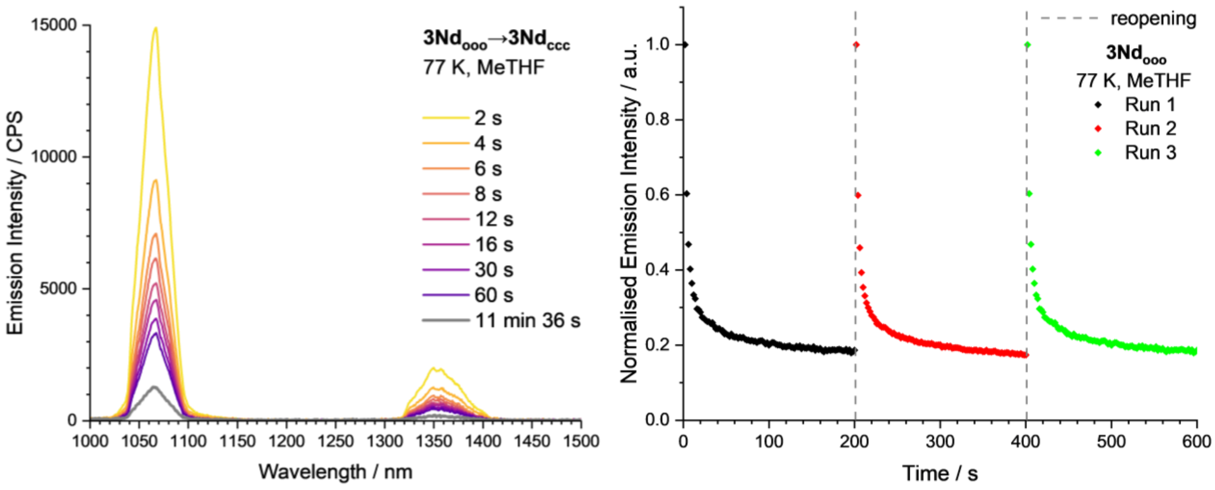

Figure S27. Emission spectra (left) and emission decrease at 1063 nm of **3Nd$_{ooo}$** in MeTHF at 77 K obtained upon continuous UV excitation at λ_{ex} = 350 nm to PSS, [c]~10^{-5} mol.L^{-1}.cm^{-1}. The initial spectra were recovered after thawing the frozen solutions and 293 K irradiation at λ = 660 nm between each decay (dashed grey lines).

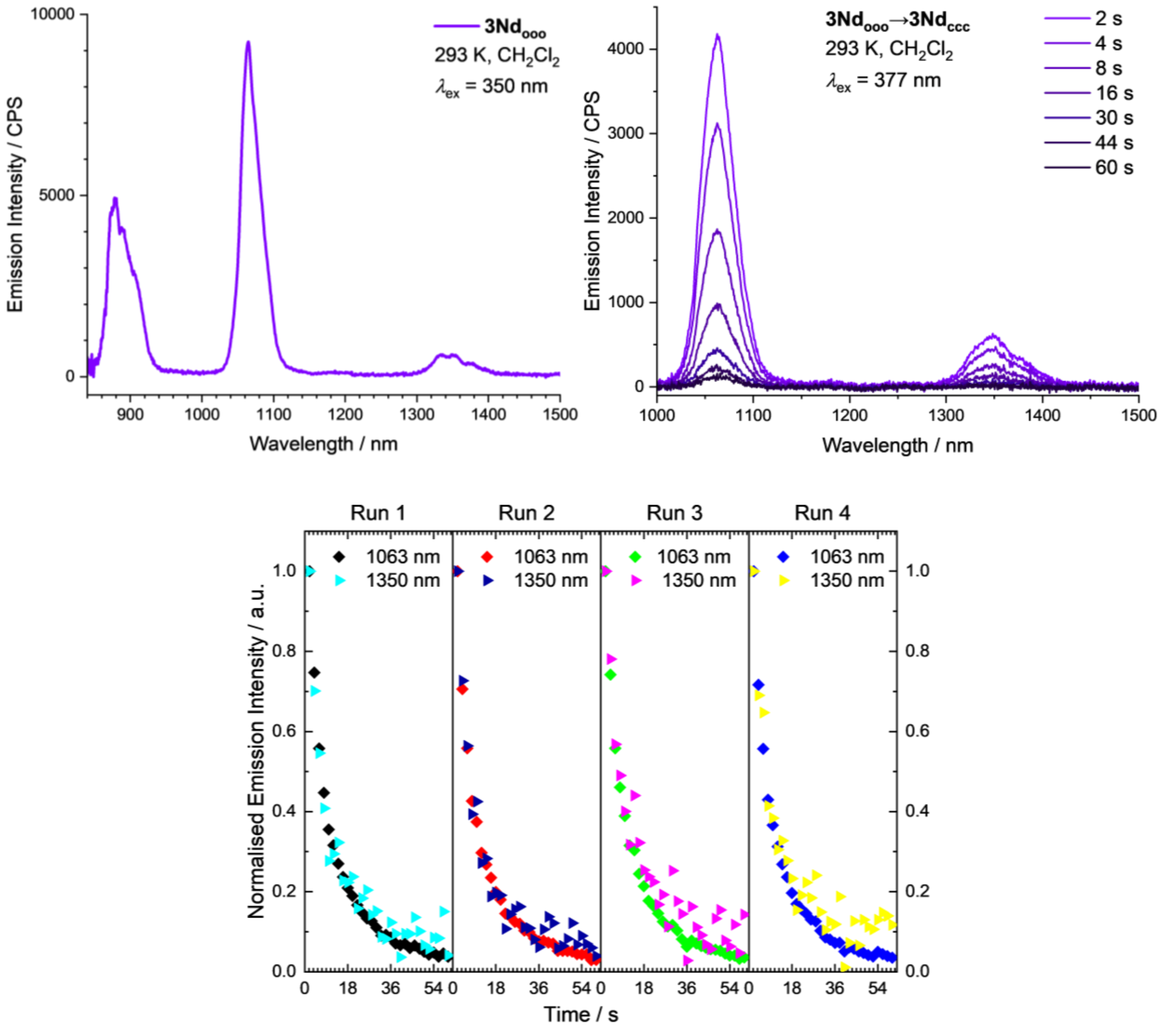

Figure S28. Full emission spectrum at t = 0 s (top left), emission spectra (top right) and emission decrease at 1063 and 1350 nm of **3Nd$_{ooo}$** in CH_2Cl_2 at 293 K obtained upon continuous UV excitation at isosbestic point λ_{ex} = 377 nm to PSS, [c]~10^{-5} mol.L^{-1}.cm^{-1}. The initial spectra were recovered after irradiation at λ = 660 nm between each decay (black solid lines).

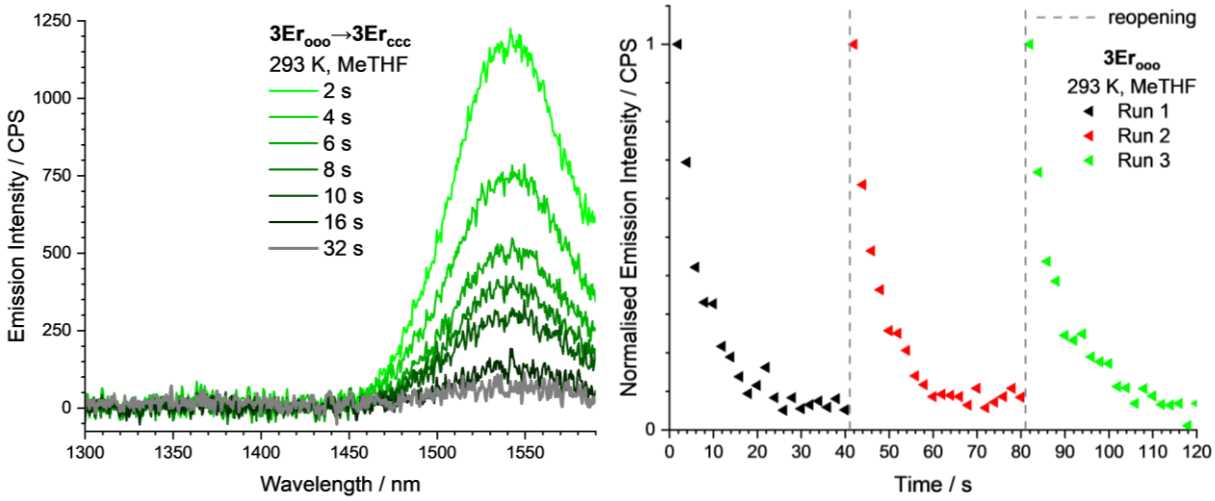

Figure S29. Emission spectra (left) and emission decrease at 1540 nm (right) of **3Er$_{ooo}$** in MeTHF at 293 K obtained upon continuous UV irradiation at λ_{ex} = 350 nm to PSS, [c]~10^{-5} mol.L^{-1}.cm^{-1}. The initial spectra were recovered after irradiation at λ = 660 nm between each decay (dashed grey lines).

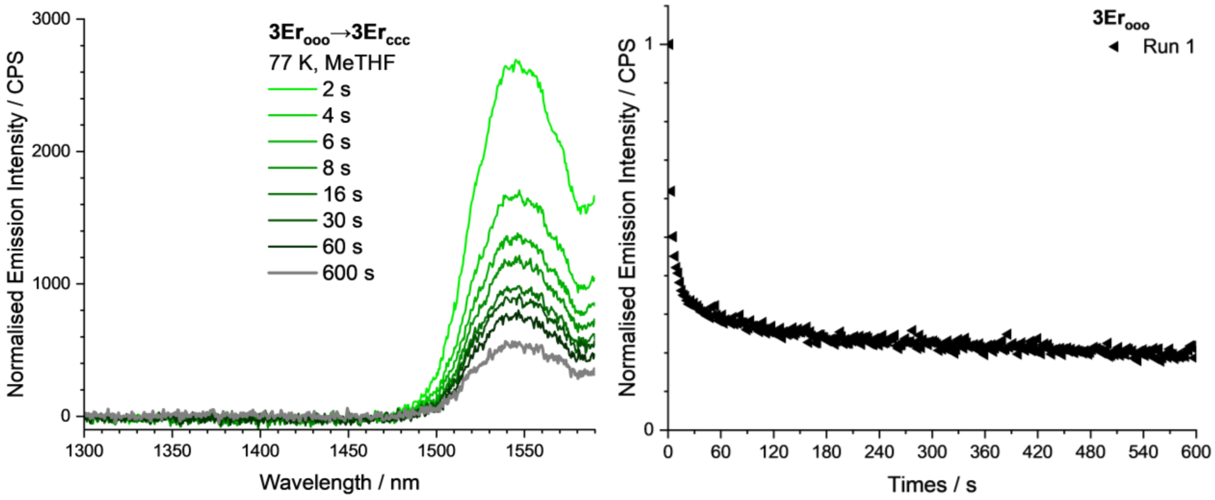

Figure 30. Emission spectra (left) and emission decrease at 1540 nm of **3Er$_{ooo}$** in MeTHF at 77 K obtained upon continuous UV excitation at λ_{ex} = 350 nm to PSS, [c]~10^{-5} mol.L^{-1}.cm^{-1}.

Crystallographic studies

General comments: ($C_{87}H_{50}DyF_{27}N_2O_6S_6$); M = 2087.15. A suitable crystal for X-ray diffraction single crystal experiment (blue stick, dimensions = 0.320 x 0.100 x 0.100 mm) was selected and mounted on the goniometer head of a D8 Venture (Bruker-AXS) diffractometer equipped with a CMOS-PHOTON70 detector, using Mo-Kα radiation (λ = 0.71073 Å, graphite monochromator) at T = 150(2) K. Crystal structure has been described in triclinic symmetry and P -1 (I.T.#2) centric space group (Rint = 0.0466). Cell parameters have been refined as follows: a = 11.6848(14), b = 18.141(2), c = 22.734(3) Å, α = 84.532(5), β = 82.784(4), γ = 77.108(4) °, V = 4649.0(10) $Å^3$. Number of formula unit Z is equal to 2 and calculated density d and absorption coefficient μ values are 1.491 $g.cm^{-3}$ and 1.043 mm^{-1} respectively. Crystal structure was solved by dual-space algorithm using SHELXT program,[6] and then refined with full-matrix least-squares methods based on F2 (SHELXL[7]). The contribution of the disordered solvents to the calculated structure factors was estimated following the BYPASS algorithm,[8] implemented as the SQUEEZE option in PLATON.[9] A new data set, free of solvent contribution, was then used in the final refinement. All non-Hydrogen atoms were refined with anisotropic atomic displacement parameters. H atoms were finally included in their calculated positions and treated as riding on their parent atom with constrained thermal parameters. A final refinement on F2 with 17626 unique intensities and 1317 parameters converged at ωRF2 = 0.1976 (RF = 0.0786) for 14634 observed reflections with $I > 2\sigma(I)$.
It is important to note that the diffraction patterns of the compound under study were of only moderate quality, with low resolution, reflecting strong disorder in the crystal structure. After extensive data collection and long exposition time (180 sec/deg), the quality of the diffraction dataset was nevertheless acceptable (Rint ~4.7), which allowed us to solve the skeleton of the crystal structure relatively quickly. However, significant disorder remained in different parts of the structure, making structure refinement challenging. To stabilize the refinement and reach convergence without non-positive ADP's (atomic displacement parameters), we had to apply several strong constraints on ADP's (EADP keyword) and restraints on interatomic distances (DFIX keyword) and stoichiometry (SUMP keyword). These steps allowed to obtain a qualitative refinement free from A-type and B-type alerts in the CHECKCIF procedure. Also, a residual electronic peak is observed near the heavy atom of the structure, i.e. dysprosium, in the difference Fourier map analysis, which is a common occurrence in this kind of molecular compound. This positive residual density, located approximately 0.9 A from the Dysprosium atom, could plausibly arise from imperfect absorption correction or unaccounted twinning.

CCDC 2402724 contains the supplementary crystallographic data for this paper. These data can be obtained free of charge from The Cambridge Crystallographic Data Centre via www.ccdc.cam.ac.uk/data_request/cif.

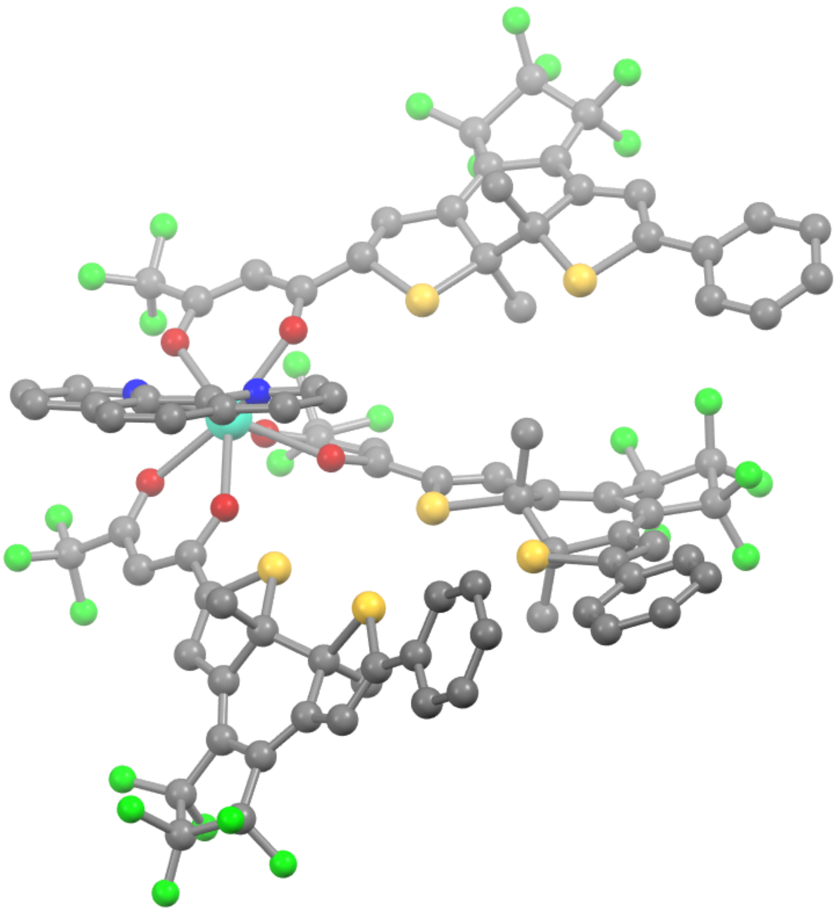

Figure S31 View of the SC- XRD structure of **3Dyccc** (H atoms are omitted for clarity, grey, yellow, blue, green and red spheres represent C, S, N, F and O atoms respectively).

Table S2. Crystal data and structure refinement parameters for **3Dy$_{ccc}$**.

	**3Dy$_{ccc}$**
Formula	$C_{87}H_{50}DyF_{27}N_2O_6S_6$
FW	2087.15
Cryst. Syst.	triclinic
Space group	P-1
a (Å)	11.6848(14)
b (Å)	18.141(2)
c (Å)	22.734(3)
α (°)	84.532(5)
β (°)	82.784(4)
γ (°)	
V (Å^3)	77.108(4)
Z	4649.0(10)
D_{ca} (g.cm^{-3})	2
T (K)	1.491
Final R (I > 2σ)	150(2)
R_w (all)	0.0786
	0.0947

Table S3. Selected bond lengths from **$3Dy_{ccc}$**.

**Bond**	**Bond length**
Dy1-N1	2.521(6)
Dy1-N2	2.548(5)
Dy1-O1	2.344(5)
Dy1-O2	2.280(5)
Dy1-O3	2.328(5)
Dy1-O4	2.336(5)
Dy1-O5	2.325(4)
Dy1-O6	2.304(5)
C20-C23	1.529(10)
C45-C47	1.530(13)
C70-C72	1.549(4)

Theoritical Calculations

The Amsterdam Density Functional (ADF[10,11,12]) software package was used to perform structural optimization of the investigated complexes. The ground state structures, namely **3Yb$_c$-S$_0$**, **3Yb$_o$-S$_0$** and **3Yb$_{o//}$-S$_0$** were optimized by using Kohn-Sham density functional theory (DFT), whereas geometries in their excited S_1 and T_1 states were obtained using the time-dependent DFT response calculations. These calculations utilized the scalar all-electron zeroth-order regular approximation (ZORA[13]). For the DFT optimizations, the spin-unrestricted formalism was used by considering doublet spin states, while the restricted formalism was employed for the TD-DFT calculations by replacing the open-shell Yb(III) ions by closed-shell Lu(III) ones. The hybrid functional PBE0[14,15] (Perdew-Burke-Ernzerhof) with 25% of exact exchange was employed along with the triple-ζ polarized Slater-type orbital (STO) all-electron basis set with one set of polarization functions for all atoms (TZP[16]). Solvent effects were considered by using the Conductor-Like Screening Model (COSMO) with the dielectric constant of 8.9 to model dichloromethane.[17] In these structural optimizations, only the DTE fragment and the H and F atoms of the whole molecules were relaxed, whereas the rest of the compounds was kept fixed. The nature of the transitions calculated at the TD-DFT level was analyzed with the help of the Natural Transition Orbitals (NTOs).[18] The NTOs were then visualized with the graphical user interface of ADF.

The multi-reference WFT calculations were performed with the help of the OpenMolcas software packages[19] on the DFT optimized geometries. In these calculations, the restricted active space self-consistent field[20] (RASSCF) approach was used to treat the static correlation effects. The second-order Douglas-Kroll-Hess[21,22,23,24] scalar relativistic (SR) Hamiltonian was used to treat the scalar relativistic effects in combination with the all-electron atomic natural orbital relativistically contracted (ANO-RCC) basis set from the Molcas library.[25,26,27] The basis sets were contracted to the triple-ζ plus polarization (TZP) quality for the Yb, N and O atoms (Yb = 25s22p15d11f4g2h/8s7p4d3f2g1h; N, O = 14s9p5d3f2g/4s3p2d1f), to the double-ζ plus polarization (DZP) quality for the C, F and S atoms (C, F = 14s9p4d3f2g/3s2p1d; S = 17s12p5d4f2g/4s3p1d) and to the double-ζ (DZ) quality for the H atoms (H = 8s4p3d1f/2s). The calculations were performed with a RAS[15,1,1,1,7,1]SCF active space. This RASSCF calculation was done on top of a CAS(13,7)SCF wave-function used to model the static correlation arising from the $4f^{13}$ Yb(III) ion. The RASSCF calculation was allowed to create (i) one hole in the RAS1 space containing a doubly occupied bonding molecular orbital centered on the DTE fragment, and (ii) one particle in the RAS3 space containing an unoccupied anti-

bonding molecular orbital or the DTE unit. The calculations employed the state-averaged formalism at the SR level by taking into account 7 quartets spin states corresponding to the combinations of triplet spin states on the ligand fragment $(\pi\text{-}\pi^*)^3$ and the doublet spin states of the Yb(III) ion (^{2}F). Additionally, 12 doublet spin states were also considered that correspond to the combinations of singlet spin states of the DTE fragment $(\pi\text{-}\pi^*)^1$ and the ^{2}F doublet spin states of the metal center. The influence of the dynamic electron correlation on the state energies were obtained from post-SCF multi-configurational pair-density functional theory (MC-pDFT) with the on-top ftrevPBE density functional.[28,29] The spin-orbit coupling (SOC) was then introduced within a state interaction among the basis of calculated SR states using the restricted active space state interaction (RASSI) approach.[30] Herein, the SOC matrix is diagonalized using the calculated 7 SR quartet and 12 SR doublet spin states. The nature of the molecular orbitals was analyzed using natural orbitals (NOs) that were directly obtained from the multi-configurational wavefunctions that include SOC effects. The procedure to obtain these orbitals is explained in Ref. [31, 32, 33] The orbitals were then visualized with the graphical interface of the ADF software package.

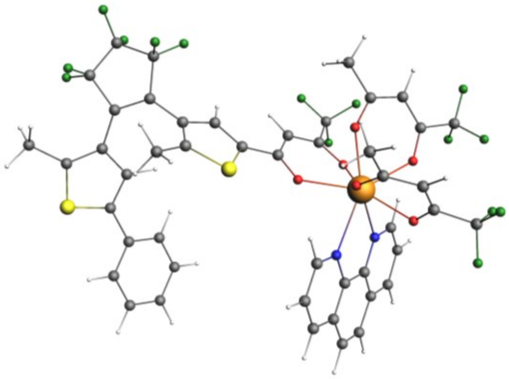

Figure S32. Representation of the optimized **3Yb$_{o//}$-S$_0$** model structure.

Table S4. (TD-)DFT calculated vertical (diabatic) and relaxed (adiabatic) relative energies (in eV) between the S$_0$, S$_1$ and T$_1$ states of the **3Yb$_c$**, **3Yb$_o$** and **3Yb$_{o//}$** model compounds.

	**3Yb$_c$**		**3Yb$_o$**		**3Yb$_{o//}$**	
	Vertical	Relaxed	Vertical	Relaxed	Vertical	Relaxed
S$_0$	0	0	0	0	0	0
S$_1$	1.837	1.389	3.561	2.440	3.611	2.563
T$_1$	1.068	0.362	2.724	2.372	2.680	2.332

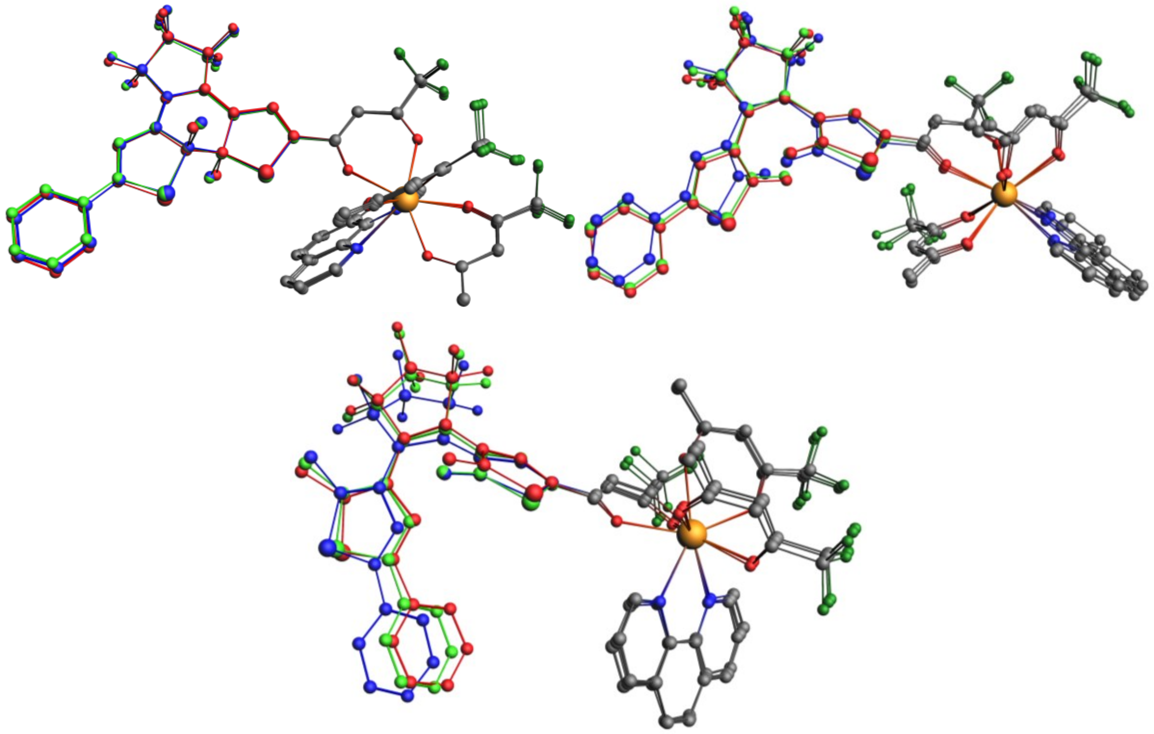

Figure S33. Overlay of the S_0 (red), S_1 (blue) and T_1 (green) DTE structures in the **3Yb$_c$** (top left), **3Yb$_o$** (top right) and **3Yb$_{o//}$** (bottom) model compounds.

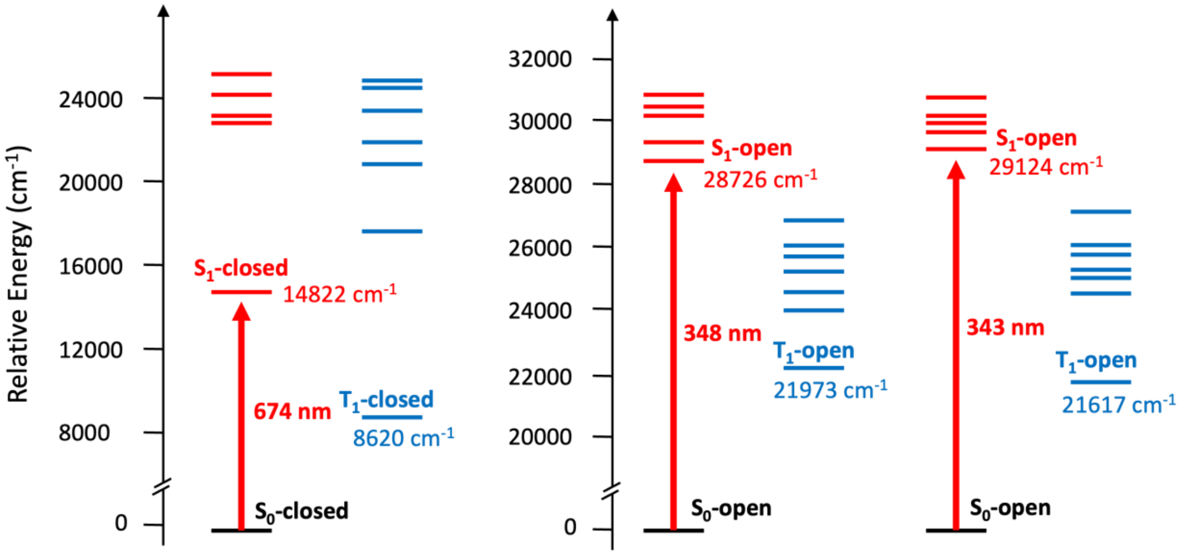

Figure S34. Calculated energy state diagrams at the TD-DFT level for **3Yb$_c$** (left), **3Yb$_o$** (middle) and **3Yb$_{o//}$** (right) with the ground state S_0 model structures. Note the change in the energy scale between the closed and open forms. Additional data are given in Table S5.

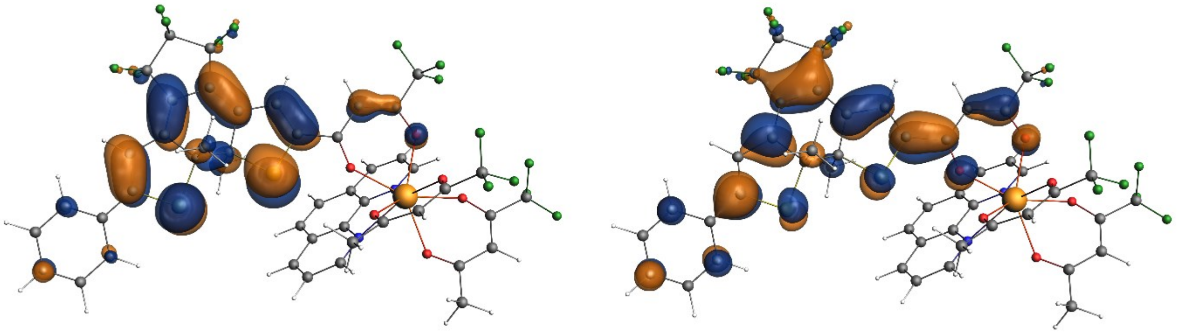

3Yb$_c$: $S_0 \rightarrow S_1$ 674 nm (*f* = 0.722)

NTO Occ. NTO Virt.

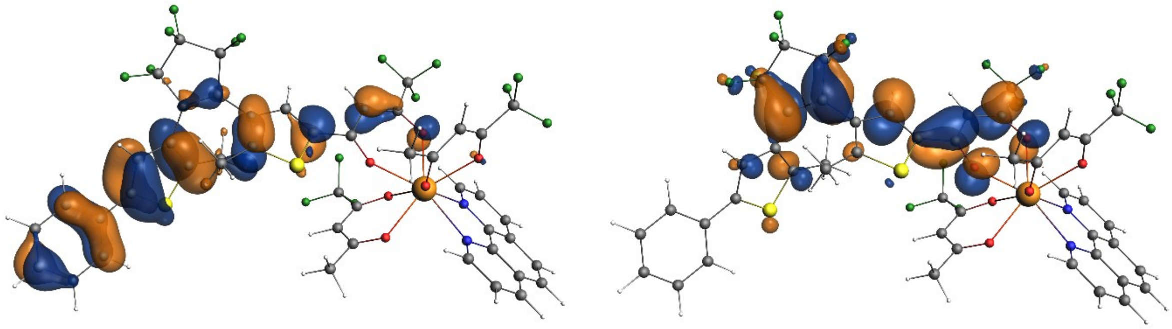

3Yb$_o$: $S_0 \rightarrow S_2$ 342 nm (*f* = 0.442)

NTO Occ. NTO Virt.

Figure S35. Plot of the natural transition orbitals (NTOs) associated to the lowest singlet to singlet transitions in **3Yb$_c$** (top) and **3Yb$_o$** (bottom). Iso-surface value = 0.03 au. The NTOs associated to the $S_0 \rightarrow S_1$ transition for **3Yb$_o$** look very similar but with smaller oscillator strength (see Figure S33).

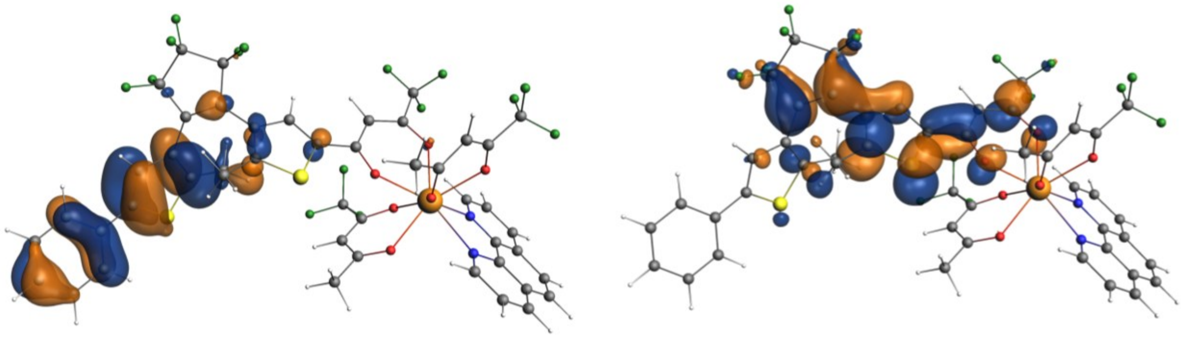

3Yb$_o$: $S_0 \rightarrow S_1$ 348 nm (*f* = 0.030)

NTO Occ. NTO Virt.

Figure S36. Plot of the natural transition orbitals (NTOs) associated to the lowest singlet to singlet transition and **3Yb$_o$**. Iso-surface value = 0.03 au.

Table S5. Calculated energy (eV / cm^{-1}), oscillator strengths (*f*) and assignment of the three lowest singlet and triplet transitions in the three model compounds at their S_0 geometries.

	State	Energy (eV / cm^{-1})	*f*	Main contribution	Assignment
**$3Yb_c$-S_0**	S_1	1.837 / 14816	0.722	H→L (97%)	DTE → DTE
	S_2	2.868 / 23131	0.008	H-2→L (38%)	DTE → DTE
				H→L+2 (25%)	DTE → DTE
	S_3	2.903 / 23414	0.019	H→L+1 (93%)	DTE → phen
	T_1	1.068 / 8614	-	H→L (96%)	DTE → DTE
	T_2	2.190 / 17663	-	H→L+2 (39%)	DTE → DTE
				H-2→L (36%)	DTE → DTE
	T_3	2.579 / 20801	-	H-1→L (23%)	hfac → DTE
				H-5→L (21%)	hfac → DTE
**$3Yb_o$-S_0**	S_1	3.561 / 28721	0.030	H→L+1 (54%)	DTE → DTE
				H→L+4 (33%)	DTE → DTE
	S_2	3.627 / 29253	0.442	H→L+4 (54%)	DTE → DTE
				H→L+1 (27%)	DTE → phen
	S_3	3.741 / 30173	0.014	H-1→L (49%)	hfac → DTE
				H-2→L+1 (13%)	hfac → DTE
	T_1	2.724 / 21970	-	H-1→L+1 (54%)	hfac → DTE
	T_2	2.967 / 23930	-	H→L+4 (49%)	DTE → DTE
	T_3	3.039 / 24511	-	H-3→L+6 (37%)	hfac → hfac
**$3Yb_{o//}$-S_0**	S_1	3.611 / 29124	0.053	H→L (57%)	DTE → hfac/phen
				H→L+1 (37%)	DTE → hfac/phen
	S_2	3.704 / 29874	0.517	H-1→L+1 (47%)	DTE → hfac/phen
				H→L+4 (21%)	DTE → DTE
	S_3	3.723 / 30028	0.058	H→L+4 (59%)	DTE → DTE
				H-1→L+1 (32%)	DTE → hfac/phen
	T_1	2.680 / 21615	-	H-1→L (50%)	DTE → hfac/phen
				H-1→L+1 (32%)	DTE → hfac/phen
	T_2	3.036 / 24486	-	H-2→L+5 (48%)	hfac → hfac
	T_3	3.089 / 24914	-	H→L+4 (56%)	DTE → DTE

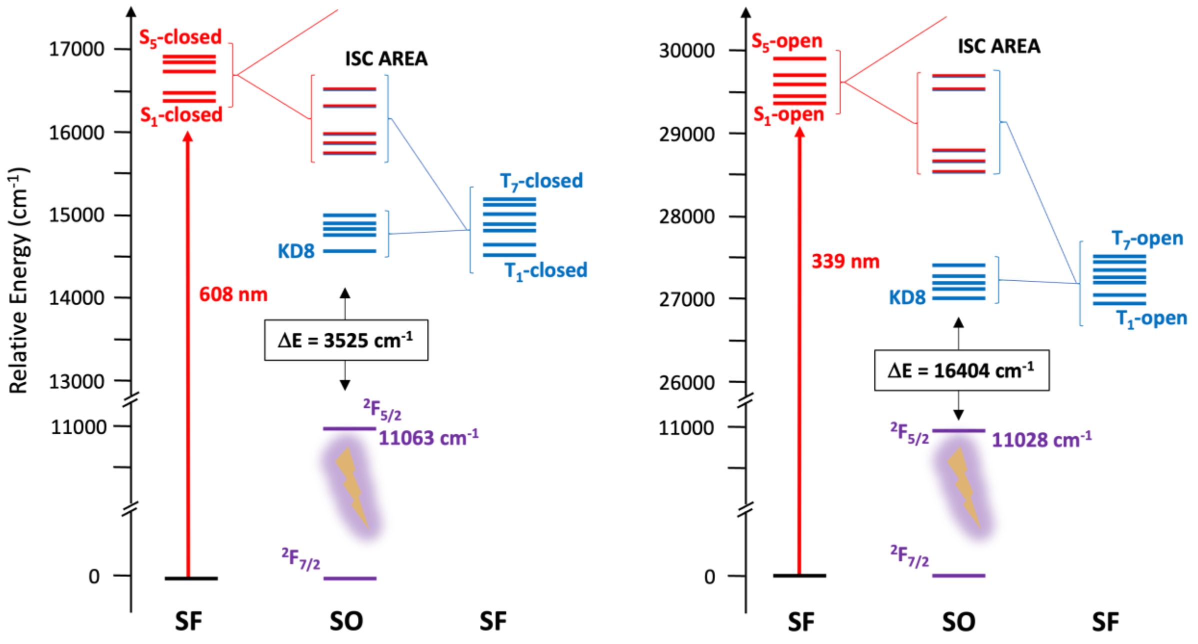

Figure S37. Calculated, at the RASSCF level, energy state diagrams (in cm^{-1}) of **3Yb$_c$-S$_0$** (left) and **3Yb$_o$-S$_0$** (right). Note the change in the energy scale between the two state diagrams. The state diagram of **3Yb$_{o//}$-S$_0$** is shown in Figure S35.

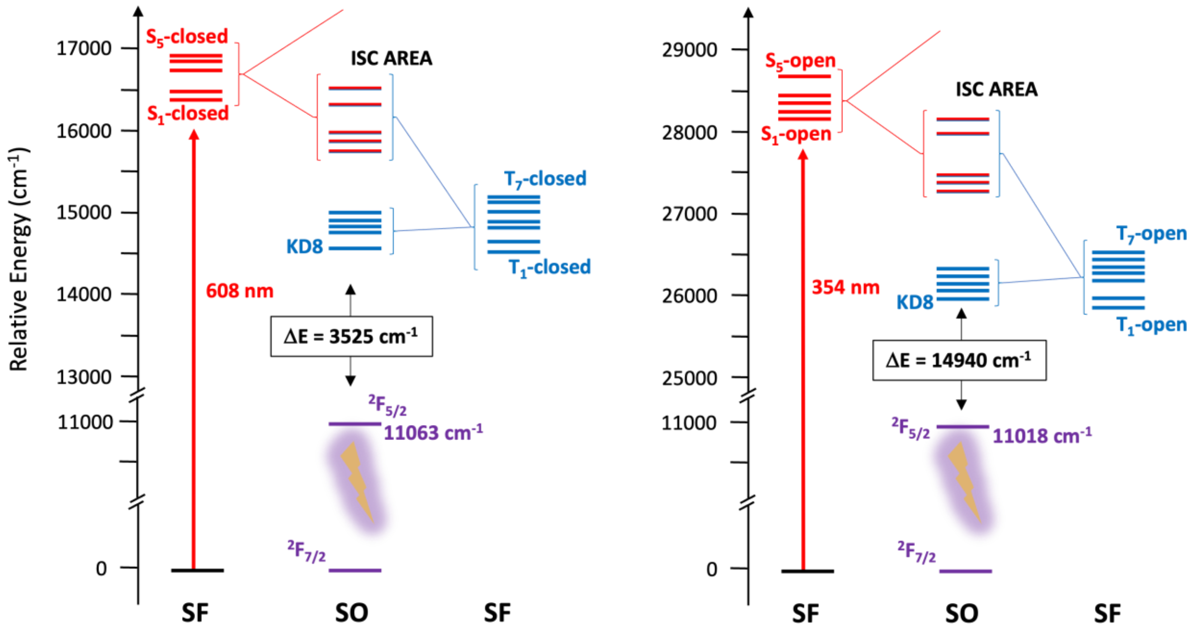

Figure S38. Calculated, at the RASSCF level, energy state diagrams (in cm^{-1}) of **3Yb$_c$-S$_0$** (left) and **3Yb$_{o//}$-S$_0$** (right). Note the change in the energy scale between the two state diagrams.

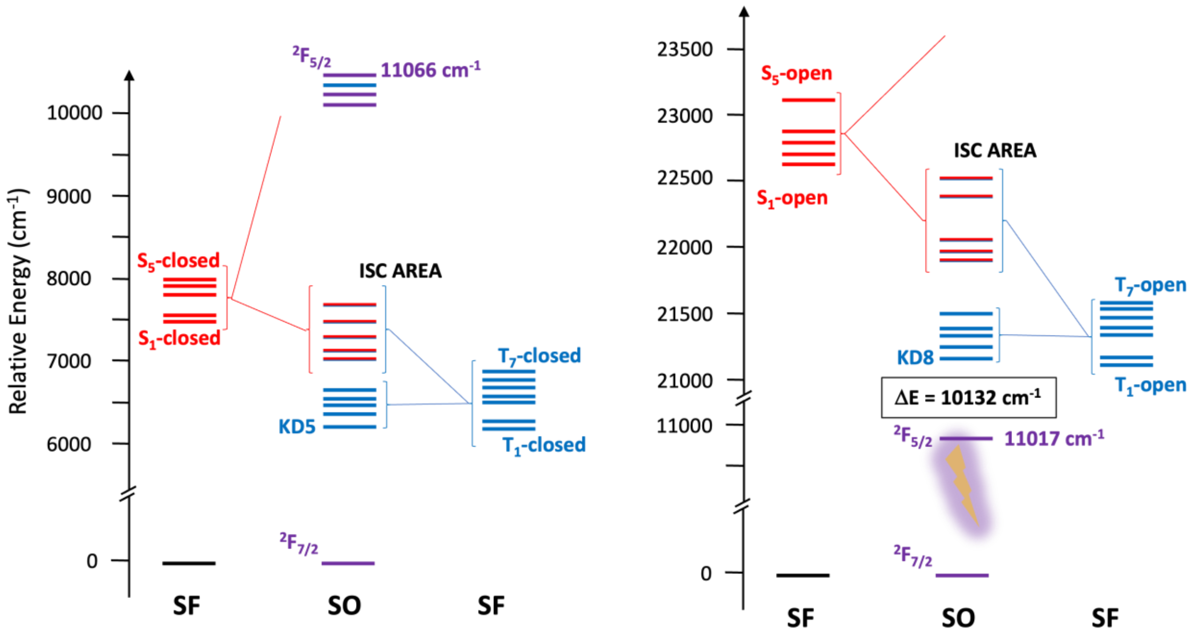

Figure S39. Calculated, at the RASSCF level, energy state diagrams (in cm^{-1}) of **3Yb$_c$-S$_1$** (left) and **3Yb$_{o//}$-S$_1$** (right). Note the change in the energy scale between the two state diagrams.

Table S6. Calculated relative energies (in cm^{-1}) of the different M_J states of the ground $^2F_{7/2}$ and excited $^2F_{5/2}$ terms of the Yb(III) center for the different model compounds. RAS[15,1,1,1,7,1]SCF level of calculations.

	**3Yb$_c$-S$_0$**	**3Yb$_c$-S$_1$**	**3Yb$_o$-S$_0$**	**3Yb$_{o//}$-S$_0$**	**3Yb$_o$-S$_1$**	**3Yb$_{o//}$-S$_1$**
$^2F_{7/2}$	0	0	0	0	0	0
	254	255	164	139	168	137
	436	438	401	381	405	381
	868	871	828	819	833	819
$^2F_{5/2}$	10356	10356	10345	10340	10345	10340
	10612	10614	10536	10511	10541	10510
	11063	11066	11028	11017	11033	11016

Table S7. Calculated relative spin-free energies (cm^{-1}) of the singlet and triplet spin states, and calculated relative spin-orbit energies (cm^{-1}) of the non-metal centered Kramers doublet (KD) states for the model compound **3Yb$_c$-S$_0$**. RAS[15,1,1,1,7,1]SCF results.

Spin-Free		Spin-Orbit		
State	ΔE	State	ΔE	Composition
T_1	14525	KD8	14588	45% T1, 42% T2, 4% T3, 3% T5, 2% T4
T_2	14638	KD9	14747	26% T1, 25% T4, 22% T2, 10% T5, 8% T7
T_3	14855	KD10	14833	30% T4, 22% T3, 15% T5, 9% T2, 8% T1
T_4	14902	KD11	14882	35% T3, 33% T5, 14 % T6, 9% T4, 5% T7
T_5	14935	KD12	14985	45% T7, 42% T6, 5% T5, 3% T2, 2% T4
T_6	15017	KD13	15759	18% T4, 17% S2, 14% T1, 14% S1, 10% T5
T_7	15066	KD14	15837	21% T2, 14% T3, 11% S3, 10% S1, 8% T1
S_1	16435	KD15	15879	16% T5, 14% T4, 13% S4, 13% S5, 9% T6
S_2	16465	KD16	16326	24% S9, 19% S8, 13% S3, 8% T3, 6% S5
S_3	16725	KD17	16528	25% S4, 22% S5, 18% S3, 6% T5, 6% T7
S_4	16862	KD18	19353	30% T7, 28% T6, 12% T3, 9% T5, 8% T4
S_5	16876			

Table S8. Calculated relative spin-free energies (cm^{-1}) of the singlet and triplet spin states, and calculated relative spin-orbit energies (cm^{-1}) of the non-metal centered Kramers doublet (KD) states for the model compound **3Yb$_c$-S$_1$**. RAS[15,1,1,1,7,1]SCF results.

Spin-Free		Spin-Orbit		
State	ΔE	State	ΔE	Composition
T_1	6168	KD5	6230	46% T1, 43% T2, 4% T3, 3% T5, 2% T4
T_2	6281	KD6	6383	28% T1, 23% T2, 20% T4, 9% T5, 8% T7
T_3	6498	KD7	6474	30% T4, 21% T3, 15% T5, 10% T2, 5% T7
T_4	6544	KD8	6524	36% T3, 33% T5, 14 % T6, 10% T4, 5% T7
T_5	6577	KD9	6626	44% T7, 41% T6, 5 % T5, 3% T2
T_6	6659	KD10	7086	19% T4, 18% S2, 16% S1, 11% T1, 10% T5
T_7	6708	KD11	7151	19% T2, 15% S1, 11% T3, 10% S2, 9% S3
S_1	7513	KD12	7297	16% T5, 14% S4, 14% T4, 12% S5, 9% T6
S_2	7544	KD13	7486	21% S2, 16% S3, 15% S1, 9% T3, 8% S5
S_3	7803	KD14	7679	26% S4, 24% S5, 16% S3, 6% T5, 6% T5
S_4	7940	KD17	10991	30% T7, 29% T6, 19% T5, 9% T1, 5% T4
S_5	7954			

Table S9. Calculated relative spin-free energies (cm^{-1}) of the singlet and triplet spin states, and calculated relative spin-orbit energies (cm^{-1}) of the non-metal centered Kramers doublet (KD) states for the model compound **3Yb$_o$-S$_0$**. RAS[15,1,1,1,7,1]SCF results.

Spin-Free		Spin-Orbit		
State	ΔE	State	ΔE	Composition
T_1	27380	KD8	27432	41% T1, 38% T2, 9% T4, 9% T3, 1% T5
T_2	27450	KD9	27545	23% T1, 22% T2, 18% T3, 14% T4, 12% T5
T_3	27639	KD10	27625	37% T3, 30% T4, 17% T5, 5% T2, 4% T7
T_4	27664	KD11	27668	30% T5, 21% T6, 14 % T4, 10% T7, 9% T1
T_5	27775	KD12	27783	46% T7, 42% T6, 6% T5, 2% T2, 1% T1
T_6	27819	KD13	29138	19% T3, 16% T4, 14% T1, 10% S2, 15% S1
T_7	27853	KD14	29196	19% T2, 17% T5, 9% T1, 11% S3, 8% S2
S_1	29514	KD15	29310	14% T4, 13% T3, 11% T6, 13% S4, 10% S5
S_2	29563	KD16	29706	24% S1, 19% S2, 17% S3, 6% T1, 6% T3
S_3	29699	KD17	29876	26% S5, 22% S4, 13% S3, 6% T6, 5% T3
S_4	29799	KD18	32162	30% T7, 28% T6, 19% T5, 8% T1, 5% T4
S_5	29999			

Table S10. Calculated relative spin-free energies (cm^{-1}) of the singlet and triplet spin states, and calculated relative spin-orbit energies (cm^{-1}) of the non-metal centered Kramers doublet (KD) states for the model compound **3Yb$_o$-S$_1$**. RAS[15,1,1,1,7,1]SCF results.

Spin-Free		Spin-Orbit		
State	ΔE	State	ΔE	Composition
T_1	20990	KD8	21036	42% T1, 40% T2, 8% T4, 7% T3, 1% T5
T_2	21057	KD9	21144	26% T1, 25% T2, 15% T3, 15% T4, 11% T5
T_3	21244	KD10	21229	38% T3, 31% T4, 14% T5, 5% T2, 4% T1
T_4	21269	KD11	21272	27% T5, 19% T6, 14 % T4, 10% T2, 10% T1
T_5	21360	KD12	21392	45% T7, 41% T6, 7% T5, 3% T1, 1% T2
T_6	21429	KD13	21759	18% T3, 18% S1, 16% T4, 11% S2, 8% T1
T_7	21466	KD14	21817	18% T5, 15% T2, 13% S3, 10% S2, 7% S1
S_1	22481	KD15	21931	16% S4, 13% T4, 11% T3, 11% S5, 10% T6
S_2	22529	KD16	22224	22% S1, 18% S3, 18% S2, 7% T3, 6% S4
S_3	22665	KD17	22394	29% S5, 21% S4, 12% S3, 7% T6, 6% T7
S_4	22764	KD18	25768	30% T7, 29% T6, 19% T5, 9% T1, 5% T4
S_5	22957			

Table S11. Comparison of the calculated relative spin-free energies (cm^{-1}) of the singlet and triplet spin states at the RASSCF and MC-pDFT levels.

	**3Yb$_c$-S$_0$**		**3Yb$_c$-S$_1$**		**3Yb$_o$-S$_0$**		**3Yb$_o$-S$_1$**	
State	ΔE RASSCF	ΔE MC-pDFT	ΔE RASSCF	ΔE MC-pDFT	ΔE RASSCF	ΔE MC-pDFT	ΔE RASSCF	ΔE MC-pDFT
T_1	14525	11886	6168	6725	27380	26713	20990	23099
T_2	14638	12184	6281	7158	27450	27309	21057	23520
T_3	14855	12548	6498	7548	27639	28307	21244	24227
T_4	14902	13502	6544	7908	27664	29358	21269	25544
T_5	14935	14237	6577	8533	27775	29575	21360	25587
T_6	15017	14254	6659	9171	27819	29680	21429	25595
T_7	15066	14427	6708	9323	27853	30819	21466	26503
S_1	16435	15477	7513	6821	29514	29481	22481	22927
S_2	16465	15568	7544	8183	29563	30849	22529	24274
S_3	16725	15718	7803	8560	29699	30893	22665	24284
S_4	16862	16065	7940	8608	29799	30974	22764	24326
S_5	16876	16274	7954	8746	29999	31140	22957	24499

Table S12. Comparison of the calculated spin-orbit coupling transition matrix elements (cm^{-1}) between the singlet and triplet spin states. RAS[15,1,1,1,7,1]SCF results.

	**$3Yb_c$-S_0**	**$3Yb_o$-S_0**	**$3Yb_c$-S_1**	**$3Yb_o$-S_1**
$<S_1\| H^{SO} \|T_1>$	430	1814	442	2205
$<S_1\| H^{SO} \|T_2>$	3303	1687	3391	1984
$<S_1\| H^{SO} \|T_3>$	1564	1878	1612	2228
$<S_1\| H^{SO} \|T_4>$	1222	1732	1257	2076
$<S_1\| H^{SO} \|T_5>$	1537	808	1574	1735
$<S_1\| H^{SO} \|T_6>$	912	855	931	1014
$<S_1\| H^{SO} \|T_7>$	1251	523	1287	611
$<S_2\| H^{SO} \|T_1>$	2971	1716	3051	2026
$<S_2\| H^{SO} \|T_2>$	521	2062	535	2505
$<S_2\| H^{SO} \|T_3>$	542	1517	545	1815
$<S_2\| H^{SO} \|T_4>$	2669	1244	2732	1498
$<S_2\| H^{SO} \|T_5>$	1599	1748	1658	2065
$<S_2\| H^{SO} \|T_6>$	572	688	587	840
$<S_2\| H^{SO} \|T_7>$	633	1070	653	1263
$<S_3\| H^{SO} \|T_1>$	445	612	458	712
$<S_3\| H^{SO} \|T_2>$	2313	2117	2377	2525
$<S_3\| H^{SO} \|T_3>$	1644	267	1690	292
$<S_3\| H^{SO} \|T_4>$	1888	2699	1944	3240
$<S_3\| H^{SO} \|T_5>$	2633	1850	2694	2215
$<S_3\| H^{SO} \|T_6>$	931	176	955	204
$<S_3\| H^{SO} \|T_7>$	399	198	421	233
$<S_4\| H^{SO} \|T_1>$	1873	1627	1919	1943
$<S_4\| H^{SO} \|T_2>$	297	624	285	730
$<S_4\| H^{SO} \|T_3>$	3002	2885	3081	3451
$<S_4\| H^{SO} \|T_4>$	1958	581	2019	694
$<S_4\| H^{SO} \|T_5>$	412	1406	426	1683
$<S_4\| H^{SO} \|T_6>$	1258	946	1259	1150
$<S_4\| H^{SO} \|T_7>$	1256	1060	1318	1267
$<S_5\| H^{SO} \|T_1>$	2134	2006	2195	2399
$<S_5\| H^{SO} \|T_2>$	1243	1484	1279	1780
$<S_5\| H^{SO} \|T_3>$	1109	1061	1122	1291
$<S_5\| H^{SO} \|T_4>$	1061	1460	1085	1723
$<S_5\| H^{SO} \|T_5>$	2106	362	2168	407
$<S_5\| H^{SO} \|T_6>$	2026	1970	2102	2334
$<S_5\| H^{SO} \|T_7>$	1662	1480	1682	1808
Average	1468	1321	1507	1598

The transition matrix element of the spin-orbit operator collected in Table S11 were obtained as followed:

$$\langle S_i | \hat{H}^{SO} | T_f \rangle = \sqrt{\sum_{f}^{g_f} m_{if}^2}$$

where m_{if} is a spin-orbit matrix element between the different spin components of the initial and final states, and where g_f is the degeneracy of the final state.

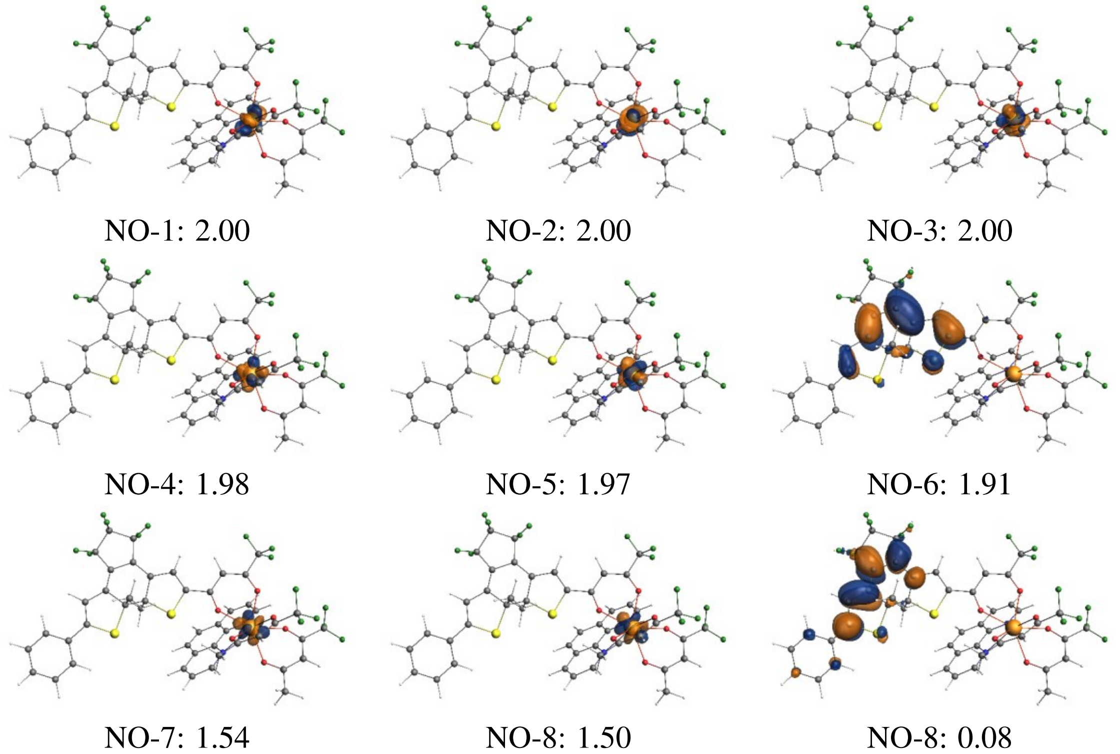

Figure S40. Plot and population of the natural orbitals for the SO GS of **3Yb$_c$-S$_0$**. RAS[15,1,1,1,7,1]SCF results. Iso-surface value: 0.03 au.

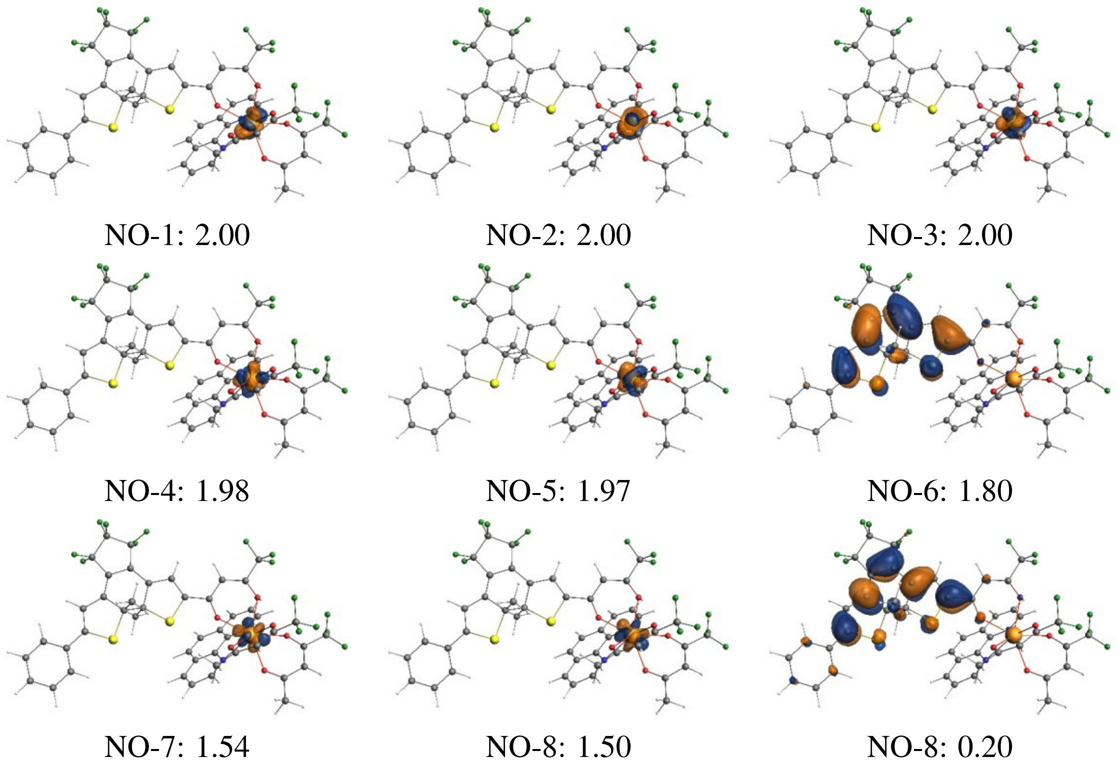

Figure S41. Plot and population of the natural orbitals for the SO GS of **3Yb$_c$-S$_1$**. RAS[15,1,1,1,7,1]SCF results. Iso-surface value: 0.03 au.

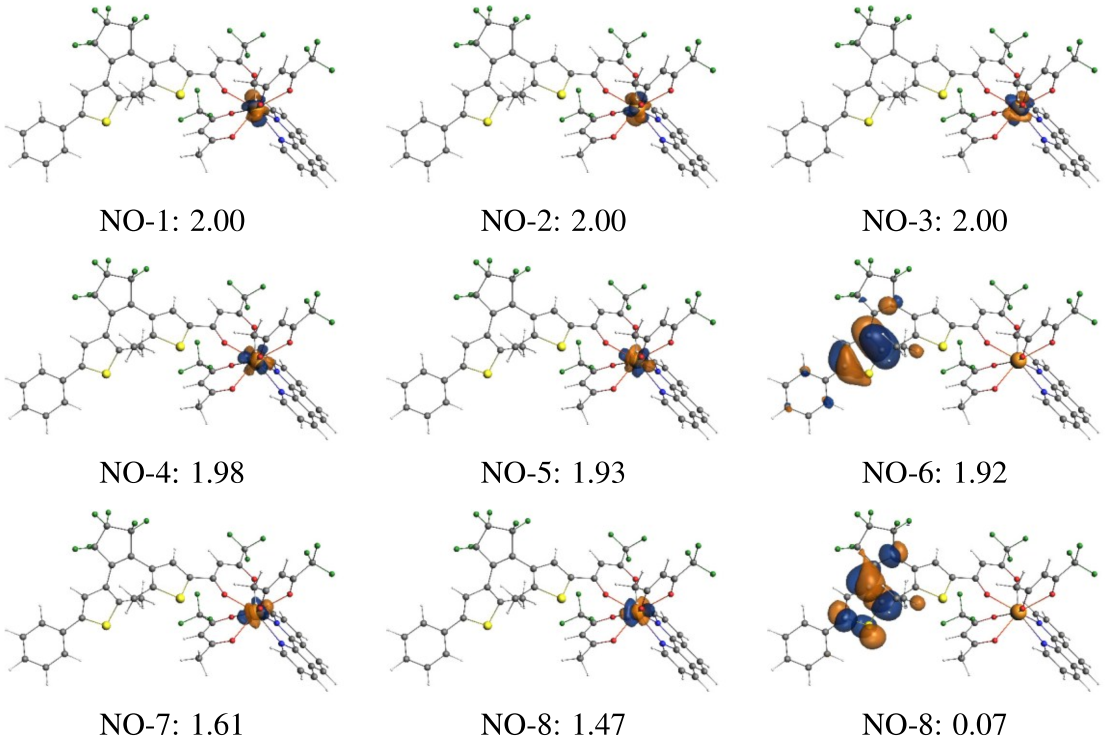

Figure S42. Plot and population of the natural orbitals for the SO GS of **3Yb$_o$-S$_0$**. RAS[15,1,1,1,7,1]SCF results. Iso-surface value: 0.03 au.

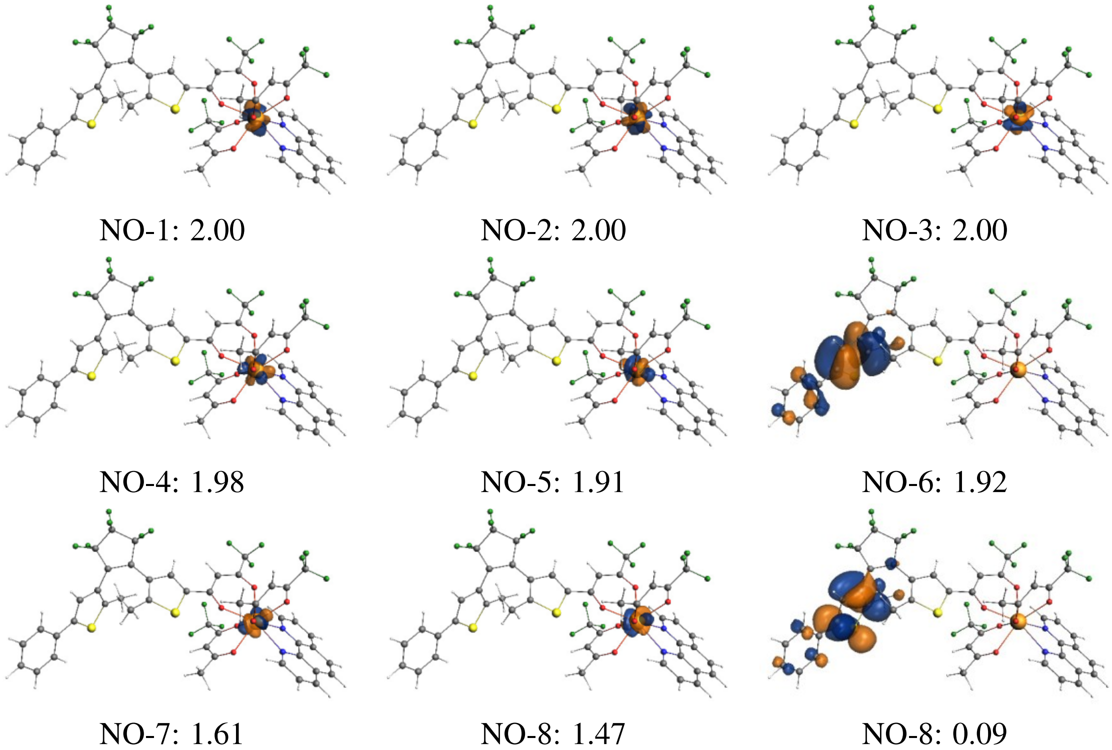

Figure S43. Plot and population of the natural orbitals for the SO GS of **3Yb$_o$-S$_1$**. RAS[15,1,1,1,7,1]SCF results. Iso-surface value: 0.03 au.

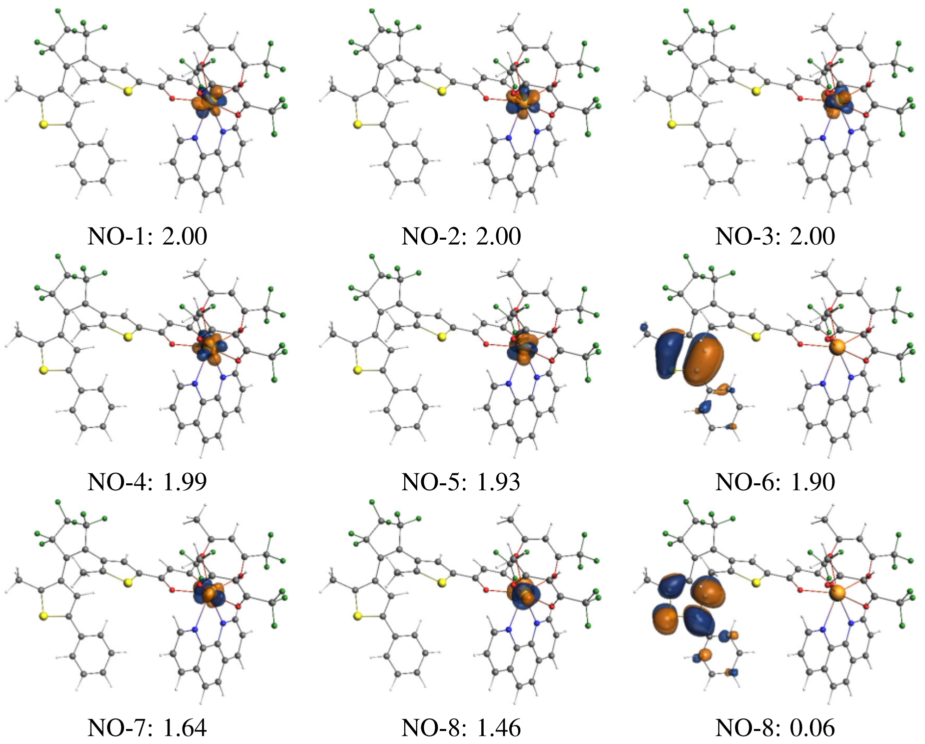

Figure S44. Plot and population of the natural orbitals for the SO GS of **3Yb$_{o//}$-S$_0$**. RAS[15,1,1,1,7,1]SCF results. Iso-surface value: 0.03 au.

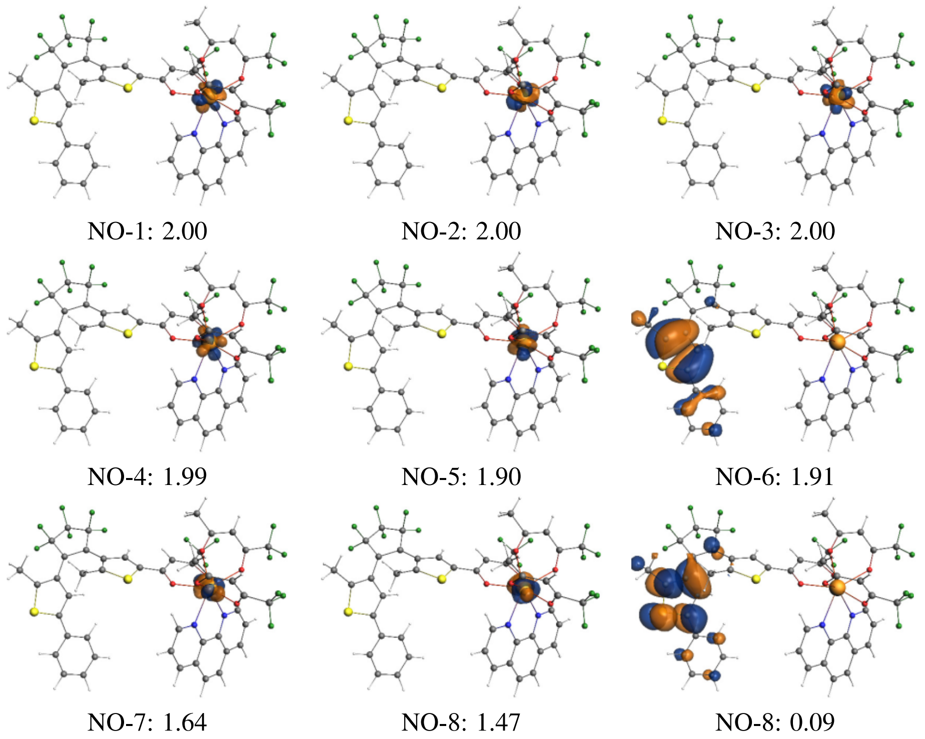

Figure S45. Plot and population of the natural orbitals for the SO GS of **3Yb$_{o//}$-S$_1$**. RAS[15,1,1,1,7,1]SCF results. Iso-surface value: 0.03 au.

1 Al Sabea, H.; Norel, L.; Galangau, O.; Hijazi, H.; Metivier, R.; Roisnel, T.; Maury, O.; Bucher, C.; Riobe, F.; Rigaut, S *J. Am. Chem. Soc.* **2019,** *141*, 20026. Al Sabea, H.; Norel, L.; Galangau, O.; Roisnel, T.; Maury, O.; Riobé, F.; Rigaut, S *Adv. Funct. Mater.* **2020,** *30*, 2002943.

2 Stoll, T.; Gennari, M.; Serrano, L.; Fortage, J.; Chauvin, J.; Odobel, F.; Rebarz, M.; Poizat, O.; Sliwa, M.; Deronzier, A.; Collomb, M.-N. *Chem. Eur. J.* **2013**, *19*, 782-792. Woodhouse, J.; Nass Kovacs, G.; Coquelle, N.; Uriarte, L. M.; Adam, V.; Barends, T. R. M.; Byrdin, M.; de la Mora, E.; Bruce Doak, R.; Feliks, M.; Field, M.; Fieschi, F.; Guillon, V.; Jakobs, S.; Joti, Y.; Macheboeuf, P.; Motomura, K.; Nass, K.; Owada, S.; Roome, C. M.; Ruckebusch, C.; Schirò, G.; Shoeman, R. L.; Thepaut, M.; Togashi, T.; Tono, K.; Yabashi, M.; Cammarata, M.; Foucar, L.; Bourgeois, D.; Sliwa, M.; Colletier, J.-P.; Schlichting, I.; Weik, M. *Nature Communications* **2020,** *11*, 741.

3 Suganuma, M.; Kitagawa, D.; Hamatani, S.; Sotome, H.; Mittelheisser, C.; Sliwa, M.; Ito, S.; Miyasaka, H.; Kobatake, S. *J. Mater. Chem. C* **2025,** *13*, 5259-5267.

4 See: https://www.ultrafast.systems/products/spectrometers-accessories/helios/

5 Zou, Q.; Marcelot, C.; Ratel-Ramond, N.; Yi, X.; Roblin, P.; Frenzel, F.; Resch-Genger, U.; Eftekhari, A.; Bouchet, A.; Coudret, C.; Verelst, M.; Chen, X.; Mauricot, R.; Roux, C. *ACS Nano* **2022,** *16*, 12107-12117.

6 Sheldrick, G. M. *Acta Cryst*. **2015**, *A71*, 3-8

7 Sheldrick, G.M., *Acta Cryst*. **2015**, *C71*, 3-8

8 Sluis, P. v.d.; Spek, A.L. *Acta Cryst.* **1990**, *A46*, 194-201

9 Spek, A. L. *J. Appl. Cryst.* **2003**, *36*, 7-13

10 te Velde, G.; Bickelhaupt, F. M.; Baerends, E. J.; van Gisbergen, S. J. A.; Fonseca Guerra, C.; Snijders, J. G.; Ziegler, T. Chemistry with ADF. *J. Comput. Chem.* **2001**, *22,* 931-967.

11 Fonseca Guerra, C.; Snijders, J. G.; te Velde, G.; Baerends, E. J. Towards an order-N DFT method. *Theor. Chem. Acc.* **1998**, *99,* 391.

12 Baerends, E. J.; Ziegler, T.; Atkins, A. J.; Autschbach, J.; Bashford, D.; Baseggio, O.; Bérces, A.; Bickelhaupt, F. M.; Bo, C.; Boerritger, P. M.; Cavallo, L.; Daul, C.; Chong, D. P.; Chulhai, D. V.; Deng, L.; Dickson, R. M.; Dieterich, J. M.; Ellis, D. E.; van Faassen, M.; Ghysels, A.; Giammona, A.; van Gisbergen, S. J. A.; Goez, A.; Götz, A. W.; Gusarov, S.; Harris, F. E.; van den Hoek, P.; Hu, Z.; Jacob, C. R.; Jacobsen, H.; Jensen, L.; Joubert, L.; Kaminski, J. W.; van Kessel, G.; König, C.; Kootstra, F.; Kovalenko, A.; Krykunov, M.; van Lenthe, E.; McCormack, D. A.; Michalak, A.; Mitoraj, M.; Mor- ton, S. M.; Neugebauer, J.; Nicu, V. P.; Noodleman, L.; Osinga, V. P.; Patchkovskii, S.; Pavanello, M.; Peeples, C. A.; Philipsen, P. H. T.; Post, D.; Pye, C. C.; Ramanantoan- ina, H.; Ramos, P.; Ravenek, W.; Rodríguez, J. I.; Ros, P.; Rüger, R.; Schipper, P. R. T.; Schlüns, D.; van Schoot, H.; Schreckenbach, G.; Seldenthuis, J. S.; Seth, M.; Snijders, J. G.; Solà, M.; M., S.; Swart, M.; Swerhone, D.; te Velde, G.; Tognetti, V.; Vernooijs, P.; Versluis, L.; Visscher, L.; Visser, O.; Wang, F.; Wesolowski, T. A.; van Wezenbeek, E. M.; Wiesenekker, G.; Wolff, S. K.; Woo, T. K.; Yakovlev, A. L. "ADF2017, SCM, Theoretical Chemistry, Vrije Universiteit, Amsterdam, The Netherlands, https://www.scm.com", **2017**.

13 van Lenthe, E.; Baerends, E. J.; Snijders, J. G. Relativistic Regular two-component Hamiltonians. *J. Chem. Phys.* **1993**, *99,* 4597-4610.

14 Ernzerhof, M.; Scuseria, G. E. Assessment of the Perdew-Burke-Ernzerhof Exchange-correlation Funtional. *J. Chem. Phys.* **1999**, *110,* 5029-5036.

15 Adamo, C.; Barone, V. Toward Reliable Density Functional Methods without Adjustable Paramaters: The PBE0 Model. *J. Chem. Phys.* **1999**, *110,* 6158-6170.

16 van Lenthe, E.; Baerends, E. J. Optimized Slater-type basis sets for the elements 1 – 118. *J. Comput. Chem.* **2003**, *24,* 1142-1156.

17 Pye, C.; Ziegler, T. An Implementation of the Conductor-Like Screening Model of Solvation within the Amsterdam Density Functional Package. *Theor. Chem. Acc.* **1999**, *101*, 396-408.

18 Martin, R. L. Natural Transition Orbitals. *J. Chem. Phys.* **2003**, *118*, 4775-4777.

19 Fdez. Galván, I.; Vacher, M.; Alavi, A.; Angeli, C.; Aquilante, F.; Autschbach, J.; Bao, J. J.; Bokarev, S. I.; Bogdanov, N. A.; Carlson, R. K.; Chibotaru, L. F.; Creutzberg, J.; Dattani, N.; Delcey, M. G.; Dong, S. S.; Dreuw, A.; Freitag, L.; Frutos, L. M.; Gagliardi, L.; Gendron, F.; Giussani, A.; González, L.; Grell, G.; Guo, M.; Hoyer, C. E.; Johansson, M.; Keller, S.; Knecht, S.; Kovačević, G.; Källman, E.; Li Manni, G.; Lundberg, M.; Ma, Y.; Mai, S.; Malhado, J. P.; Malmqvist, P. Å.; Marquetand, P.; Mewes, S. A.; Norell, J.; Olivucci, M.; Oppel, M.; Phung, Q. M.; Pierloot, K.; Plasser, F.; Reiher, M.; Sand, A. M.; Schapiro, I.; Sharma, P.; Stein, C. J.; Sørensen, L. K.; Truhlar, D. G.; Ugandi, M.; Ungur, L.; Valentini, A.; Vancoillie, S.; Veryazov, V.; Weser, O.; Wesołowski, T. A.; Widmark, P.-O.; Wouters, S.; Zech, A.; Zobel, J. P.; Lindh, R. OpenMolcas: From Source Code to Insight. *J. Chem. Theory Comput.* **2019,** *15*, 5925-5964.

20 Roos, B. O.; Taylor, P. R.; Siegbahn, P. E. M. A Complete Active Space SCF Method (CASSCF) using a Density Matrix Formulated Super-CI Approach. *Chem. Phys.* **1980**, *48,* 157–173.

21 Douglas, M.; Kroll, N. M. Quantum Electrodynamical Corrections to the Fine Structure of Helium. *Ann. Phys.* **1974**, *82,* 89-155.

22 Hess, B. A. Applicability of the no-pair equation with free-particle projection operators to atomic and molecular structure calculations. *Phys. Rev. A* **1985**, *32,* 756-763.

23 Hess, B. A. Relativistic electronic-structure calculations employing a two-component no-pair formalism with external-field projection operators. *Phys. Rev. A* **1986**, *33,* 3742-3748.

24 Wolf, A.; Reiher, M.; Hess, B. A. The generalized Douglas-Kroll transformation. *J. Chem. Phys.* **2002**, *117,* 9215-9226.

25 Widmark, P.-O.; Malmqvist, P.-Å.; Roos, B. O. Density-matrix averaged atomic natural orbital (ANO) basis-sets for correlated molecular wave-functions. I. First row atoms. *Theor. Chim. Acta* **1990**, *77,* 291–306.

26 Roos, B. O.; Lindh, R.; Malmqvist, P.-Å.; Veryazov, V.; Widmark, P.-O. Main group atoms and dimers studied with a new relativistic ANO basis set. *J. Phys. Chem. A* **2004**, *108,* 2851–2858.

27 Roos, B. O.; Lindh, R.; Malmqvist, P.-Å.; Veryazov, V.; Widmark, P.-O. New relativistic ANO basis sets for transition metal atoms. *J. Phys. Chem. A* **2005**, *109,* 6575.

28 Gagliardi, L. Truhlar, D. G.; Li Manni, G.; Carlson, R. K.; Hoyer, C. E.; Bao, J. L. Multiconfiguration Pair-Density Functional Theory: A New Way to Treat Strongly Correlated Systems. *Acc. Chem. Res.* **2017**, *50*, 66-73.

29 Zhou, C.; Gagliardi, L.; Truhlar, D. G. Multiconfiguration Pair-Density Functional Theory for Iron Porphyrin with CAS, RAS, and DMRG Active Spaces. *J. Phys. Chem. A.* **2019**, *123*, 3389-3394.

30 Malmqvist, P.-A.; Roos, B. O.; Schimmelpfennig, B. The restricted active space (RAS) state interaction approach with spin-orbit coupling. *Chem. Phys. Lett.* **2002**, *357,* 230-240.

31 Gendron, F.; Páez-Hernández, D.; Notter, F.-P.; Pritchard, B.; Bolvin, H.; Autschbach, J. Magnetic properties and electronic structure of neptunyl(VI) complexes: Wavefunctions, orbitals, and crystal-field models. *Chem. Eur. J.* **2014**, *20,* 7994–8011.

32 Gendron, F.; Pritchard, B.; Bolvin, H.; Autschbach, J. Single-Ion 4f Element Magnetism: an Ab-Initio Look at $Ln(COT)_2^-$. *Dalton Trans.* **2015**, *44,* 19886– 19900.

33 Autschbach, J. Orbitals for Analyzing Bonding and Magnetism of Heavy-Metal Complexes. *Comments Inorg. Chem.* **2016**, *36,* 215–244.
